# Spatiotemporal epidemiology and clinical manifestations of two decades of scrub typhus in India: a systematic review and meta-analysis

**DOI:** 10.1136/bmjgh-2025-018998

**Published:** 2025-08-03

**Authors:** Rini Chaturvedi, Syed Shah Areeb Hussain, Hayavadhan Sampath, Manju Rahi, Bijay R Mirdha, Amit Sharma

**Affiliations:** 1 Microbiology, All India Institute of Medical Sciences, New Delhi, India; 2 International Centre for Genetic Engineering & Biotechnology, New Delhi, India; 3 Btech Biotechnology, Vellore Institute of Technology, Vellore, Tamil Nadu, India; 4 Division of Epidemiology and Communicable Diseases, Indian Council of Medical Research, New Delhi, India; 5 Molecular Medicine, International Centre For Genetic Engineering and Biotechnology, New Delhi, India

**Keywords:** Epidemiology, Public Health, India, Rickettsia infections

## Abstract

**Background:**

Scrub typhus, once known as tsutsugamushi fever and attributed to Rickettsia, has transformed into a growing public health concern. Despite its increasing incidence in India, comprehensive spatiotemporal analyses of scrub typhus have been lacking.

**Methods:**

This study examines the prevalence of scrub typhus cases reported from 2003 to 2023, using available literature to provide a breakdown of cases by year and state in India, aiming to elucidate the disease’s spatiotemporal dynamics. The aetiological association of *Orientia tsutsugamushi* and scrub typhus is based on geographical, immunological and molecular genetic studies.

**Findings:**

This analysis identified 47 650 cumulative cases of scrub typhus in India over the past two decades. The case fatality rate was 5% out of 35 243 cases. Variations in transmission dynamics and the Leptotrombidium vector’s competence may influence the disease’s distribution. Nonetheless, there has been a notable increase in infections since 2010, peaking in 2019 and 2022. Curtailing and containing such an upsurge can be daunting and requires an interdisciplinary public health approach. Further, there is heterogeneity in studies on general, gastrointestinal, pulmonary and inflammatory symptoms compared with studies on cardiac, hepatic, neurological and other symptoms. The Weil-Felix test was the most common diagnostic technique used, and doxycycline was the treatment for scrub typhus cases.

**Interpretations:**

This meta-analysis can help policy-makers and researchers in India develop scrub typhus management and control policies.

**PROSPERO registration number:**

CRD42024611771.

**WHAT IS ALREADY KNOWN ON THIS TOPIC**

- Scrub typhus was sporadically reported in India, and multiple outbreaks occurred from the early to mid-1990s. However, some studies reported a decline in prevalence in the late 1990s. This disease, causing various complications and morbidity, has now re-emerged as a potential threat to public health in India.

**WHAT THIS STUDY ADDS**

- This systematic review and meta-analysis examined year-wise and location-wise data on the prevalence of cases, symptoms, complications, outbreaks and fatalities between 2003 and 2023. To date, only one study has reported on the burden of scrub typhus in India, while others have shown the burden of scrub typhus state-wise for particular years. This study provides the first spatiotemporal analysis of scrub typhus spread over two decades. There is an apparent change in the epidemiology of this disease. We have collated comprehensive geographical data along with symptoms and treatments here to reveal a complicated yet tractable public health problem in India.

**HOW THIS STUDY MIGHT AFFECT RESEARCH, PRACTICE OR POLICY**

- There was an unexpected upsurge of scrub typhus cases from 2010 onwards within the country. Multiple states and territories started to report the cases, and the recorded cumulative cases mounted to 39 124 from 2012 to 2022. Mizoram is the most recent state to implement diagnostic measures and intervention strategies to manage scrub typhus and has recorded as many as 20 558 cases. Other endemic states should also implement such control and diagnostic measures to estimate the actual burden of scrub typhus. To mitigate the rising scrub typhus burden in India, public health interventions must focus on awareness, screening, and intervention strategies, such as the containment of modifiable risk factors, timely referral, and the availability of cost-effective infrastructure, including case registries for updating central surveillance units. Further, combination therapy with doxycycline and azithromycin should be administered to counteract the problem of morbidity and mortality in patients.

## Introduction

 Scrub typhus is transmitted by the larval stage of the mites (Chiggers) from the Trombiculidae family and is caused by the intracellular obligate bacterium *Orientia tsutsugamushi*. Even though the disease is known as ‘scrub’ typhus, there is insufficient information to conclusively connect this ambiguous habitat category to the risk of human infection.^1^ There are high-risk regions in endemic areas, probably linked to the existence of suitable habitats for chigger species which carry bacteria. Our current understanding of such locations and their dynamics across time is restricted. The likelihood of a human infection is contingent on the degree of chigger exposure. In turn, exposure is influenced by environmental elements that affect chigger abundance such as temperature, humidity and vegetation. Behavioural aspects of human activities that are linked to the danger of chigger infestation include agricultural practices and exposure to forested regions.

The World Health Orgnazation (WHO) has designated scrub typhus as one of the world’s most underdiagnosed/under-reported diseases that frequently requires hospitalisation.^1^ The disease is linked to a high fatality rate, and around a million cases of scrub typhus in Southeastern Asia are reported yearly,^2^ while one billion are at risk of scrub typhus.^3^ Geographically limited to South and Southeast Asia, Northern Australia and the islands in the Indian and Pacific Oceans, the ‘tsutsugamushi triangle’ region is believed to be endemic to disease.^4^ However, new reports of scrub typhus from the Arabian Peninsula, Africa, Chile and Peru indicate that infection may have spread further.^5^

Scrub typhus is still a neglected disease despite a significant rise in cases in recent years. It presents a wide array of clinical manifestations, thereby making diagnosis complicated. While epidemiological studies on regional outbreaks and case reports from different parts of India are available, there is a paucity of data on the overall distribution of scrub typhus across India, making it difficult to understand the current status. Furthermore, as many as 68 different types of clinical manifestations of scrub typhus have been reported in the literature, with varying prevalence levels. Understanding the spatiotemporal changes in the spread of scrub typhus across India can aid in developing more effective management and control strategies. A clear understanding of the prevalence of different clinical manifestations is critical for early detection and prompt response. Therefore, in this meta-analysis, we aimed to collate data on the spatiotemporal spread of scrub typhus in India. We provide a comprehensive perspective of the epidemiological changes in the disease over the past two decades. We note the variance in symptoms and complications of scrub typhus as reported in the literature. This analysis would be valuable in helping the national disease control programme to develop strategies for detecting, combating and reducing scrub typhus in India.

### Etiological agent, vector and pathogenic factors

The agent for scrub typhus, *O. tsutsugamushi*, is a gram-negative, non-motile, non-capsulated pleomorphic bug lacking lipopolysaccharides and peptidoglycans.^6^ Studies across India have reported a wide variation in circulating genotypes of scrub typhus: Gilliam-like strains were reported from Bihar,^7^ and Shimla reported an equal proportion of Karp and Kato-like strains. At the same time, Meghalaya and Tamil Nadu had a preponderance of Kato-like strains.^8^ A predominance of Karp-like strains (64.7%) was also reported in five north Indian states.^9^ Using a type-specific antigen of a 56 kDa gene by nested PCR and phylogenetic analysis from 22 cases, strains with a predominance of clustering (57%) with Gilliam-type have been reported in Karnataka.^10^ In Karnataka, Andhra Pradesh and Kerala, JG-v-like (48.97%), Karp-like (26.53%), JG-like (22.44%) and Kato-like (2.04%) strains have been identified.^11^ It is postulated that some strains produce eschars less commonly than other antigenic types.^8 12 13^

There are over 700 species of Trombiculid mites,^14^ with infectious larval stage mites (called ‘chiggers’)^15^ of the Leptotrombidium genus responsible for scrub typhus transmission (online supplemental figure 1). These mites are rarely submitted to diagnostic laboratories as they drop off the host before moulting to the nymphal stage. When larvae feed, a large amount of *O. tsutsugamushi* that colonises the salivary glands of mites is injected into their host.^16^ The bacteria remain an endosymbiont within the vector by transovarial and transmedial transmission. *O. tsutsugamushi* repeatedly escapes from membrane compartments such as phagosomes and cytoplasm in host cells throughout its life cycle.^17^ Following skin inoculation, *O. tsutsugamushi* progressively spreads to hepatocytes, cardiomyocytes and endothelial cells.^18^ Using a non-lytic budding mechanism, the organism exits cells. *Leptotrobidium deliense* and *Leptotrobidium akamushi* are the primary vectors for scrub typhus, with *Schoengastiella ligula* now considered a secondary vector, particularly in India.^19 20^ The antigenic variability and short-lived immunity to *Orientia* contribute to initial infections and reinfections.

### Preferred diagnostic and treatment strategies for scrub typhus

Serological tests and molecular assays are primary laboratory procedures for diagnosing scrub typhus. As per the Centre for Disease Control and Prevention, indirect immunofluorescence assay (IFA) is the reference standard, though it is rarely available in developing nations where prevalence is high.^21^ Immunochromatographic tests (ICTs) using either pooled cell lysates from various strains of *O. tsutsugamushi* or recombinant p56 or other outer membrane proteins as the antigen provide better sensitivity and specificity and may eventually replace the IFA. ICTs detect IgG, IgM and IgA antibodies against *O. tsutsugamushi* with moderate sensitivity (~70%). Sensitivity increases with fever duration but has a substantial number of false negative results. PCRs usually target the genes of outer membrane proteins. They may be more sensitive than serological tests detecting *Orientia* DNA in blood even during persistent phases of infection with no obvious clinical symptoms. While providing a cheap option for identifying rickettsial infections in resource-poor settings, the Weil-Felix agglutination test has poor sensitivity (~15%) and specificity (~96%) and is thus not preferred.^22^

Historically, scrub typhus has been treated with chloramphenicol. However, its use in pregnant women and infants is fraught with complications, due to which it is avoided in pregnancy and not prescribed to neonates. At the same time, tetracycline is contraindicated during pregnancy due to teratogenicity, and it can discolour children’s teeth. Doxycycline (a tetracycline derivative) was initially contraindicated in children but is now deemed reasonably safe in children under eight, particularly in life-threatening cases.^23 24^ The macrolide azithromycin is effective in penetrating polymorphonuclear leucocytes and macrophages, the target cells of *O. tsutsugamushi* and is also deemed safe for use in pregnant women and young children.^25 26^

## Methods

### Search strategy and selection criteria

The study protocol was registered on PROSPERO prior to conducting the study selection and data extraction process (PROSPERO registration no. CRD42024611771). To identify relevant literature on scrub typhus, a systematic search of three databases–PubMed, Scopus and Embase was conducted using PRISMA protocol with the search terms “*Orientia tsutsugamushi* in India”, “Rickettsia in India” AND “Scrub typhus in India”. The search was restricted to articles published in English between 2003 and 2023, excluding simple reviews. Articles with location-specific data on testing and positivity rates were included to analyse scrub typhus prevalence. At the same time, those describing clinical manifestations were used to assess the prevalence of different symptoms and complications in scrub typhus patients. To distinguish between seroprevalence and actual disease prevalence, a case of scrub typhus was defined as a positive for the *O. tsutsugamushi* pathogen using standardised tests (immuno-chromatography, Weil-Felix, IgM ELISA or indirect IFA) in addition to suspicion or clinical diagnosis of scrub typhus and/or related signs. A descriptive review was applied to case reports to describe diagnostic accuracy and specific treatment outcomes.

### Data extraction

Two independent reviewers (RC and SSAH) screened all identified citations and extracted the relevant data. Titles and abstracts were screened using Covidence software, which uses Cochrane’s Risk of Bias 2 tool.^27^ General information regarding study title, authors, citation, location and year of study; and details of the methodology, that is, the study type, population, gender, time-period, total number of patients tested, total number of positive patients, total number of patients included in the study, mean age, number of patients that survived or died and outcomes studied were extracted for all the articles on a predesigned template. For studies on clinical manifestations, a detailed list of all symptoms, complications and coinfections reported, as well as their frequency and outcomes, were extracted in a separate template. The final agreement on study eligibility and the data extracted was resolved after a discussion with a third reviewer (HS). The extracted data have been published as a new dataset and uploaded to the Mendeley Data repository.^28^

### Study quality assessment

Study quality was assessed using the National Heart, Lung and Blood Institute (NHLBI)^28^ study quality assessment tool. Selected studies were categorised based on study type, and the risk of bias was assessed based on questionnaires for each study type in the NHLBI tool. Two independent reviewers (RC and SSAH) assessed each question in the tool. For each response that was not a ‘yes’, the potential risk of bias in the study resulting from the flaw was assessed. Based on the responses, the studies were rated as poor, fair and reasonable. Finally, Cohen’s kappa value was calculated to estimate the degree of agreement between the two reviewers.

### Statistical analysis and data representation

Primary outcomes assessed were the state-wise prevalence, state-wise case fatality rate (CFR) and the prevalence of different clinical manifestations (symptoms and complications). Secondary outcomes, such as the choice of diagnostics and treatment methods and the prevalence of coinfections with scrub typhus were also assessed. A descriptive analysis of the presentation of scrub typhus in pregnancy and children, as well as peculiar clinical manifestations, was provided. Publications reporting specific outbreaks were plotted on maps year-wise. Diagnostics and treatments used were pooled using simple proportions. For articles that did not specify the year of study, the year of publication was considered.

Scrub typhus cases reported in different studies were aggregated district wise, and the cases were visualised on a map. Studies with overlapping inpatient data, determined by comparing the location, hospital name, study periods and the study population used in the study, were removed to prevent duplication. For districts in which data were available for more than 2 years, a bar plot was generated to show the time-wise variation in scrub typhus incidence. The studies documenting cases from the same time frame, within the same hospital and involving the same patient groups were clubbed together and duplicate entries were eliminated. A closer look at the district-wise data revealed that most of the scrub typhus cases were reported from large hospitals in major cities of a state, and the actual origin of the infection was not reported. Therefore, it was decided to conduct the analysis state-wise to prevent inaccurate reporting of the district in which the case occurred.

Data on the total number of observations and the total number of events were recorded on separate Excel sheets for each type of outcome, including only the studies for which that outcome was available. A detailed summary of the type of analysis for each study is available in the online supplemental table. State-wise prevalence and CFRs of scrub typhus were pooled using a random effects model and reported using proportions with a 95% CI. Symptoms and complications related to scrub typhus were grouped based on the affected target sites, and their pooled prevalence was estimated similarly using a random effects model. A random effects model assumes that accurate effect sizes vary across studies, thereby accounting for both within-study and between-study variability. This is more suitable when substantial between-study variability exists, in contrast to a fixed effects model that only accounts for sampling error. Pooled effect sizes were represented as forest and caterpillar plots. Pooled prevalence and CFRs were plotted on a map using QGIS V.3.4. Heterogeneity in study effect sizes was assessed using the I^2^ and τ^2^ statistics, which estimate the percentage of variability and the absolute variance in effect sizes of different studies, respectively. Heterogeneity based on I^2^ statistics was categorised as low (<25%), moderate (25%–75%) and high (>75%) and sensitivity was assessed in more detail using GOSH (graphical display of study heterogeneity) plots. GOSH plots represent all possible pooled effect size estimates from subsets of included studies, thereby providing insights into how individual studies affect the overall result and identifying clusters that may bias the pooled effect size. The GOSH plots were created using 10 000 subsets due to computational constraints as the number of studies were relatively high. Publication bias was assessed using funnel plots. All analyses and plots were performed in R^29^ using the packages meta^30^ and metafor.^31^

## Results

### Search results

**Figure 1:**
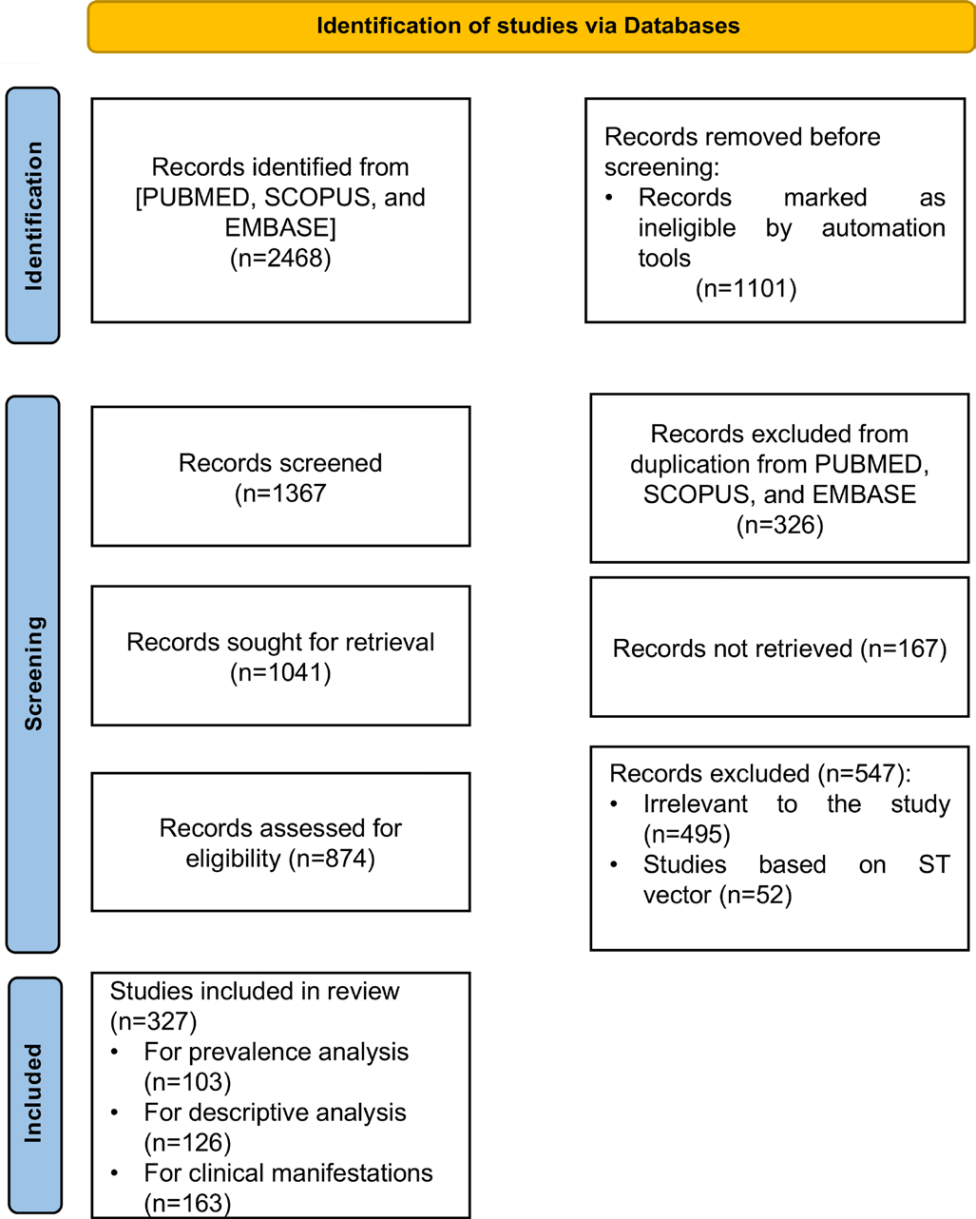


State-wise, the prevalence of scrub typhus was estimated from 103 studies with data on the total number of febrile patients and scrub typhus positivity rates. Details of clinical manifestations were available in 163 studies, including 65, which also had prevalence data, and 98, which included data on clinical manifestations only (figure 1). Almost all studies used hospital-based passive surveillance, and only four studies were conducted in the community using justification for sample size. While this may introduce selection bias, the results are still believed to be meaningful for identifying the distribution and prevalence of scrub typhus in India, as there is a significant lack of surveillance for scrub typhus.

### Risk of bias in studies

Study quality was assessed separately for cross-sectional, case-control and case report/series studies using the NHLBI Study quality assessment tool for that specific study type. The majority of the studies were rated as fair, with only a few studies scoring well on all questions (online supplemental table). A small fraction of the studies were also rated as poor and were set aside for descriptive analysis only. Cohen’s kappa estimate for the agreement between the two raters on the study quality of the three types of studies was found to be 0.62, 0.5 and 0.87, respectively. Therefore, there was strong agreement between the two raters on the quality of case reports and case series, whereas there was moderate agreement on the quality of cross-sectional and case-control studies. Almost all cross-sectional and most case-control studies did not apply any methods for sample size justification, which is a standard limitation that may affect the external validity of the studies. Furthermore, only a few studies were controlled for confounding factors in their analysis.

### Epidemiology of scrub typhus in India (2003–2023)

**Figure 2:**
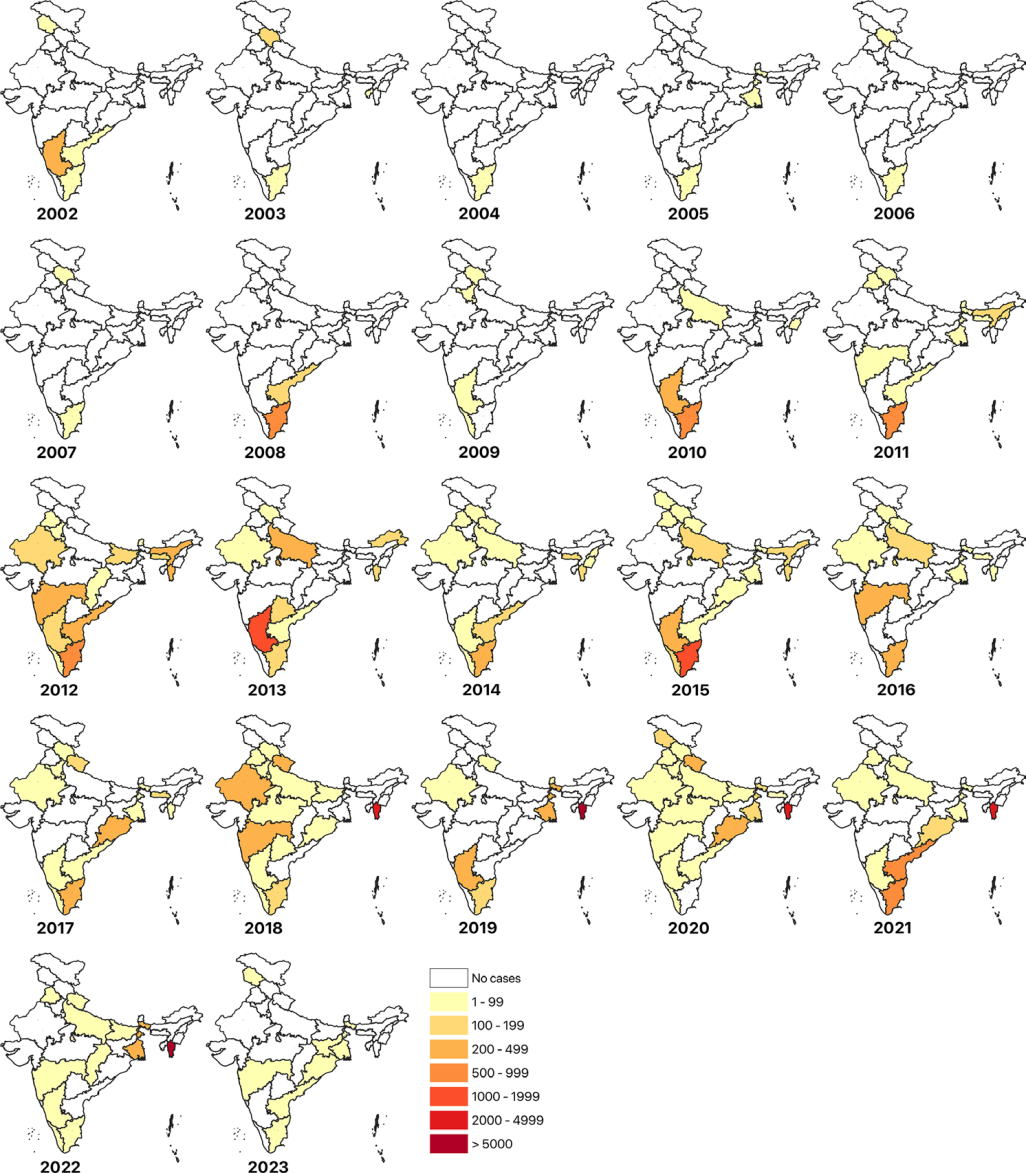
State-wise distribution of scrub typhus cases from 2002 to 2023 in India.

The number of scrub typhus publications has increased gradually over the years (online supplemental figure 2). From 2003 to 2023, 47 650 cumulative scrub typhus cases have been reported; four states/union territories, namely Mizoram, Tamil Nadu, Karnataka and Puducherry, have reported more than 2000 cumulative cases with a vast majority of the cases reported in 2019 (6532 cases) and 2022 (7110 cases). Reports of scrub typhus cases started to emerge from other parts of the country in 2010, and most states have reported scrub typhus since then (figure 2, online supplemental figure 3). Mizoram was most severely affected, with 19 651 cases during 2018–2022^32^ compared with 907 cases between 2012 and 2017 (figure 2, online supplemental figure 3).^33^ The most affected districts in Mizoram were Aizawl, Champai, Serchhip, Lungtai and Lawangtai (online supplemental figure 4). Tamil Nadu consecutively reported a high incidence of scrub typhus from 2010 to 2017, peaking in 2010, 2011 and 2015, with more than 900 cumulative cases, subsequently dropping below 300 in recent years (figure 2, online supplemental figure 3). Differences between genders were not significant, with the proportion of males affected (~47%) slightly higher than females (43%).

**Figure 3:**
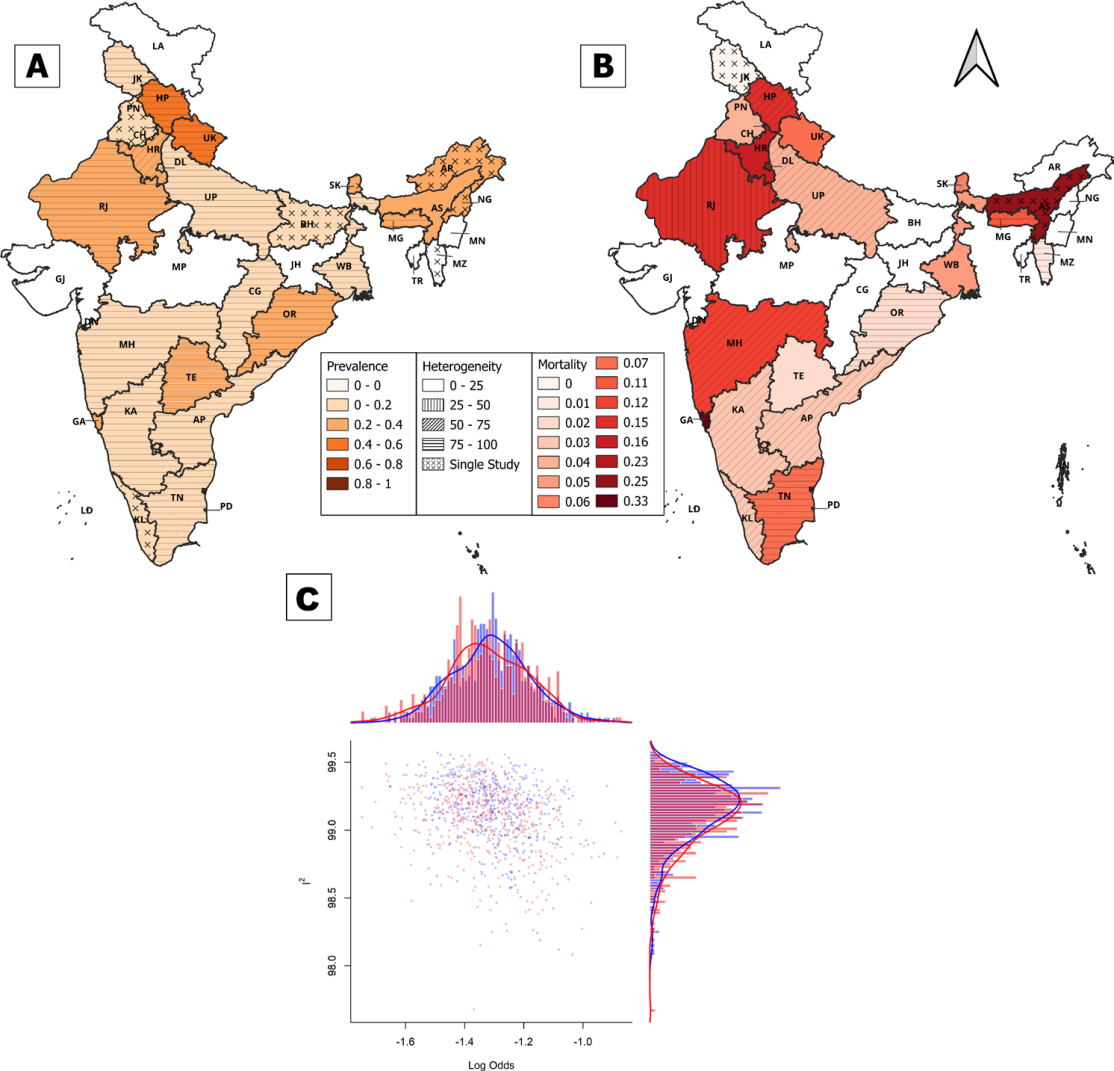
Pooled (**A**) prevalence and (**B**) case fatality rate of scrub typhus in different states of India from published literature between 2003 and 2023 using a random effects model. (**C**) Heterogeneity in prevalence studies of scrub typhus.

Even though Mizoram had the highest number of scrub typhus cases, the prevalence of scrub typhus in Mizoram could not be estimated as data on the total number of tests is unavailable. Furthermore, in Arunachal Pradesh, Bihar, Goa, Kerala, Nagaland, Punjab and Sikkim, prevalence data were available in only a single study, due to which pooled prevalence could not be estimated. Nevertheless, our results highlight the widespread distribution of scrub typhus in India (online supplemental material forest plot 1). District-wise data on scrub typhus cases showed strong bias towards districts in which the capital city of the state exists. A likely cause of this is that rural populations often travel to major cities for seeking better treatment, particularly in less well-known infections such as scrub typhus. Therefore, the district-wise prevalence likely underestimates the overall prevalence of the infection, and therefore, the meta-analysis was conducted state-wise instead. Among other states with more than one study, the degree of heterogeneity is significantly large, with Delhi and Assam being the only exceptions (0%–45% heterogeneity) with a pooled prevalence rate of 10% and 21%, respectively (figure 3A). The significant heterogeneity in pooled effect sizes of other states is likely a result of differences in study periods and the specific study sites within the state. Among these states, the highest prevalence of scrub typhus is estimated to be in Himachal Pradesh and Uttarakhand (44% and 53%, respectively), followed by Odisha, Rajasthan and Haryana (23%–32%) (online supplemental material forest plot 1). Further, a roughly homogeneous and symmetrical distribution indicated that there were no distinct population clusters with a different effect size and values were more or less uniformly distributed across the different studies (figure 3C). Publication bias in studies used for assessing prevalence was visible but not very significant (online supplemental figure 5).

### Case fatality rates

We noted 1088 fatalities reported from all states over two decades, with a pooled CFR of 5% out of 35 243 cumulative cases that had mortality data available, based on a random effects model (figure 3B). The CFR of scrub typhus was highest in Assam (25%) and Goa (33%) (figure 3B). However, these were based on results from only a single study and may not represent the actual CFR in these states (online supplemental material forest plot 2). Among the states with more than two studies with mortality data, pooled CFRs were highest in Delhi (23%), Haryana (14%), Himachal Pradesh (10%), Rajasthan (10%), Maharashtra (12%) and Meghalaya (7%) and significantly lower for the remaining states (<5%). Even though Mizoram had the highest number of cumulative cases of scrub typhus over the years, it had a pooled fatality rate of only 1% based on the random effects model (figure 3B). Notably, most fatalities occurred either due to acute respiratory distress syndrome (ARDS) or multiorgan dysfunction syndrome.^34^ Studies reporting mortality were fewer in number, and there was some bias in the smaller sample size studies towards the left (online supplemental figure 5).

### Outbreaks of scrub typhus

**Figure 4:**
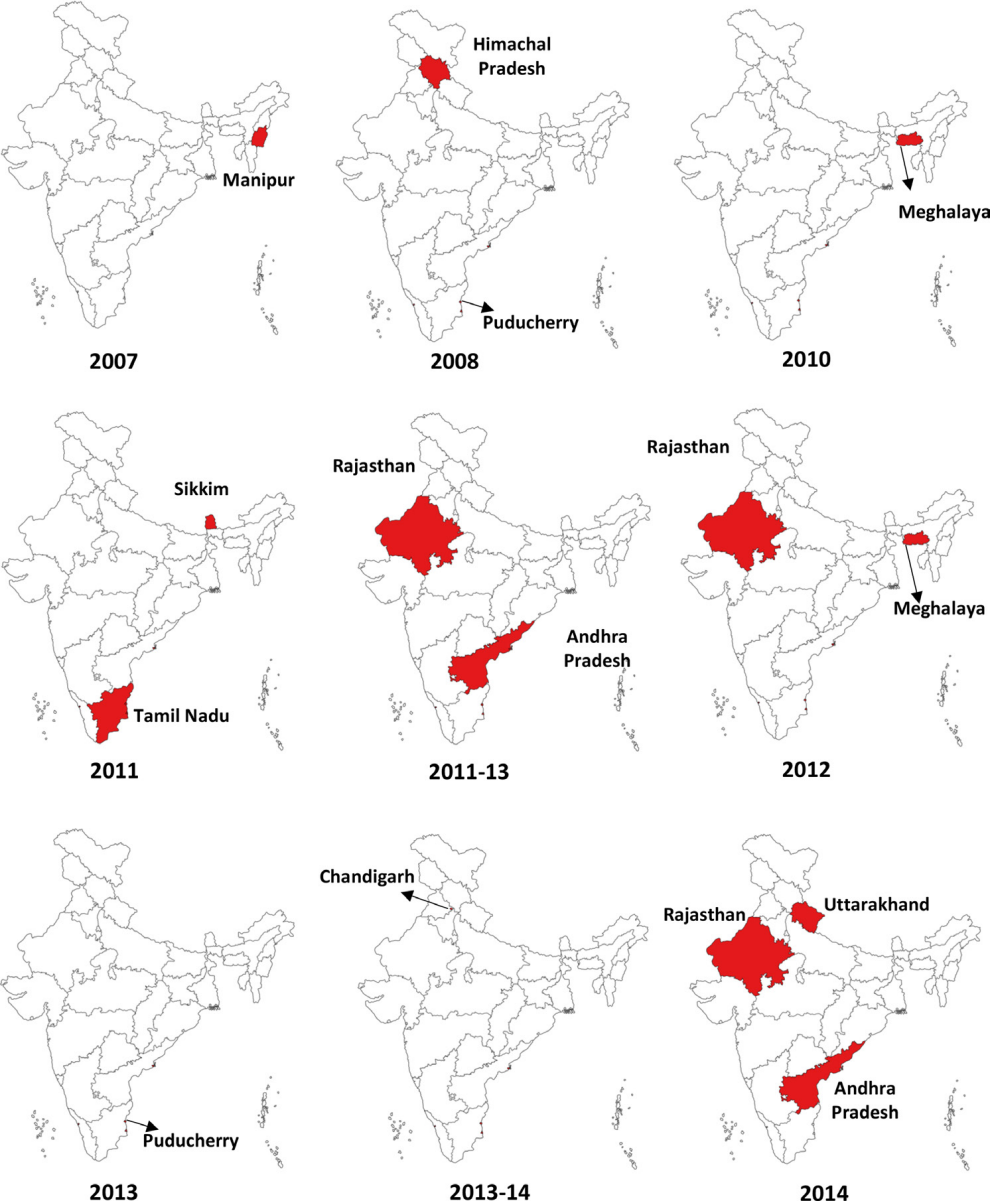
Outbreaks of scrub typhus reported in published literature over the past two decades (2003–2023).

Besides episodic cluster cases of scrub typhus, several outbreaks with high CFRs have occurred regularly throughout India (figure 4). Two outbreaks in Himachal Pradesh with a cumulative 158 cases and 34 deaths were reported in 2003.^35 36^ Another 50 cases followed this in Puducherry during 2006–2008.^37^ Since 2001, the northeast state of Manipur has experienced regular post-monsoon outbreaks of febrile illness. In 2007, an outbreak in Manipur occurred, reporting 38 patients with two fatalities (5.3%).^38^ Within 2 years, an outbreak in an adjoining northeastern state, Meghalaya, in 2009–2010 affected 24 children.^39^ Apart from these, in consecutive years, four outbreaks were also reported from four different states having relatively different geographical locations and climates: Tamil Nadu (52 cases),^40^ Andhra Pradesh (174 cases),^41^ Sikkim (63 cases)^42^ and Rajasthan (42 cases)^43^ (figure 4). Two outbreaks occurred in 5 years in two different cities in Rajasthan; 66 patients were positive in Kota in 2014,^44^ whereas 1340 positive cases were from Udaipur in 2019.^45^ Further, a recent report illustrated a high number of cases from Mizoram over 5 years since 2018, with the highest being reported to be 6542 cases in 2022, followed by 5859 cases in 2019, depicting Mizoram as the most affected state in India.

### Diagnosis and therapeutic regime of scrub typhus

**Figure 5:**
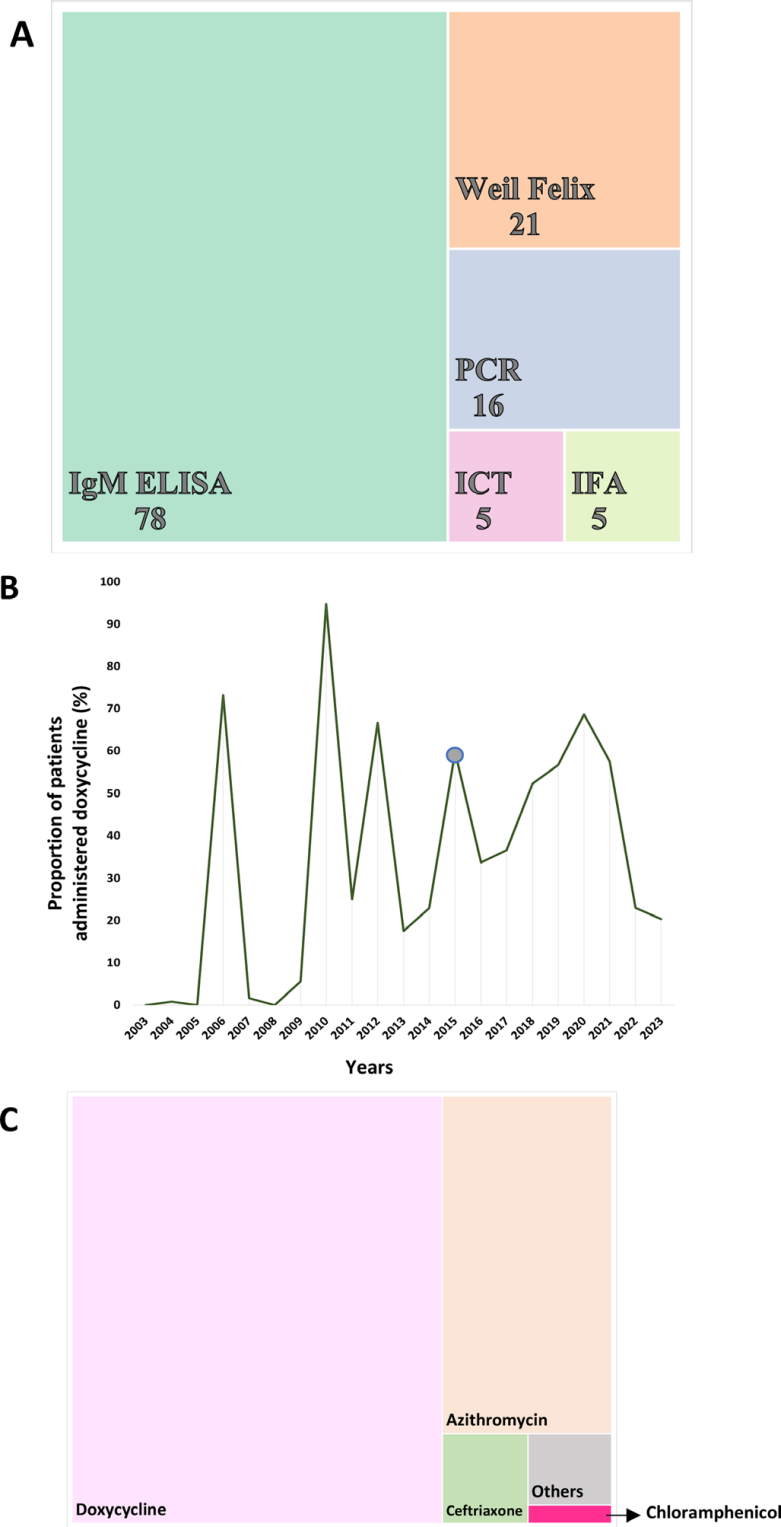
(A) Number of studies that used different types of diagnostic tests for scrub typhus and (B) choice of therapeutic regime for scrub typhus (%) in studies in India. (C) Percentage of patients administered doxycycline in India since 2003. The blue dot on the graph indicates the time when DHR-ICMR implemented the use of doxycycline as the drug of choice for scrub typhus treatment in the country. DHR-ICMR, Department of Health Research-Indian Council of Medical Research; ICT, immunochromatographic test; IFA, immunofluorescence assay.

The primary choice of diagnosis in the reviewed studies was the Weil-Felix test, based on ~61% of the cases (25 091 cases, including a mass survey of 19 651 cases from Mizoram).^32^ In comparison, the serological methods IgG and IgM were used for ~50% of cases (20 540). PCR was the third diagnostic test of choice (4.6%), while ~5% of cases were diagnosed using a combination of techniques (ELISA+Weil-Felix and ELISA+PCR). Up to 78 studies used IgM ELISA for diagnosis and 21 studies using Weil-Felix tests for diagnosing scrub typhus in India (figure 5A).

In 2015, the Department of Health Research-Indian Council of Medical Research specified doxycycline as the first-line treatment for suspected Rickettsial diseases before confirmation (figure 5B).^25^ By 2020, ~70% of patients were treated with it (figure 5B). Doxycycline was the choice of drug (n=17 362; ~38%), followed by azithromycin (13%) (figure 5C). A recent study demonstrated that in both modified intention-to-treat and per-protocol populations, combination therapy involving intravenous doxycycline and azithromycin was superior to monotherapy with either drug about the primary composite outcome of persistent fever and death at day 28.^46^ Other studies did not find any variation in the primary result between the monotherapy groups.^25^

### Diverse clinical spectrums of scrub typhus

Scrub typhus symptoms coincide with those of several other infectious diseases. A significant variation in heterogeneity in studies on general, gastrointestinal, pulmonary and inflammatory symptoms compared with studies on cardiac, hepatic, neurological and other symptoms was observed in the current study (online supplemental figure 6). Similarly, heterogeneity was higher in studies with pulmonary and neurological complications (online supplemental figure 7). The forest plots related to symptoms and complications are provided in online supplemental material forest plot 3–6. Funnel plots indicate that publication bias in the studies for each symptom subgroup existed but was relatively low (online supplemental figure 8). However, publication bias was more visible in complications, particularly for cardiac, pulmonary and neurological complications (online supplemental figure 9).

**Figure 6:**
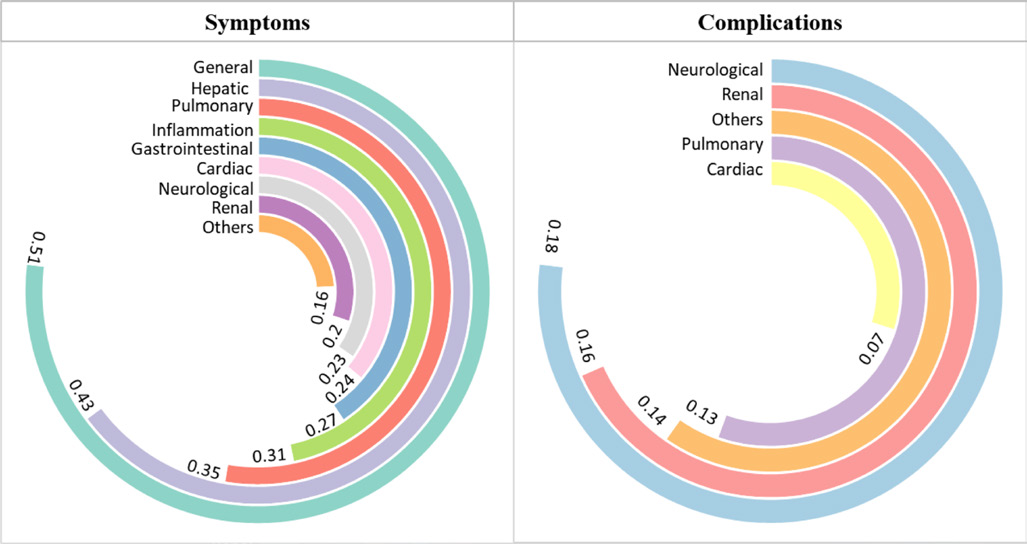
Pooled prevalence of clinical manifestations (symptoms and complications) using a random effects model of scrub typhus in the Indian population. Symptoms and complications included in the categories above have been defined in online supplemental table 2.

Scrub typhus manifests as AUFI after an incubation period of 6–21 days with an overall pooled prevalence of 97% (online supplemental material forest plot 3). Other general symptoms include headaches, chills and rigour, myalgia and arthralgia, malaise and pallor with moderate pooled prevalence (33%–56%) (figure 6; online supplemental material forest plot 3). Eschar, a notable feature of scrub typhus, had a relatively lower pooled prevalence of only 26%, likely as they often occur in body parts that are not easily visible, like genitals. *O. tsutsugamushi* parasite invades the hepatocyte cells, leading to hepatic symptoms like hepatomegaly and hepatic dysfunction, with an overall pooled prevalence rate of 44% (figure 6). Hepatomegaly was the most common liver-related symptom (46%) (online supplemental material forest plot 7). Pulmonary symptoms (35%) include cough and sore throat (34%), breathing problems like tachypnoea and dyspnoea (37%), crepitations (26%) and haemoptysis (online supplemental material forest plot 9). Severe involvement of the lungs includes bronchitis and interstitial pneumonitis (11%), respiratory dysfunction (49%) and ARDS (13%). Clinical features such as dyspnoea, cough, low blood pressure, mean arterial pressure and hypovolaemia were significant in scrub typhus patients with ARDS. A relatively significant incidence of ARDS (prevalence rate-13%) was observed (online supplemental material forest plot 10). Inflammation (31%) in scrub typhus affects the spleen (34%), lymph nodes (33%), meninges (24%) and pancreas (6%) and may also lead to fluid buildup (28%) and/or facial puffiness (34%) (online supplemental material forest plot 8). Gastrointestinal symptoms noted were nausea (43%), diarrhoea (11%), vomiting (35%), abdominal pain (26%), jaundice/icterus (18%), gastrointestinal bleeding (15%) and other gastric abnormalities (43%) (online supplemental material forest plot 4), with overall pooled prevalence lower than that of general symptoms (26%) (figure 6). Cardiac-associated symptoms (24%) included hypotension (22%) and tachycardia (65%) only, with cardiac complications such as myocarditis (4%), cardiac dysfunction (16%) and disseminated intravascular coagulation (7%) also reported in some studies (online supplemental material forest plot 5 and 6) (figure 6).

Neurological signs in scrub typhus are comparable to those of other rickettsial disorders, with an overall pooled prevalence of 23%, leading to severe complications in 18% of people (figure 6). Central nervous system (CNS) involvement in scrub typhus includes impaired consciousness (28%), seizures/coma (18%) and nerve palsy (1%) (online supplemental material forest plot 13). Acute encephalitis syndrome (AES) is a common severe presentation of scrub typhus, observed in 41% of scrub typhus patients in the meta-analysis (online supplemental material forest plot 14), with other complications such as meningitis (33%), meningoencephalitis (15%), shock (19%) and AES (41%) also reported fairly frequently (online supplemental material forest plot 14). Other severe complications of scrub typhus can include intracerebral or subarachnoid haemorrhage, postinfective demyelination syndromes, ataxia, transverse myelitis, plexopathy, neuroleptic malignant syndrome, cerebellitis, radiculoneuropathy, transient parkinsonism and cerebral venous thrombosis, with rare neurological complications such as Guillain-Barre syndrome and opsoclonus-myoclonus syndrome (OMS) also observed in 1.52% and 9.52% of the cases, respectively, as per the descriptive analysis of case series studies. Renal involvement in scrub typhus had a pooled prevalence of ~20% (figure 5A), ranging from mild urinary abnormalities such as oliguria/dysuria/anuria (18%) and proteinuria (40%) to severe complications such as acute kidney injury (16%) (online supplemental material forest plot 11 and 12). Less common symptoms of scrub typhus are abnormal bleeding (9%), anaemia (19%), pleural effusion (13%) and conjunctival congestion (21%) (online supplemental material forest plot 15 and 16). Scrub typhus affects highly vascularised organs such as the liver, kidney, brain and lungs, resulting in multiorgan failure (18% prevalence). About 13% of the scrub typhus patients suffered from various co-infections and comorbidities such as hepatitis (25%), diabetes (9%), leptospirosis (14%), malaria (6%) and dengue (9%) (online supplemental material forest plot 17).

### Scrub typhus in pregnancy and children: the vulnerable groups

Since 2003, 15 studies from India reported complications during pregnancy due to scrub typhus in 142 pregnant females, with mortality in five females (3.5%) due to the presentation of advanced disease. Approximately 40% of pregnancies led to poor fetal outcomes: abortions (54%), miscarriages (3.5%), stillbirths (12%), preterm birth (28%) and infant deaths (1.75%). Four studies from Tamil Nadu reported 83 confirmed cases in pregnant females,^47^,^50^ with the most common symptoms reported being fever (48%), neurological manifestations (13%) and eschar (27%). Most cases were treated with azithromycin (60%), followed by doxycycline (13%).^47^,^50^

Scrub typhus among neonates is rare but should be considered a differential diagnosis for neonates presenting with fever, hepatosplenomegaly, thrombocytopaenia and elevated C-reactive protein (CRP), especially in endemic regions.^51 52^ Transmission mechanisms include transplacental transfer, perinatal bloodborne transmission and miteborne transmission. Studies in Uttar Pradesh reported *O. tsutsugamushi* positivity among children 2–15 years of age.^53^ Studies have reported ~30 cases of neonatal scrub typhus, and transmission mechanisms include transplacental transfer, perinatal bloodborne transmission and miteborne transmission. Unfavourable fetal and neonatal outcomes can be decreased by preventing and treating scrub typhus during pregnancy.

## Discussion

Scrub typhus is increasingly recognised as a public health issue in India, yet its extent remains underexplored. This analysis of its spatiotemporal spread and mortality rates over two decades provides critical insights. The disease has notably emerged in Mizoram since 2012, with as many as 20 558 cases reported during a period of 10 years (2012–2022), as per the state Integrated Disease Surveillance Programme (IDSP).^32 33^ These cases were based on the scrub-typhus zero surveillance test^33^ and on Tsutsugamushi or Weil-Felix OXK positivity, representing one of the most comprehensive datasets in the selected studies.^32^ This surge may be linked to ecological changes like bamboo flowering, which boost mite populations.^32 33^ Some of the Indian northeastern states (Assam, Arunachal Pradesh, Meghalaya and Nagaland) also show high prevalence (21%–40%), possibly due to agricultural and forest-dependent lifestyles, which increase exposure to vector habitats.^54^ Endemicity is also significant in regions like the northwestern Himalayas (Himachal Pradesh-44%, Uttarakhand-53%, Jammu & Kashmir-34%) and Rajasthan-32%, which are characterised by suitable vegetation and farming practices.^55^ Moderate prevalence in Odisha-30%, Haryana-23% and Uttar Pradesh-16% links scrub typhus to living conditions such as firewood storage and proximity to livestock. Southern states/territories like Karnataka, Puducherry and Telangana show moderate prevalence, while Tamil Nadu has relatively lower rates (15%) despite extensive surveillance. By 2020, scrub typhus was reported in 20 of 36 states, with improved detection during COVID-19 surveillance highlighting its widespread presence. The IDSP unit in India analysed data from 2015 to 2019 to suggest that Alwar district (Rajasthan) continuously reported scrub typhus with a cumulative 4904 cases for 2 years (2018–2019).^56^ The state reporting system may not have reported cases to the Central Surveillance Unit, IDSP.^56^ Hence, there is a possibility that scrub typhus cases are not being updated for central Indian surveillance, and this will need synergy.

State-wise, CFR ranged from 1% to 33%, and this is significantly higher than for other vector-borne diseases. Delhi recorded an alarming pooled CFR of 23%, though this was based on limited data. Scrub typhus has also been implicated in AES, notably in Uttar Pradesh and Assam, thus complicating clinical management.^53 57^ Genetic diversity among serotypes and diagnostic challenges exacerbate underdiagnosis. Weil-Felix test, though less sensitive, is widely used in resource-constrained settings like in Mizoram, where government-tailored testing improved detection.^32^ Integrating multiple diagnostic methods and prioritising genotypic studies is crucial for developing point-of-care tools. Efforts to estimate cumulative deaths rather than relying solely on CFRs reveal Tamil Nadu with the highest number of deaths (222), followed by Punjab and Himachal Pradesh.

Diverse clinical symptoms for scrub typhus are also a significant cause of concern. We found that the highest pooled prevalence was of general symptoms at ~51%, followed by hepatic-43%, pulmonary-35% and inflammatory symptoms at 31% (figure 5A). At the same time, neurological complications arising due to scrub typhus were the highest (18%). In pulmonary complications (13%), ARDS was the most common complication arising due to scrub typhus, which is often correlated with fatalities. Doxycycline remains the primary treatment for scrub typhus. Combination therapy with doxycycline and azithromycin is proposed for severe cases, offering a potentially more robust approach to treatment.^46^

A key limitation of the results of our study is the high heterogeneity and publication bias in the studies included in the meta-analysis. This is unavoidable, as studies differ significantly in the surveillance season, exact geographical location and the overall period of the analysis. Nevertheless, the results from the study provide an important understanding of the widespread distribution and the gradual expansion of scrub typhus across India, as well as the diverse clinical spectrums. The diverse epidemiology of scrub typhus across India underscores the need for targeted interventions, improved diagnostic capacity, and strategic treatment approaches to mitigate its impact on public health.

## Conclusions

Based on current data, *O. tsutsugamushi* infections exhibit distribution with no apparent restrictions by climatic conditions, socioeconomic status, gender or age in India. The infection is emerging as a life-threatening disease. Effective control and prevention strategies necessitate early diagnostic procedures in endemic areas with high caseloads. Establishing a robust surveillance system nationwide is crucial. Initiatives like distributing diagnostic kits, as seen in Mizoram since 2019, can reflect the actual disease burden and facilitate appropriate preventive measures. Notably, CFRs are closely linked to scrub typhus-induced complications, emphasising the importance of early detection and treatment in averting fatalities.

## Supplementary material

10.1136/bmjgh-2025-018998 online supplemental file 1

## Data Availability

All data relevant to the study are included in the article or uploaded as supplementary information.
