# Supplementary material for: Spatiotemporal epidemiology and clinical manifestations of two decades of scrub typhus in India: a systematic review and meta-analysis
Source: BMJ Glob Health. 2025 Aug 3;10(8):e018998. doi: 10.1136/bmjgh-2025-018998 (PMC12320085; doi:10.1136/bmjgh-2025-018998)
Supplement: online supplemental file 1 [file bmjgh-10-8-s001.pdf]

Spatiotemporal epidemiology and clinical manifestations of two decades of Scrub Typhus in India: A systematic review and meta-analysis

Rini Chaturvedi[1], Syed Shah Areeb Hussain[2], S. Hayavadhan[3], Bijay R. Mirdha[1], Amit Sharma[2]

Supplementary Materials

Table of Contents

Supplementary Information 3

Detailed Explanation of random effects model used to pool data 4

Sources and Measures of Heterogeneity 5

Risk of Bias 5

Supplementary Table 7

Supplementary Table 1: Risk of bias of individual studies based on two raters 7

Supplementary Figures 13

Supplementary figure 1. Total number of publications for scrub typhus over the years. Y-axis represents total number of publications 13

Supplementary Figure 2. Cumulative cases of scrub typhus in Indian states year wise. Other states which reported scrub typhus cases were Madhya Pradesh, Jharkhand, and Tripura. X-axis represents total number of cases for corresponding years. *The study year when the study was completed was considered. 14

Supplementary Figure 3. Caterpillar plots representing the distribution of values and pooled log odds of different categories of symptoms in scrub typhus 15

Supplementary Figure 4. Caterpillar plots representing the distribution of values and pooled log odds of different categories of complications in scrub typhus 16

Supplementary Figure 5. District wise distribution of cumulative cases of scrub typhus as well as temporal trends in scrub typhus cases in districts where year-wise data was provided or in which more than one study was conducted at different time periods.... 17

Supplementary Figure 6. Number of studies that used different types of diagnostic tests for Scrub Typhus 18

Forest Plot 1. Pooled prevalence of scrub typhus in different states of India using a random effects model 25

Forest plot 2. Pooled estimates of case fatality ratio of scrub typhus cases in different states of India using a random effects model 28

Forest plot 3. Pooled prevalence of different types of general symptoms of scrub typhus 38

Forest plot 4. Pooled prevalence of different types of gastrointestinal symptoms of scrub typhus 43

Forest plot 5. Pooled prevalence of different types of cardiac symptoms of scrub typhus 44

Forest plot 6. Pooled prevalence of different types of cardiac complications of scrub typhus 45

Forest plot 7. Pooled prevalence of different types of hepatic symptoms of scrub typhus 47

Forest plot 8. Pooled prevalence of different types of inflammation symptoms of scrub typhus 51

Forest plot 9. Pooled prevalence of different types of pulmonary symptoms of scrub typhus........54
Forest plot 10. Pooled prevalence of different types of pulmonary complications of scrub typhus........56
Forest plot 11. Pooled prevalence of different types of renal symptoms of scrub typhus........57
Forest plot 12. Pooled prevalence of different types of renal complications of scrub typhus........58
Forest plot 13. Pooled prevalence of different types of neurological symptoms of scrub typhus........61
Forest plot 14. Pooled prevalence of different types of neurological complications of scrub typhus........63
Forest plot 15. Pooled prevalence of other symptoms of scrub typhus........64
Forest plot 16. Pooled prevalence of other complications of scrub typhus........65
Forest plot 17. Pooled prevalence of co-infections along with scrub typhus reported in literature........66

Detailed Explanation of random effects model used to pool data

Effect sizes observed in different studies can vary significantly amongst each other due to variations in the processes that generate the data. If these variations are not accounted for, it can result in a significant bias in the estimation of the pooled effect size. Therefore, in meta-analysis a statistical model that can account for this heterogeneity in effect sizes is used to pool the effect size from different studies. By specifying the statistical model, an approximate representation of the reality behind the data is provided. Even though a meta-analysis provides a single effect size estimate by combining the results from multiple studies, it is important to identify why and by how much the studies differ in their effect sizes.

A fixed-effects model assumes that effect sizes from all studies belong to a homogenous population and all variation in the observed effect size is a result of the sampling error. Therefore, a fixed effects model assumes that all studies are trying to estimate the same true effect size. This can be explained by the equation

$$\bar{\theta}_k = \theta + \varepsilon_k$$

where $\bar{\theta}_k$ is the observed effect size in the study k, θ is the true effect size of the population, and ε_k is the sampling error.

However, in most real-world examples, the assumptions of the fixed effects model may be an oversimplification, as the studies in a meta-analysis can never be perfectly homogenous. Besides the sampling error, there are many different types of errors that can result in differences in the observed effect sizes. Therefore, to account for this, a random effects model includes an additional source of error, due to which the true effect size is different for each study included in the meta-analysis. The actual effect size of any study is then changed as follows:

$$\overline{\theta_k} = \theta_k + \varepsilon_k$$

where θ_k is the true effect size one single study, k. The true effect size of study k, here, is part of an overarching distribution of true effect sizes that has a mean μ, and the true effect size of the study varies from this overarching distribution due to an error ζ_k. Therefore, the above formula is converted as follows:

$$\bar{\theta}_k = \theta_k + \mu + \zeta_k$$

Even with an extremely strict inclusion/exclusion criteria, it is generally highly unlikely that the studies included in a meta-analysis are homogenous.

In the present study, there are likely to be many sources of error in the studies that have been pooled together in addition to the sampling error. This includes errors due to geographical differences within each state, temporal differences, differences in population age, gender, comorbidities, etc., differences in definitions of different outcomes, measurement bias, as well as the study design. Keeping this in mind, a fixed effects model would be insufficient to

estimate the true effect size, and therefore a random effects model has been applied in our study to pool the individual effect sizes from different studies.

Sources and Measures of Heterogeneity

Multiple sources of heterogeneity have been identified in the present analysis, which include but are not limited to the study design, geographical region, the study time period, diagnostic methods, as well as the population in which the prevalence is assessed. Therefore, it is expected that a significant amount of heterogeneity in the effect sizes of the studies included in the analysis will be observed.

Two widely used statistical measures were used to assess the heterogeneity in the present study, namely the I^2 statistic, and the τ^2 statistic.

I^2 quantifies the percentage of variability in the effect size estimates due to the true heterogeneity rather than random chance and can be calculated as follows:

$$I^2 = \frac{Q - df}{Q} \times 100$$

where Q is the Cochrain's Q statistic and df is the degrees of freedom. I^2 statistic between 0-25% indicates low heterogeneity, 25-50% indicates moderate heterogeneity, 50-75% indicates substantial heterogeneity and greater than 75% indicates high heterogeneity.

τ^2 is the estimate of the variance of true effect sizes across studies in a random-effects model. Unlike I^2, which is a relative measure, τ^2 gives an absolute measure of heterogeneity in the effect size scale (e.g., prevalence). Larger τ^2 values indicate greater heterogeneity between studies. It is estimated using methods like DerSimonian and Laird or restricted maximum likelihood (REML).

Risk of Bias

Study quality was assessed separately for cross-sectional, case-control, and case report/series studies using the NHLBI Study quality assessment tool for that specific study type. Cohen's Kappa estimate for the agreement between the two raters on the study quality of the three types of studies was found to be 0.62, 0.5, and 0.87, respectively. Therefore, there was strong agreement between the two raters on the quality of case reports and case series, whereas there was moderate agreement on the quality of cross-sectional and case-control studies.

The majority of the studies were rated as fair, with only a few studies scoring well on all questions. A small fraction of the studies was also rated as poor and were set aside for descriptive analysis only. Almost all cross-sectional and most case-control studies did not apply

any methods for sample size justification, which is a common limitation that may affect the external validity of the studies. Furthermore, only a few studies controlled for confounding factors in their analysis.

Supplementary Table

Supplementary Table 1: Risk of bias of individual studies based on two raters

FID	Study ID	Population	Analysis	Rater 1	Rater 2
CR1	Attur et. al. (2013)	All Patients (Hospital)	Symptoms…	Good	Good
CR2	Behara et. al. (2021)	Children (Hospitals)	Symptoms…	Good	Good
CR3	Abbas et. al. (2021)	NA	Descriptive	Good	Good
CR4	Agarwal et. al. (2021)	NA	Descriptive	Good	Good
CR5	Aggarwal et. al. (2009)	NA	Descriptive	Good	Good
CR6	Aggarwal et. al. (2012)	NA	Descriptive	Fair	Good
CR7	Agrwal et. al. (2019)	NA	Descriptive	Good	Good
CR8	Ahmed et. al. (2014)	All Patients (Hospital)	Symptoms…	Good	Fair
CR9	Arasu et. al. (2021)	Pregnant Women	Descriptive	Good	Good
CR10	Arora et. al. (2022)	NA	Descriptive	Good	Good
CR11	Atam et. al. (2020)	NA	Descriptive	Good	Good
CR12	Avasthi et. al. (2018)	NA	Descriptive	Good	Good
CR13	Bansal et. al. (2022)	NA	Descriptive	Good	Good
CR14	Baruah et. al. (2016)	NA	Descriptive	Good	Good
CR15	Basheer et. al. (2015)	NA	Descriptive	Good	Good
CR16	Bhardwaj et. al. (2013)	NA	Descriptive	Good	Good
CR17	Bhargava et. al. (2019)	NA	Descriptive	Good	Fair
CR18	Bhat et. al. (2015)	NA	Descriptive	Fair	Fair
CR19	Bhatt et. al. (2014)	NA	Descriptive	Good	Good
CR20	Bolla et. al. (2023)	NA	Descriptive	Good	Good
CR21	Chandra et. al. (2023)	NA	Descriptive	Good	Good
CR22	Chandramohan et. al. (2015)	NA	Descriptive	Good	Good
CR23	Chandrashekhar et. al. (2014)	NA	Descriptive	Good	Good
CR24	Chaturvedi et. al. (2016)	NA	Descriptive	Good	Good
CR25	Chaudhry et. al. (2009)	NA	Descriptive	Good	Good
CR26	Chauhan et. al. (2021)	NA	Descriptive	Good	Good
CR27	Choudhary et. al. (2020)	NA	Descriptive	Good	Good
CR28	Chowdhary et. al. (2022)	NA	Descriptive	Good	Good
CR29	D'sa et. al. (2012)	NA	Descriptive	Good	Good
CR30	Das et. al. (2023)	NA	Descriptive	Good	Good
CR31	Dev et. al. (2019)	NA	Descriptive	Good	Fair
CR32	Devarajan et. al. (2012)	NA	Descriptive	Good	Good
CR33	Devi et. al. (2017)	NA	Descriptive	Good	Fair
CR34	Didel et. al. (2017)	NA	Descriptive	Good	Fair
CR35	Ete et. al. (2016)	NA	Descriptive	Good	Fair
CR36	Gangula et. al. (2017)	NA	Descriptive	Good	Fair
CR37	Garg et. al. (2022)	NA	Descriptive	Good	Good
CR38	Ghosh et. al. (2020)	NA	Descriptive	Good	Good
CR39	Ghosh et. al. (2022)	NA	Descriptive	Good	Good
CR40	Gopal et. al. (2010)	NA	Descriptive	Good	Good
CR41	Goswami et. al. (2013)	NA	Descriptive	Good	Good
CR42	Goyal et. al. (2019)	NA	Descriptive	Good	Good
CR43	Guleria et. al. (2018)	NA	Descriptive	Good	Good
CR44	Gupta et. al. (2012)	NA	Descriptive	Good	Good
CR45	Gupta et. al. (2018)	NA	Descriptive	Good	Good
CR46	Gupta et. al. (2021)	Pregnant Women	Descriptive	Good	Good
CR47	Gupta et. al. (2022a)	NA	Descriptive	Good	Good
CR48	Gupta et. al. (2022b)	NA	Descriptive	Good	Good
CR49	Handattu et. al. (2018)	NA	Descriptive	Good	Good
CR50	Hazra et. al. (2021)	NA	Descriptive	Good	Good
CR51	Iqbal et. al. (2011)	NA	Descriptive	Good	Good
CR52	Iqbal et. al. (2015)	NA	Descriptive	Good	Good
CR53	Ittyachen et. al. (2009)	NA	Descriptive	Good	Good
CR54	Ittyachen et. al. (2017)	NA	Descriptive	Good	Good
CR55	Jain et. al. (2014)	NA	Descriptive	Good	Good
CR56	Jain et. al. (2020)	Pregnant Women	Descriptive	Good	Good
CR57	Jain et. al. (2021)	NA	Descriptive	Good	Good
CR58	Jajoo et. al. (2017)	NA	Descriptive	Good	Good
CR59	James et. al. (2020)	NA	Descriptive	Good	Good
CR60	Jamil et. al. (2019)	All Patients (Hospital)	Symptoms…	Good	Good
CR61	Jatiya et. al. (2019)	NA	Descriptive	Good	Good
CR62	Jena et. al. (2014)	NA	Descriptive	Good	Good
CR63	Jessani et. al. (2016)	NA	Descriptive	Good	Good

CR64	Kalita et. al. (2021)	NA	Descriptive	Good	Good
CR65	Kamath et. al. (2023)	NA	Descriptive	Good	Good
CR66	Karanth et. al. (2013)	NA	Descriptive	Good	Good
CR67	Karim et. al. (2016)	NA	Descriptive	Good	Good
CR68	Kasinathan et. al. (2019)	NA	Descriptive	Good	Good
CR69	Khanna et. al. (2022)	NA	Descriptive	Good	Good
CR70	Koti et. al. (2015)	NA	Descriptive	Good	Good
CR71	Kumar et. al. (2013)	NA	Descriptive	Good	Good
CR72	Kumar et. al. (2020)	NA	Descriptive	Good	Good
CR73	Kundavaram et. al. (2014)	NA	Descriptive	Good	Good
CR74	Mahajan et. al. (2007)	NA	Descriptive	Good	Good
CR75	Mahajan et. al. (2009)	Pregnant Women	Descriptive	Good	Good
CR76	Mahajan et. al. (2011)	NA	Descriptive	Good	Good
CR77	Mahajan et. al. (2012)	NA	Descriptive	Good	Good
CR78	Mahajan et. al. (2014)	NA	Descriptive	Good	Good
CR79	Mahajan et. al. (2015)	NA	Descriptive	Good	Good
CR80	Mahajan et. al. (2016b)	NA	Descriptive	Good	Good
CR81	Majumder et. al. (2021)	NA	Descriptive	Good	Good
CR82	Manappallil et. al. (2021)	NA	Descriptive	Good	Good
CR83	Margaret et. al. (2023)	NA	Descriptive	Good	Good
CR84	Mathai et al. (2003b)	Pregnant Women (Hospital)	Descriptive	Good	Good
CR85	Mehta et. al. (2019)	NA	Descriptive	Good	Good
CR86	Mohandoss et. al. (2021)	NA	Descriptive	Good	Good
CR87	Mohanty et. al. (2019)	NA	Descriptive	Good	Good
CR88	Mohanty et. al. (2021a)	NA	Descriptive	Good	Good
CR89	Mohanty et. al. (2021b)	NA	Descriptive	Good	Good
CR90	Mondal et. al. (2022)	NA	Descriptive	Good	Good
CR91	Munigangaiah et. al. (2016)	NA	Descriptive	Good	Good
CR92	Muranjan et. al. (2016)	NA	Descriptive	Good	Good
CR93	Naik et. al. (2022)	NA	Descriptive	Good	Good
CR94	Narayanasamy et. al. (2020)	NA	Descriptive	Good	Good
CR95	Naveen et. al. (2020)	NA	Descriptive	Good	Good
CR96	Neyaz et. al. (2016)	NA	Descriptive	Good	Good
CR97	Pandey et. al. (2006)	NA	Descriptive	Good	Good
CR98	Pandey et. al. (2022)	NA	Descriptive	Good	Good
CR99	Pannu et. al. (2020)	NA	Descriptive	Good	Good
CR100	Pavithran et. al. (2004)	NA	Descriptive	Good	Good
CR101	Pazhaniyandi et. al. (2015)	NA	Descriptive	Good	Good
CR102	Pradeesh et. al. (2022)	NA	Descriptive	Good	Good
CR103	Princess et. al. (2018)	NA	Descriptive	Good	Good
CR104	Ray et. al. (2016)	NA	Descriptive	Good	Good
CR105	Rishi et. al. (2018)	NA	Descriptive	Good	Good
CR106	Saha et. al. (2018)	NA	Descriptive	Good	Good
CR107	Sahu et. al. (2021)	NA	Descriptive	Good	Good
CR108	Saifudheen et. al. (2012)	NA	Descriptive	Good	Good
CR109	Sankuratri et. al. (2015)	NA	Descriptive	Good	Good
CR110	Sarkar et. al. (2016)	NA	Descriptive	Good	Good
CR111	Sawale et. al. (2014)	NA	Descriptive	Good	Good
CR112	Shanmugapriya et. al. (2014)	NA	Descriptive	Good	Good
CR113	Sharda et. al. (2016)	NA	Descriptive	Good	Good
CR114	Sharma et. al. (2020)	NA	Descriptive	Good	Good
CR115	Singh (2004)	NA	Descriptive	Good	Good
CR116	Singh et. al. (2014)	NA	Descriptive	Good	Good
CR117	Singh et. al. (2021)	NA	Descriptive	Good	Good
CR118	Sundriyal et. al. (2013)	NA	Descriptive	Good	Good
CR119	Tandon et. al. (2019)	NA	Descriptive	Good	Good
CR120	Thakur et. al. (2022)	NA	Descriptive	Good	Good
CR121	Tomar et. al. (2021)	NA	Descriptive	Good	Good
CR122	Vajpayee et. al. (2017)	Neonates (Hospital)	Descriptive	Good	Good
CR123	Vasireddy et. al. (2023)	NA	Descriptive	Good	Good
CR124	Venketesan et. al. (2019)	NA	Descriptive	Good	Good
CR125	Verma et. al. (2017)	NA	Descriptive	Good	Good
CR126	Ahmad et. al. (2010)	NA	Symptoms...	Good	Good
CR127	Bhat et. al. (2016)	Children (Hospitals)	Symptoms...	Good	Good
CR128	Biswal et. al. (2018)	All Patients (Hospital)	Symptoms...	Good	Good
CR129	Isaac et al. (2004)	All Patients (Hospital)	Prevalence	Good	Good
CR130	Kaur et. al. (2020)	Children (Hospitals)	Symptoms...	Good	Good

CR131	Kawali et. al. (2023)	All Patients (Hospital)	Prevalence	Good	Good
CR132	Krishna et. al. (2015)	Children (Hospitals)	Symptoms…	Good	Good
CR133	Kumar et al. (2015)	Pregnant Women (Hospital)	Descriptive	Good	Good
CR134	Mahajan et. al. (2006a)	All Patients (Hospital)	Symptoms…	Good	Good
CR135	Mahajan et. al. (2010)	All Patients (Hospital)	Symptoms…	Good	Good
CR136	Mathai et al. (2003)	All Patients (Hospital)	Symptoms…	Good	Good
CR137	Meena et. al. (2015)	Pregnant Women	Symptoms…	Good	Good
CR138	Nanda et. al. (2014)	All Patients (Hospital)	Symptoms…	Good	Good
CR139	Rama et. al. (2019)	All Patients (Hospital)	Symptoms…	Good	Good
CR140	Razak et. al. (2010)	NA	Symptoms…	Good	Good
CR141	Sardana et. al. (2020)	All Patients (Hospital)	Symptoms…	Good	Good
CR142	Sarma et. al. (2017)	All Patients (Hospital)	Symptoms…	Good	Good
CR143	Sethi et. al. (2014)	All Patients (Hospital)	Symptoms…	Good	Good
CR144	Vikas et. al. (2023)	All Patients (Hospital)	Symptoms…	Good	Good
CR145	Vindhiya et. al. (2023)	Neonates	Symptoms…	Good	Good
CR146	Zainab et. al. (2018)	Children (Hospitals)	Symptoms…	Good	Good
CC1	Borkakoty et. al. (2016)	All Patients (Hospital)	Symptoms…	Fair	Fair
CC2	Govindaraj et. al. (2024)	All Patients (Hospital)	Prevalence	Fair	Poor
CC3	Mittal et. al. (2017)	All Patients (Hospital)	Symptoms…	Fair	Poor
CC4	Thakur et al. (2011)	All Patients (Hospital)	Prevalence + Symptoms…	Fair	Poor
CC5	Thangaraj et al. (2018)	Children (Hospitals)	Prevalence + Symptoms…	Good	Fair
CC6	Varghese et al. (2016)	All Patients (Hospital)	Symptoms…	Poor	Poor
CC7	Karthika et. al. (2021)	Children (Hospitals)	Prevalence + Symptoms…	Good	Fair
CC8	Varghese et. al. (2023)	Adult Patients >15 (Hospital)	Descriptive	Good	Good
CC9	Damodar et. al. (2023)	Children (Hospitals)	Prevalence + Symptoms…	Fair	Fair
CC10	Koralur et. al. 2018	Adult Patients (Hospital)	Prevalence	Fair	Fair
CC11	Mahajan et. al. (2006b)	All Patients (Hospital)	Symptoms…	Fair	Poor
CC12	Anitharaj et. al. (2020)	All Patients (Hospital)	Symptoms…	Fair	Fair
CC13	Koraluru et. al. (2015)	All Patients (Hospital)	Prevalence	Fair	Poor
CC14	Prakash et. al. (2006)	All Patients (Hospital)	Prevalence	Poor	Poor
CS1	Chunchanur et. al. (2020)	All Patients (Hospital)	Prevalence	Fair	Poor
CS2	Farhana et al. (2016)	All Patients (Hospital)	Prevalence	Fair	Fair
CS3	Jose et. al. (2022)	Children (Hospitals) (<15)	Prevalence	Fair	Fair
CS4	Khemka et al. (2021)	Children <12 (Hospitals)	Prevalence + Symptoms…	Good	Fair
CS5	Varghese et. al. (2013)	Adult Patients (Hospital)	Symptoms…	Good	Fair
CS6	Misra et. al. (2014)	All Patients (Hospital)	Symptoms…	Good	Fair
CS7	Narayanasamy et al. (2016)	Children (Hospitals)	Prevalence + Symptoms…	Fair	Fair
CS8	Palanivel et. al. (2012)	Children (Hospitals)	Symptoms…	Fair	Fair
CS9	Ramachandran et. al. (2023)	All Patients (Hospital)	Symptoms…	Good	Fair
CS10	Ramasamy et. al. (2023)	Children <12 (Hospitals)	Symptoms…	Fair	Fair
CS11	Takhar et al. (2017)	All Patients (Hospital)	Prevalence + Symptoms…	Fair	Fair
CS12	Aggarwal et al. (2015)	Adult Patients (Hospital)	Prevalence + Symptoms…	Fair	Fair
CS13	Bal et al. (2019)	Children (Hospitals)	Prevalence + Symptoms…	Fair	Fair
CS14	Bal et al. (2021)	All Patients (Hospital)	Prevalence + Symptoms…	Good	Fair
CS15	Bharathi et. al. (2023)	All Patients (Hospital)	Prevalence	Fair	Poor
CS16	Bhise et al. (2020)	All Patients (Hospital)	Prevalence + Symptoms…	Fair	Poor
CS17	Fomda et. al. (2023)	Community	Prevalence	Good	Fair
CS18	Khan et. al. (2012)	All Patients (Hospital)	Symptoms…	Fair	Poor
CS19	Narang et. al. (2022)	All Patients (Hospital)	Prevalence	Good	Poor
CS20	Panigrahi et. al. (2023)	All Patients (Hospital)	Prevalence	Fair	Fair
CS21	Paulraj et al. (2021)	All Patients (Hospital)	Prevalence + Symptoms…	Fair	Fair
CS22	Rawat et al. (2018)	All Patients (Hospital)	Prevalence + Symptoms…	Fair	Fair
CS23	Sinha et al. (2014)		Prevalence + Symptoms…	Fair	Fair

CS24	Stephen et al. (2015)	All Patients (Hospital)	Prevalence + Symptoms…	Fair	Fair
CS25	Tarai et. al. (2022)	All Patients (Hospital)	Prevalence	Fair	Fair
CS26	Thakur et. al. (2019)	All Patients (Hospital)	Symptoms…	Good	Fair
CS27	Vanramliana et. al. (2023)	All Patients (Hospital)	Prevalence	Fair	Fair
CS28	Agrawal et. al. (2022)	Children (Hospitals) (<18)	Symptoms…	Fair	Fair
CS29	Alam et. al. (2022)	All Patients (Hospital)	Symptoms…	Fair	Fair
CS30	Arun Babu et al. (2017)	Children < 12 (Hospitals)	Prevalence + Symptoms…	Fair	Fair
CS31	Barnabas et. al. (2021)	Adult Patients (Hospital)	Prevalence + Symptoms…	Fair	Fair
CS32	Basu et al. (2019)	Children <12 (Hospitals)	Prevalence + Symptoms…	Fair	Fair
CS33	Basu et al. (2021)	Children <12 (Hospitals)	Symptoms…	Fair	Fair
CS34	Bhat et. al. (2014a)	Children (Hospitals)	Symptoms…	Fair	Fair
CS35	Bhat et. al. (2014b)	Children (Hospitals)	Symptoms…	Fair	Fair
CS36	Boorugu et al. (2014)	All Patients (Hospital)	Prevalence + Symptoms…	Fair	Fair
CS37	Chrispal et al. (2010)	Adult Patients (Hospital)	Prevalence + Symptoms…	Fair	Fair
CS38	Chrispal et. al. (2010)	Adult Patients (Hospital)	Symptoms…	Fair	Fair
CS39	Das et. al. (2021)	All Patients (Hospital)	Prevalence	Fair	Fair
CS40	Dave et al. (2022)	All Patients (Hospital)	Prevalence + Symptoms…	Fair	Fair
CS41	Gaba et al. (2019)	All Patients (Hospital)	Prevalence + Symptoms…	Fair	Fair
CS42	Ganesh et. al. (2018)	Children (Hospitals)	Prevalence + Symptoms…	Fair	Fair
CS43	Grover et. al. (2021)	All Patients (Hospital)	Symptoms…	Fair	Fair
CS44	Jain et al. (2019)	All Patients >14 (Hospital)	Prevalence + Symptoms…	Fair	Fair
CS45	Jakharia et al. (2016)	All Patients (Hospital)	Prevalence	Fair	Fair
CS46	Jamil et. al. (2014)	Adult Patients (Hospital)	Symptoms…	Fair	Fair
CS47	Jamil et. al. (2015)	All Patients (Hospital)	Symptoms…	Fair	Fair
CS48	Jana et. al. (2023)	Children <12 (Hospitals)	Symptoms…	Fair	Fair
CS49	Kalal et. al. (2016)	Children <18 (Hospitals)	Symptoms…	Fair	Fair
CS50	Kalita et. al. (2015)	All Patients (Hospital)	Symptoms…	Fair	Fair
CS51	Kalita et. al. (2016)	All Patients (Hospital)	Symptoms…	Fair	Fair
CS52	Kamarasu et. al. (2007)	Community Survey	Prevalence	Fair	Fair
CS53	Kumar et al. (2012)	Children (Hospitals)	Symptoms…	Fair	Fair
CS54	Kumar et al. (2018b)	All Patients (Hospital)	Symptoms…	Fair	Fair
CS55	Kumar et. al. (2018)	All Patients (Hospital)	Prevalence	Fair	Fair
CS56	Loomba et. al. (2014)	All Patients (Hospital)	Symptoms…	Fair	Fair
CS57	Mokta et. al. (2017)	Adult Patients (Hospital)	Prevalence + Symptoms…	Fair	Fair
CS58	Morch et. al. (2017)	All Patients (Hospital)	Prevalence	Fair	Fair
CS59	Morch et. al. (2022)	All Patients (Hospital)	Prevalence	Fair	Fair
CS60	Murmu et. al. (2024)	All Patients (Hospital)	Prevalence + Symptoms…	Fair	Fair
CS61	Mutkule et al. (2015)	All Patients (Hospital)	Prevalence + Symptoms…	Fair	Fair
CS62	Rauf et. al. (2018)	Children <12 (Hospitals)	Prevalence + Symptoms…	Fair	Fair
CS63	Rehani et. al. (2024)	All Patients (Hospital)	Symptoms…	Fair	Fair
CS64	Roychowdhary et. al. (2022)	Adult Patients (Hospital)	Symptoms…	Fair	Fair
CS65	Sarangi et. al. (2016)	Children <14 (Hospitals)	Prevalence	Fair	Fair
CS66	Sharma et al. (2005)	All Patients (Hospital)	Prevalence	Fair	Fair
CS67	Singla et. al. (2020)	Children (Hospitals)	Symptoms…	Fair	Fair
CS68	Sivarajan et al. (2016)	All Patients (Hospital)	Prevalence + Symptoms…	Fair	Fair
CS69	Somashekar et al. (2006)	Children < 14 (Hospitals)	Prevalence + Symptoms…	Fair	Fair
CS70	Sood et al. (2016)	Children (Hospitals)	Prevalence + Symptoms…	Fair	Fair
CS71	Stephen et. al. (2013)	Adult Patients (Hospital)	Prevalence + Symptoms…	Fair	Fair
CS72	Stephen et. al. (2018)	All Patients (Hospital)	Symptoms…	Fair	Fair
CS73	Sultan et. al. (2022)	Adult Patients (Hospital)	Prevalence + Symptoms…	Fair	Fair

CS74	Varghese et al. (2006)	All Patients (Hospital)	Prevalence + Symptoms…	Fair	Fair
CS75	Varghese et. al. (2015)	All Patients (Hospital)	Symptoms…	Fair	Fair
CS76	Verma et. al. (2021)	All Patients (Hospital)	Symptoms…	Fair	Fair
CS77	Williams et al. (2021)	Children (Hospitals)	Prevalence + Symptoms…	Fair	Fair
CS78	Yadav et. al. (2019)	Children (Hospitals)	Prevalence	Fair	Fair
CS79	Yaqoob et. al. (2020)	All Patients (Hospital)	Prevalence	Fair	Fair
CS80	Gurung et al. (2013)	All Patients (Hospital)	Prevalence + Symptoms…	Fair	Fair
CS81	Alam et. al. (2020)	Children (Hospitals)	Prevalence + Symptoms…	Fair	Fair
CS82	Baidya et. al. (2022)	Children <12 (Hospitals)	Prevalence + Symptoms…	Fair	Fair
CS83	Chunduru et. al. (2023)	All Patients (Hospital)	Symptoms…	Fair	Fair
CS84	Kumar et. al. (2021)	Adult Patients (Hospital)	Symptoms…	Fair	Fair
CS85	Anithraj et. al. (2016)	All Patients (Hospital)	Prevalence + Symptoms…	Fair	Fair
CS86	Balasubramanian et. al. (2016)	All Patients (Hospital)	Symptoms…	Fair	Fair
CS87	Behera et al. (2019)	All Patients (Hospital)	Prevalence + Symptoms…	Fair	Fair
CS88	Bhattacharya et. al. (2020)	Adult Patients (Hospital)	Symptoms…	Fair	Fair
CS89	Karanth et. al. (2014)	All Patients (Hospital)	Symptoms…	Fair	Fair
CS90	Koshy et. al. (2018)	All Patients (Hospital)	Symptoms…	Fair	Fair
CS91	Narayanasamy et. al (2018)	Children <12 (Hospitals)	Prevalence + Symptoms…	Fair	Fair
CS92	Ramyasree et al. (2015)	Adult Patients (Hospital)	Prevalence + Symptoms…	Fair	Fair
CS93	Sharma et. al. (2015)	All Patients (Hospital)	Symptoms…	Fair	Fair
CS94	Shiva et. al. (2022)	Adult Patients (Hospital)	Symptoms…	Fair	Fair
CS95	Vaz & Gupta (2006)	Adult Patients (Hospital)	Prevalence + Symptoms…	Fair	Fair
CS96	Gulati et. al. (2021)	All Patients (Hospital)	Symptoms…	Fair	Fair
CS97	Abhilash et. al. (2015)	All Patients (Hospital)	Symptoms…	Fair	Fair
CS98	Agarwal et. al. (2014)	All Patients (Hospital)	Symptoms…	Fair	Fair
CS99	Ahmad et al. (2016)	All Patients (Hospital)	Prevalence + Symptoms…	Fair	Fair
CS100	Bansod et. al. (2021)	All Patients (Hospital)	Symptoms…	Fair	Fair
CS101	Basheer et. al. (2016)	All Patients (Hospital)	Symptoms…	Fair	Fair
CS102	Behera et al. (2021)	Children (Hospitals) (<14)	Prevalence + Symptoms…	Fair	Fair
CS103	Bhargava et. al. (2016)	All Patients (Hospital)	Symptoms…	Fair	Fair
CS104	Bhattacharya et. al. (2019)	Adult Patients (Hospital)	Symptoms…	Fair	Fair
CS105	Dass et. al. (2011)	Children (Hospitals)	Symptoms…	Fair	Fair
CS106	Dhar et. al. (2018)	Adult Patients (Hospital)	Symptoms…	Fair	Fair
CS107	Jayaprakash et. al. (2019)	All Patients (Hospital)	Symptoms…	Fair	Fair
CS108	John et. al. (2023)	All Patients (Hospital)	Prevalence + Symptoms…	Fair	Fair
CS109	Kakarlapudi et. al. (2018)	Children (Hospitals)	Prevalence + Symptoms…	Fair	Fair
CS110	Khan et. al. (2017)	All Patients (Hospital)	Prevalence + Symptoms…	Fair	Fair
CS111	Kispotta et al. (2020)	Children <12 (Hospitals)	Prevalence + Symptoms…	Fair	Fair
CS112	Krishnan et. al. (2016)	Children <12 (Hospitals)	Symptoms…	Fair	Fair
CS113	Kumar et al. (2014)	All Patients (Hospital)	Prevalence + Symptoms…	Fair	Fair
CS114	Kumar et al. (2018a)	Children <12 (Hospitals)	Symptoms…	Fair	Fair
CS115	Lakshmi et al. (2020)	All Patients (Hospital)	Prevalence + Symptoms…	Fair	Fair
CS116	Mahajan et. al. (2008)	Children (Hospitals)	Symptoms…	Fair	Fair
CS117	Mahajan et. al. (2016a)	All Patients (Hospital)	Symptoms…	Fair	Fair
CS118	Masand et. al. (2016)	Children (Hospitals)	Symptoms…	Fair	Fair
CS119	Muthukrishnan et. al. (2020)	Children (Hospitals)	Symptoms…	Fair	Fair
CS120	Nallasamy et. al. (2020)	Children (Hospitals)	Symptoms…	Fair	Fair
CS121	Narayanasamy et. al (2021)	Children (Hospitals)	Symptoms…	Fair	Fair
CS122	Narayanasamy et. al (2023)	Children (Hospitals)	Symptoms…	Fair	Fair

CS123	Narvencar et al. (2012)	Adult Patients (Hospital)	Prevalence + Symptoms...	Fair	Fair
CS124	Peter et. al. (2013)	All Patients (Hospital)	Prevalence	Fair	Fair
CS125	Pichamuthu et al. (2014)	All Patients (Hospital)	Prevalence + Symptoms...	Fair	Fair
CS126	Poomalar & Rekha (2014)	Prenatal and postnatal women (Hospital)	Prevalence + Symptoms...	Fair	Fair
CS127	Rajan et al. (2016)	Pregnant Women (Hospital)	Descriptive	Fair	Fair
CS128	Ralph et. al. (2019)	Adult Patients > 16 (Hospital)	Symptoms...	Fair	Fair
CS129	Rana et. al. (2017)	Adult Patients (Hospital)	Symptoms...	Fair	Fair
CS130	Rathi et al. (2011)	Children (Hospitals)	Prevalence + Symptoms...	Fair	Fair
CS131	Rose et. al. (2017)	Children (Hospitals)	Symptoms...	Fair	Fair
CS132	Saha et al. (2018)	Children (Hospitals)	Prevalence + Symptoms...	Fair	Fair
CS133	Sankhyan et. al. (2014)	Children (Hospitals)	Prevalence + Symptoms...	Fair	Fair
CS134	Sengupta et. al. (2014)	Pregnant Women (Hospital)	Symptoms...	Fair	Fair
CS135	Sharma et. al. (2016)	All Patients (Hospital)	Symptoms...	Fair	Fair
CS136	Sivaprakasam et. al. (2020)	Children (Hospitals)	Symptoms...	Fair	Fair
CS137	Sivathanu et. al. (2017)	Children <12 (Hospitals)	Symptoms...	Fair	Fair
CS138	Subbalaxmi et. al. (2014)	All Patients (Hospital)	Symptoms...	Fair	Fair
CS139	Valappil et. al. (2017)	All Patients (Hospital)	Symptoms...	Fair	Fair
CS140	Varghese et al. (2013)	All Patients (Hospital)	Symptoms...	Fair	Fair
CS141	Varghese et. al. (2014)	All Patients (Hospital)	Symptoms...	Fair	Fair
CS142	Veerappan et. al. (2021)	Children (Hospitals)	Symptoms...	Fair	Fair
CS143	Vikrant et al. (2013)	All Patients (Hospital)	Prevalence + Symptoms...	Fair	Fair
CS144	Viswanathan et. al. (2013)	All Patients (Hospital)	Symptoms...	Fair	Fair
CS145	Vivekanandan et. al. (2010)	All Patients (Hospital)	Symptoms...	Fair	Fair
CS146	Yadav et al. (2023)	Pregnant Women	Prevalence + Symptoms...	Fair	Fair
CS147	Khan et. al. (2016)	All Patients (Hospital)	Prevalence	Fair	Fair
CS148	Mittal et al. (2012)	All Patients (Hospital)	Prevalence + Symptoms...	Fair	Fair
CS149	Oberoi & Varghese (2014)	All Patients (Hospital)	Prevalence + Symptoms...	Fair	Fair
CS150	Sengupta et. al.(2015)	Community	Prevalence	Fair	Fair
CS151	Suvarna et. al. (2023)	Community	Prevalence	Fair	Fair
CS152	Thangaraj et al. (2017)	All Patients (Hospital)	Prevalence + Symptoms...	Fair	Fair
CS153	Trowbridge et. al. (2017)	All Patients (Hospital)	Prevalence	Fair	Fair
CS154	Bithu et. al. (2014)	All Patients (Hospital)	Prevalence	Fair	Fair
CS155	Husain et. al. (2022)	All Patients (Hospital)	Prevalence	Fair	Fair
CS156	Shubham et. al. (2023)	All Patients (Hospital)	Prevalence	Fair	Fair
CS157	Vikram et al. (2020)	All Patients (Hospital)	Prevalence + Symptoms...	Fair	Fair

Supplementary Figures

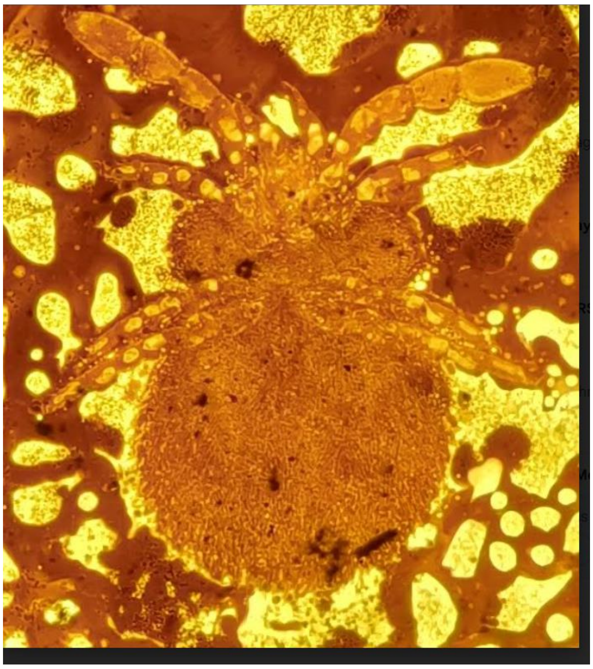

Supplementary figure 1. Larval stage of the Leptotrombidium mite, the primary vector for the *Orientia tsutsugamushi*, the etiological agent of scrub typhus. Obtained from archives of All India Institute of Medical Sciences (AIIMS), New Delhi

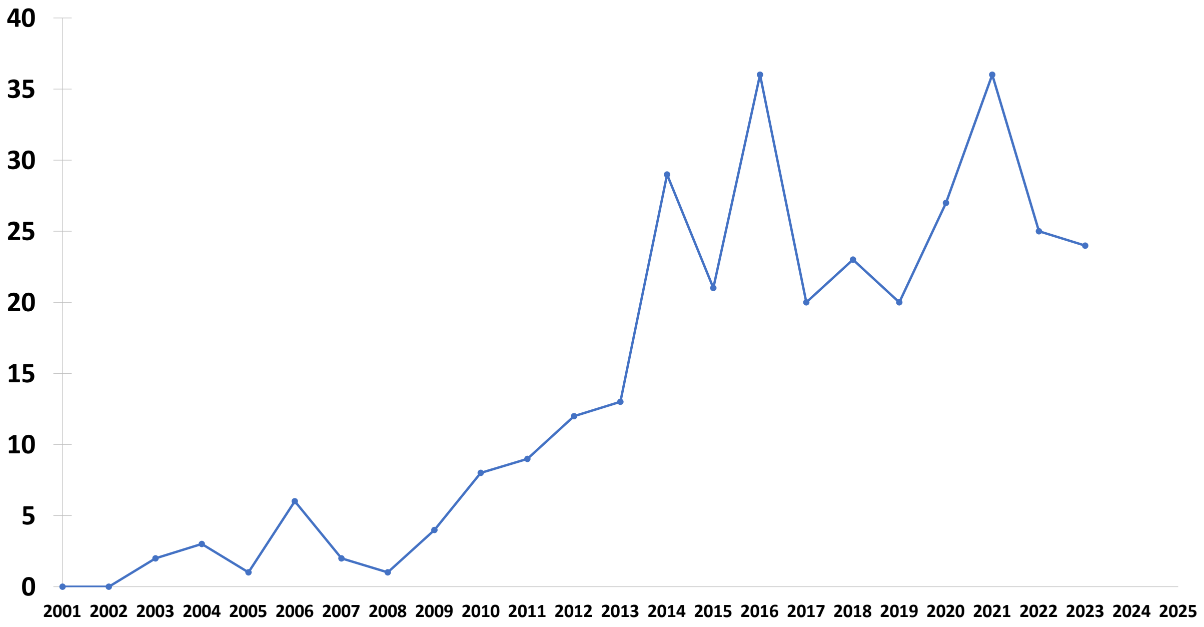

Supplementary figure 2. Total number of publications for scrub typhus over the years

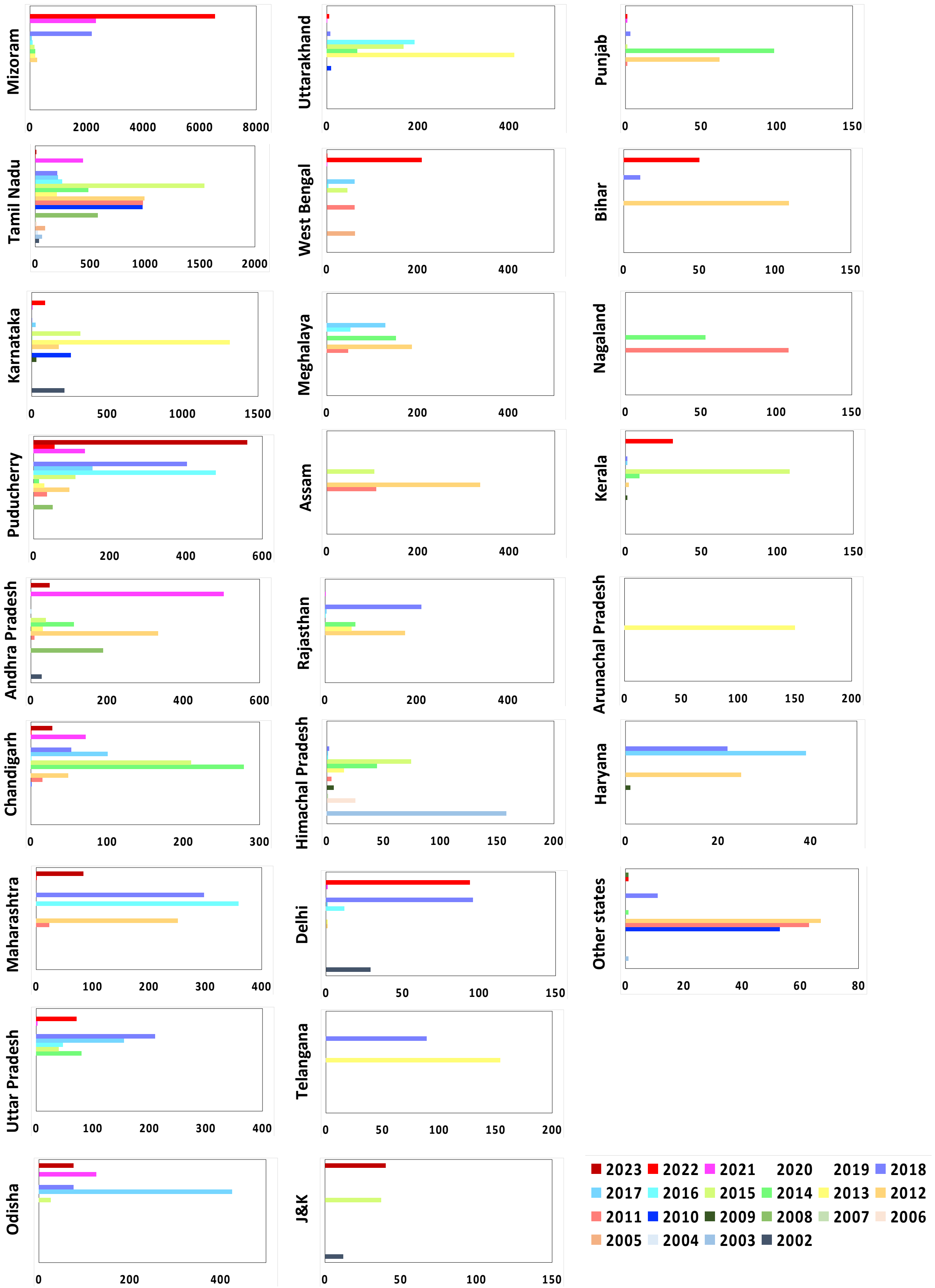

Supplementary Figure 3. Cumulative cases of scrub typhus in Indian states year wise. Other states which reported scrub typhus cases were Madhya Pradesh, Jharkhand, and Tripura. *The study year when the study was completed was considered.

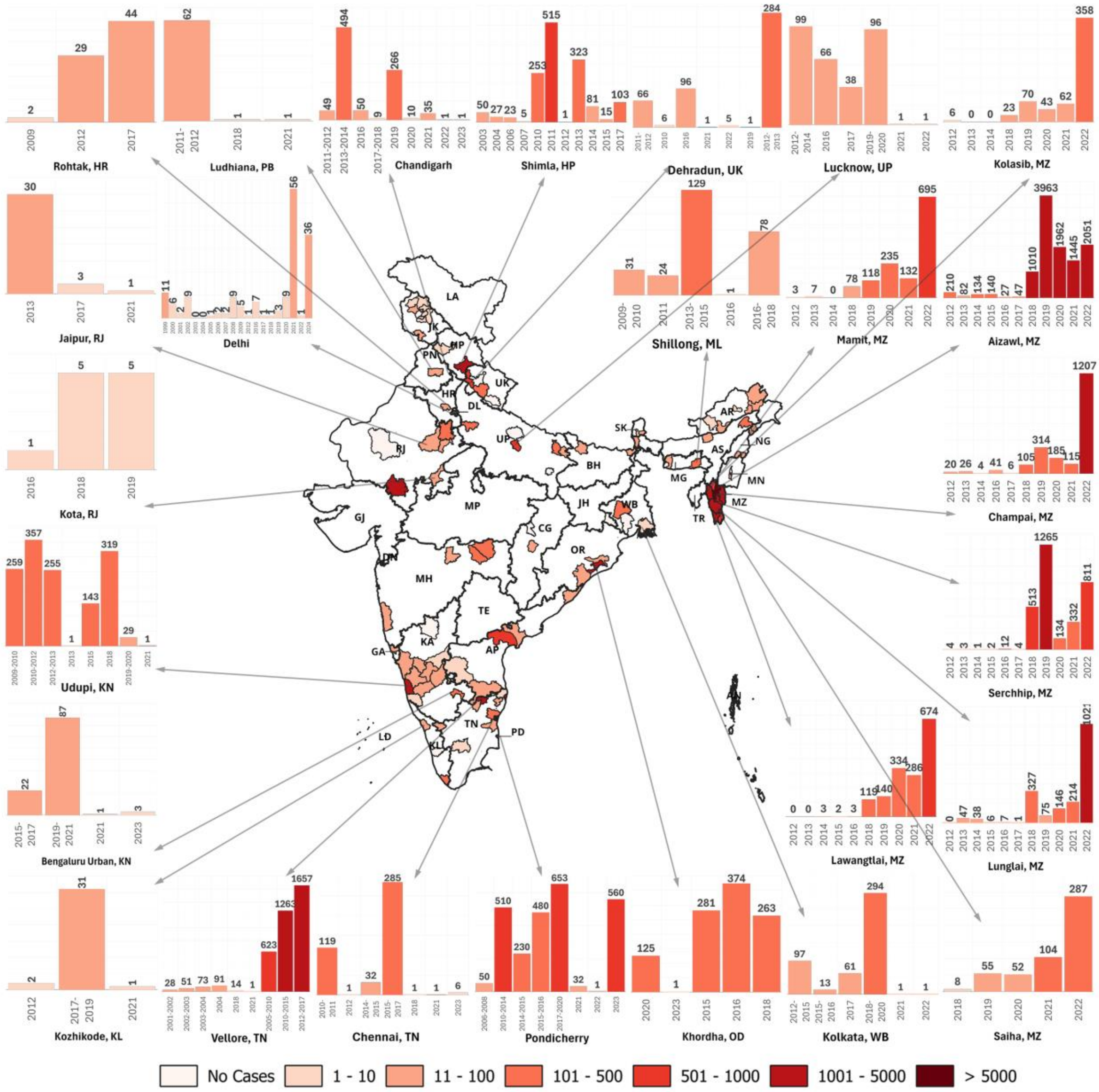

Supplementary Figure 4. District wise distribution of cumulative cases of scrub typhus as well as temporal trends in scrub typhus cases in districts where year-wise data was provided or in which more than one study was conducted at different time periods

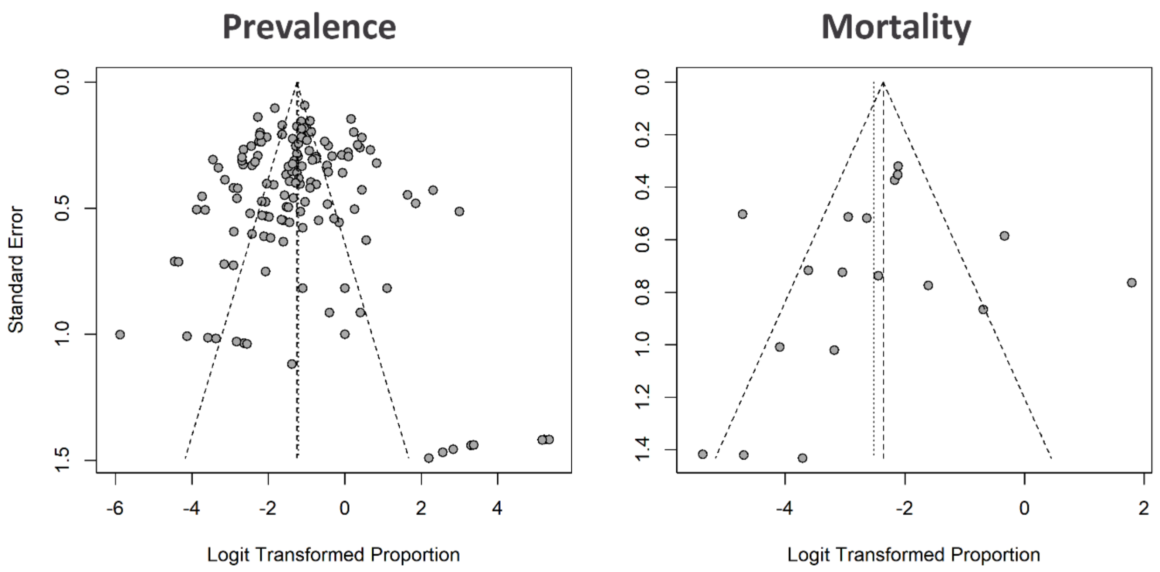

Supplementary Figure 5. Funnel plots for prevalence and mortality studies indicating publication bias (Symmetrical plots indicate low publication bias while asymmetrical plots indicate significant publication bias)

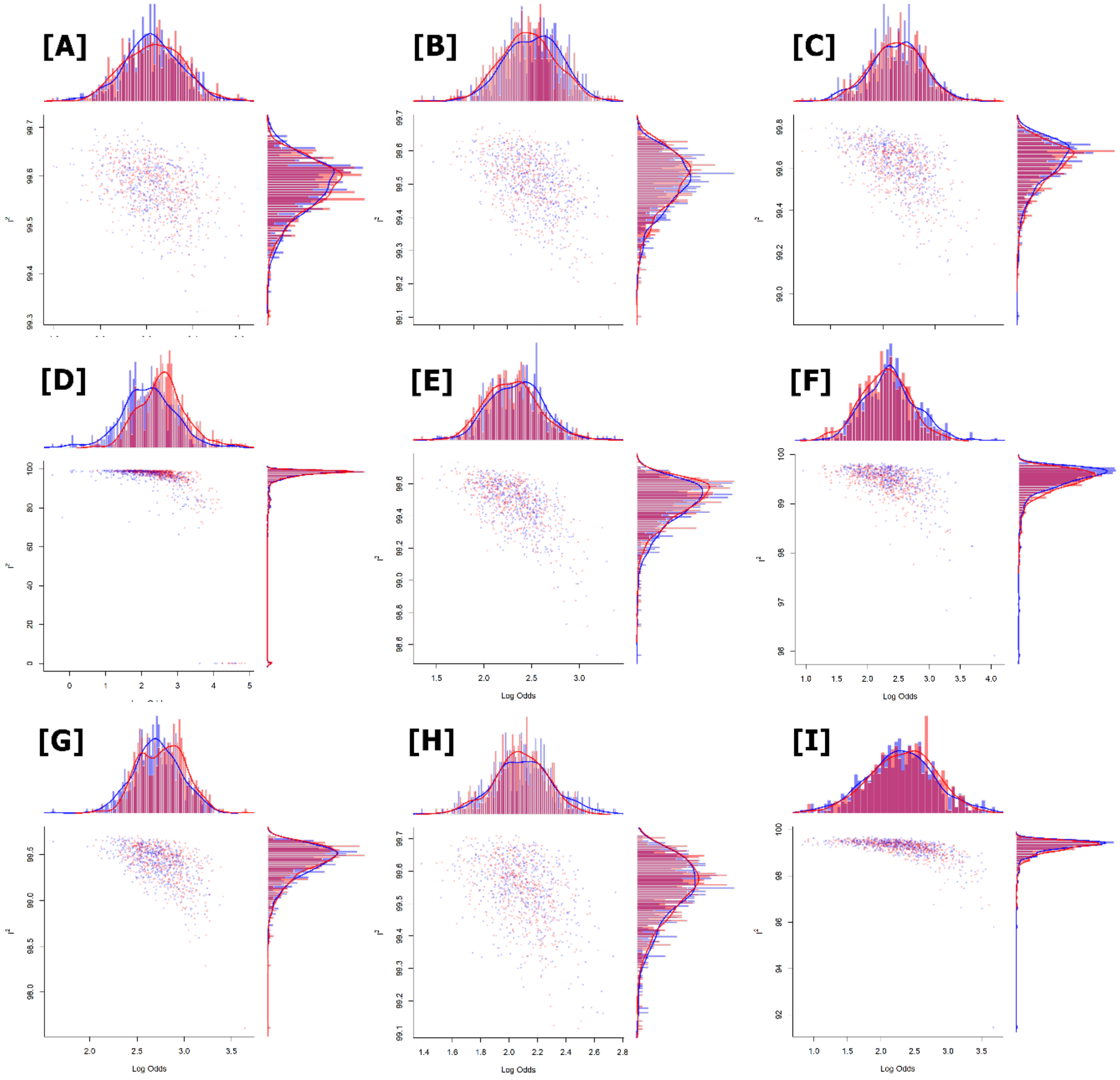

Supplementary Figure 6: Variations in the heterogeneity against the effect size for Symptoms – [A] General, [B] Gastro-intestinal, [C] Pulmonary, [D] Cardiac, [E] Hepatic, [F] Renal, [G] Neurological, [H] Inflammatory, [I] Others.

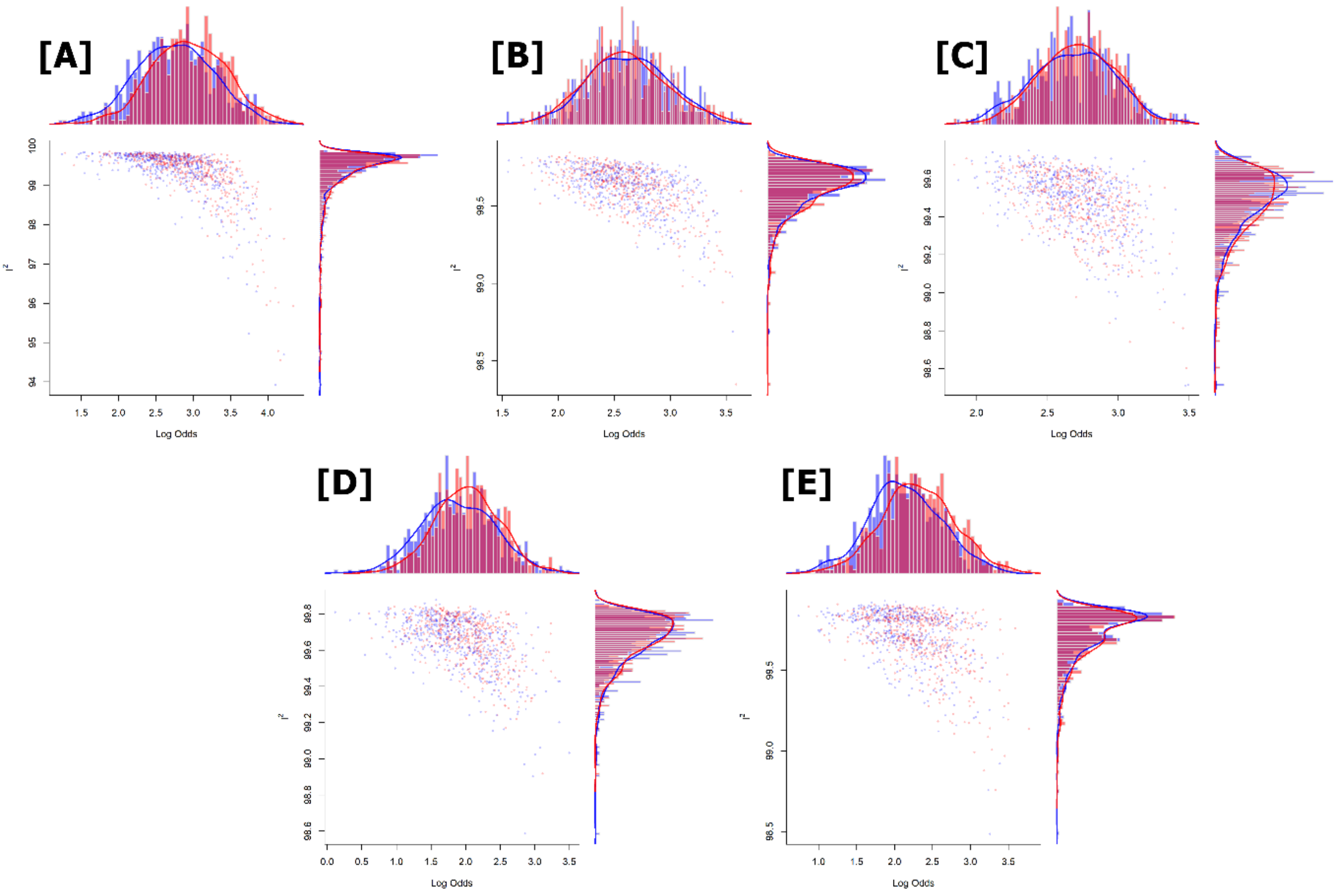

Supplementary Figure 7: Variations in the heterogeneity against the effect size for complications - [A] Cardiac, [B] Pulmonary, [C] Neurological, [D] Renal, [E] Others

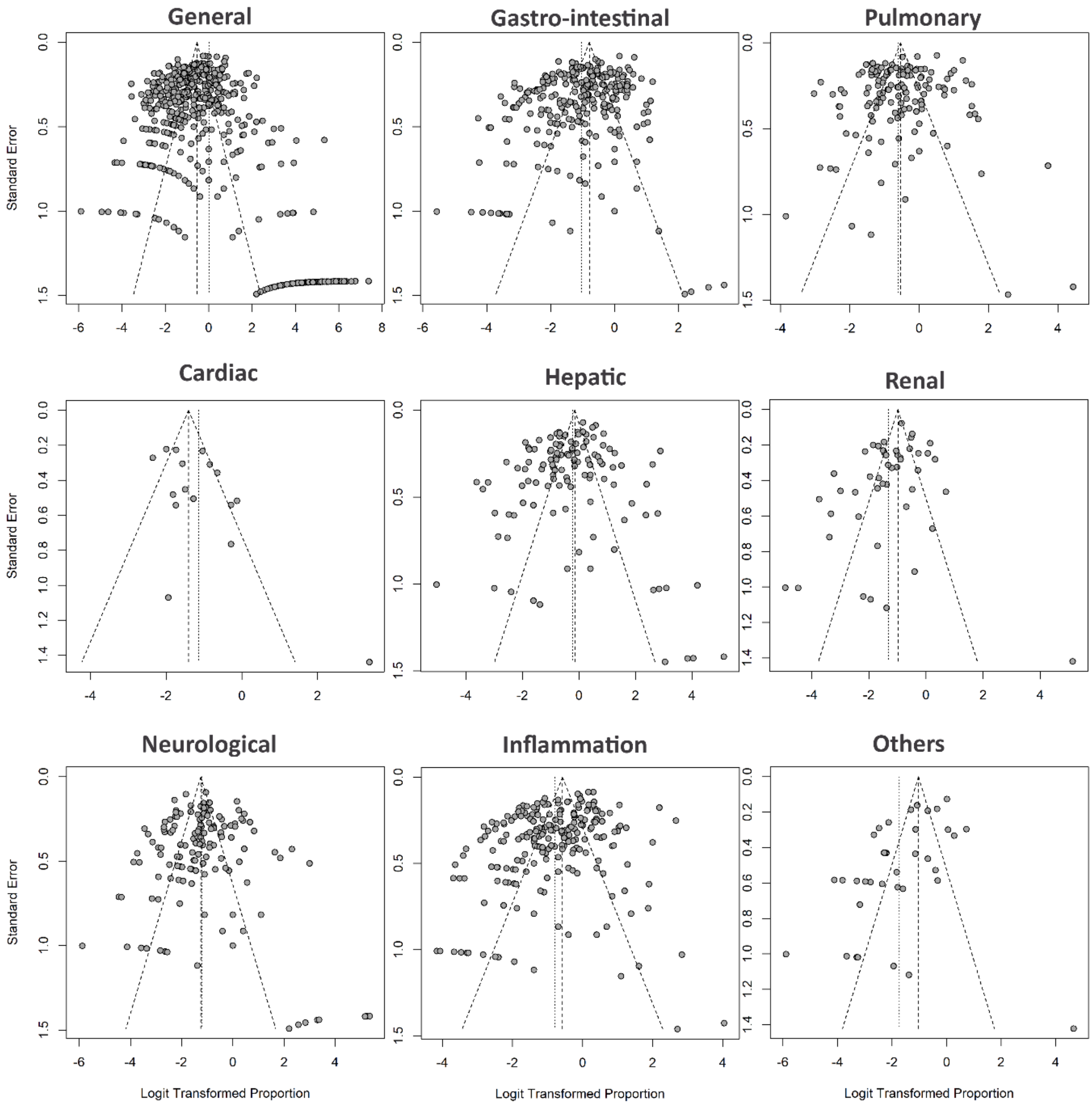

Supplementary Figure 8. Funnel plots indicating publication bias for different symptoms (Symmetrical plots indicate low publication bias while asymmetrical plots indicate significant publication bias)

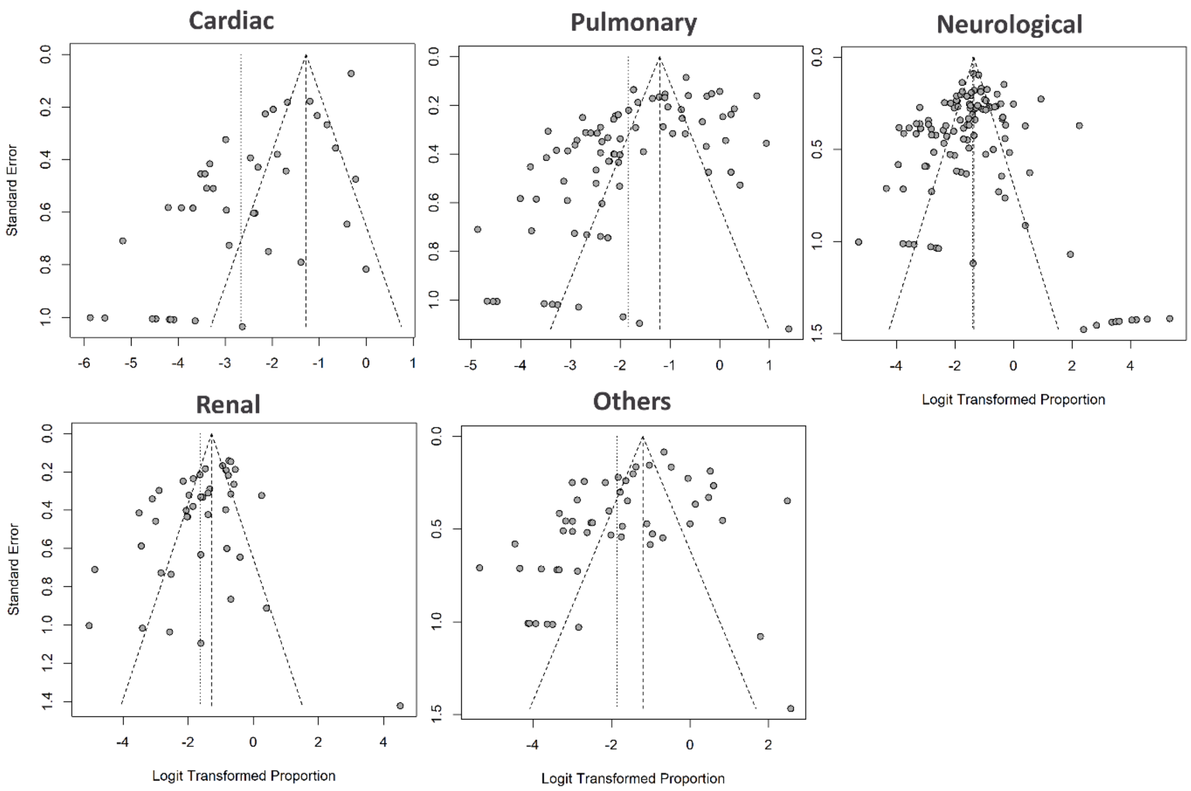

Supplementary Figure 9. Funnel plots indicating publication bias for different complications (Symmetrical plots indicate low publication bias while asymmetrical plots indicate significant publication bias)

Forest Plots

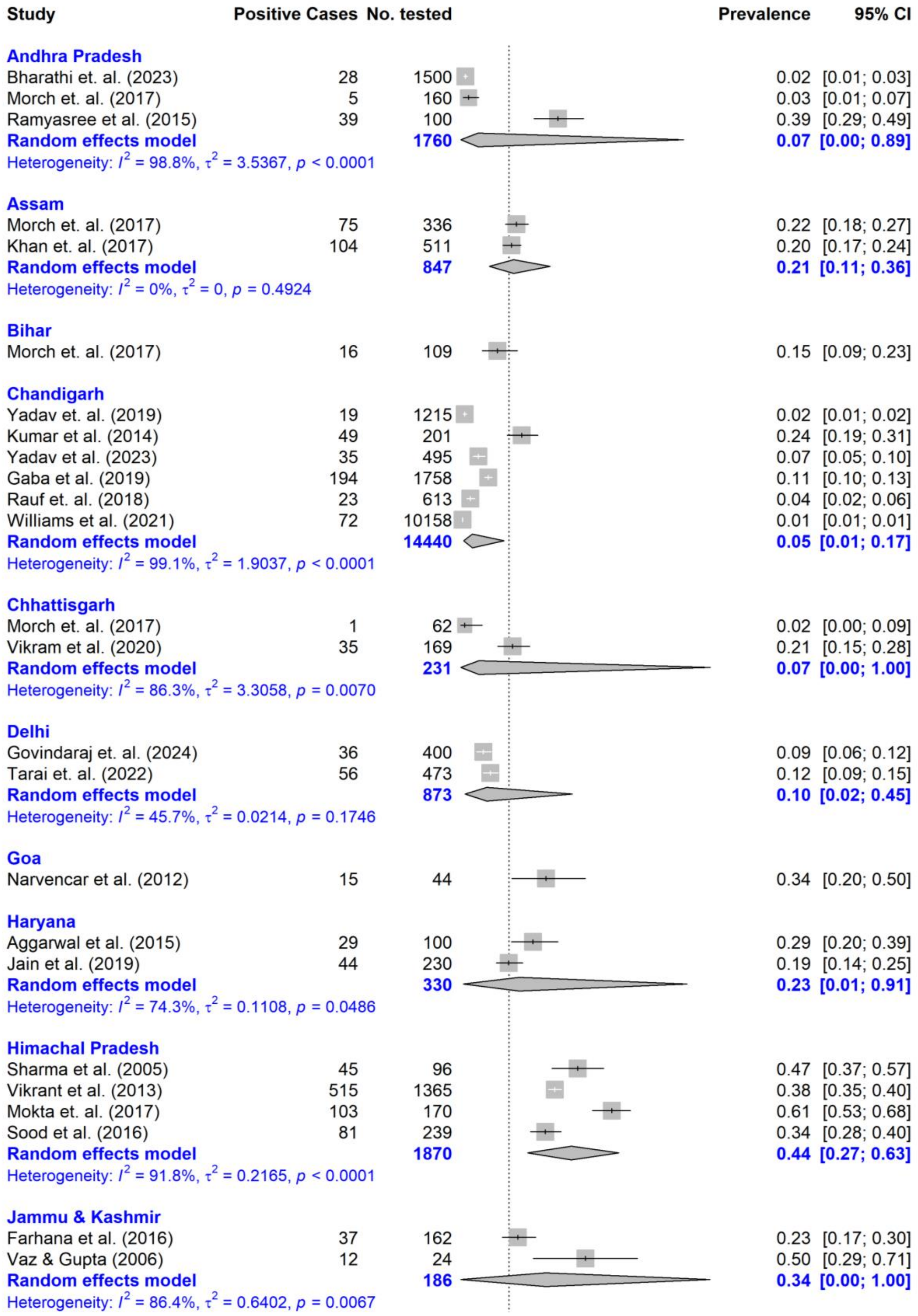
Study | Positive Cases | No. tested | Prevalence | 95% CI
Andhra Pradesh
Bharathi et. al. (2023) 28 1500 0.02 [0.01; 0.03]
Morch et. al. (2017) 5 160 0.03 [0.01; 0.07]
Ramyasree et al. (2015) 39 100 0.39 [0.29; 0.49]
Random effects model 1760 0.07 [0.00; 0.89]
Heterogeneity: I^2 = 98.8%, τ^2 = 3.5367, p < 0.0001
Assam
Morch et. al. (2017) 75 336 0.22 [0.18; 0.27]
Khan et. al. (2017) 104 511 0.20 [0.17; 0.24]
Random effects model 847 0.21 [0.11; 0.36]
Heterogeneity: I^2 = 0%, τ^2 = 0, p = 0.4924
Bihar
Morch et. al. (2017) 16 109 0.15 [0.09; 0.23]
Chandigarh
Yadav et. al. (2019) 19 1215 0.02 [0.01; 0.02]
Kumar et al. (2014) 49 201 0.24 [0.19; 0.31]
Yadav et al. (2023) 35 495 0.07 [0.05; 0.10]
Gaba et al. (2019) 194 1758 0.11 [0.10; 0.13]
Rauf et. al. (2018) 23 613 0.04 [0.02; 0.06]
Williams et al. (2021) 72 10158 0.01 [0.01; 0.01]
Random effects model 14440 0.05 [0.01; 0.17]
Heterogeneity: I^2 = 99.1%, τ^2 = 1.9037, p < 0.0001
Chhattisgarh
Morch et. al. (2017) 1 62 0.02 [0.00; 0.09]
Vikram et al. (2020) 35 169 0.21 [0.15; 0.28]
Random effects model 231 0.07 [0.00; 1.00]
Heterogeneity: I^2 = 86.3%, τ^2 = 3.3058, p = 0.0070
Delhi
Govindaraj et. al. (2024) 36 400 0.09 [0.06; 0.12]
Tarai et. al. (2022) 56 473 0.12 [0.09; 0.15]
Random effects model 873 0.10 [0.02; 0.45]
Heterogeneity: I^2 = 45.7%, τ^2 = 0.0214, p = 0.1746
Goa
Narvencar et al. (2012) 15 44 0.34 [0.20; 0.50]
Haryana
Aggarwal et al. (2015) 29 100 0.29 [0.20; 0.39]
Jain et al. (2019) 44 230 0.19 [0.14; 0.25]
Random effects model 330 0.23 [0.01; 0.91]
Heterogeneity: I^2 = 74.3%, τ^2 = 0.1108, p = 0.0486
Himachal Pradesh
Sharma et al. (2005) 45 96 0.47 [0.37; 0.57]
Vikrant et al. (2013) 515 1365 0.38 [0.35; 0.40]
Mokta et. al. (2017) 103 170 0.61 [0.53; 0.68]
Sood et al. (2016) 81 239 0.34 [0.28; 0.40]
Random effects model 1870 0.44 [0.27; 0.63]
Heterogeneity: I^2 = 91.8%, τ^2 = 0.2165, p < 0.0001
Jammu & Kashmir
Farhana et al. (2016) 37 162 0.23 [0.17; 0.30]
Vaz & Gupta (2006) 12 24 0.50 [0.29; 0.71]
Random effects model 186 0.34 [0.00; 1.00]
Heterogeneity: I^2 = 86.4%, τ^2 = 0.6402, p = 0.0067

**Karnataka**				
Kawali et. al. (2020)	13	163	0.08	[0.04; 0.13]
Koralur et. al. 2018	319	1036	0.31	[0.28; 0.34]
Koraluru et. al. (2015)	143	546	0.26	[0.23; 0.30]
Kawali et. al. (2023)	3	16	0.19	[0.04; 0.46]
Chunchanur et. al. (2019)	22	100	0.22	[0.14; 0.31]
Damodar et. al. (2023)	87	376	0.23	[0.19; 0.28]
**Random effects model**		**2237**	**0.21**	**[0.13; 0.33]**
Heterogeneity: $I^2 = 86.7\%$, $\tau^2 = 0.2641$, $p < 0.0001$				
**Kerala**				
Kumar et. al. (2018)	9	75	0.12	[0.06; 0.22]
**Maharashtra**				
Shubham et. al. (2023)	31	92	0.34	[0.24; 0.44]
Narang et. al. (2022)	267	1680	0.16	[0.14; 0.18]
Morch et. al. (2017)	20	251	0.08	[0.05; 0.12]
Rathi et al. (2011)	23	161	0.14	[0.09; 0.21]
Bhise et al. (2020)	158	490	0.32	[0.28; 0.37]
**Random effects model**		**2674**	**0.19**	**[0.08; 0.37]**
Heterogeneity: $I^2 = 95.8\%$, $\tau^2 = 0.5228$, $p < 0.0001$				
**Meghalaya**				
Sivarajan et al. (2016)	90	662	0.14	[0.11; 0.16]
**Nagaland**				
Khan et. al. (2016)	53	145	0.37	[0.29; 0.45]
**Odisha**				
Karthika et. al. (2021)	124	764	0.16	[0.14; 0.19]
Panigrahi et. al. (2023)	74	170	0.44	[0.36; 0.51]
Das et. al. (2021)	6	92	0.07	[0.02; 0.14]
Sarangi et. al. (2016)	26	71	0.37	[0.25; 0.49]
Behera et al. (2021)	76	171	0.44	[0.37; 0.52]
Bal et al. (2019)	201	413	0.49	[0.44; 0.54]
Bal et al. (2021)	45	140	0.32	[0.25; 0.41]
Behera et al. (2019)	114	432	0.26	[0.22; 0.31]
**Random effects model**		**2253**	**0.30**	**[0.17; 0.46]**
Heterogeneity: $I^2 = 96.1\%$, $\tau^2 = 0.6678$, $p < 0.0001$				
**Pondicherry**				
Anithraj et. al. (2016)	140	220	0.64	[0.57; 0.70]
Roopa et. al. (2015)	109	482	0.23	[0.19; 0.27]
Jose et. al. (2022)	51	318	0.16	[0.12; 0.21]
Poomalar & Rekha (2014)	340	659	0.52	[0.48; 0.55]
Stephen et al. (2015)	28	45	0.62	[0.47; 0.76]
Narayanasamy et al. (2016)	117	448	0.26	[0.22; 0.30]
Narayanasamy et. al (2018)	230	930	0.25	[0.22; 0.28]
Kispotta et al. (2020)	262	41365	0.01	[0.01; 0.01]
Stephen et. al. (2013)	28	58	0.48	[0.35; 0.62]
Arun Babu et al. (2017)	107	283	0.38	[0.32; 0.44]
Baidya et. al. (2022)	31	127	0.24	[0.17; 0.33]
**Random effects model**		**44935**	**0.27**	**[0.12; 0.52]**
Heterogeneity: $I^2 = 99.7\%$, $\tau^2 = 2.3719$, $p = 0$				
**Punjab**				
Oberoi & Varghese (2014)	98	772	0.13	[0.10; 0.15]
**Rajasthan**				
Sinha et al. (2014)	42	170	0.25	[0.18; 0.32]
Bithu et. al. (2014)	133	271	0.49	[0.43; 0.55]
Takhar et al. (2017)	66	290	0.23	[0.18; 0.28]
Dave et al. (2022)	1340	3814	0.35	[0.34; 0.37]
**Random effects model**		**4545**	**0.32**	**[0.17; 0.53]**
Heterogeneity: $I^2 = 93.9\%$, $\tau^2 = 0.2760$, $p < 0.0001$				

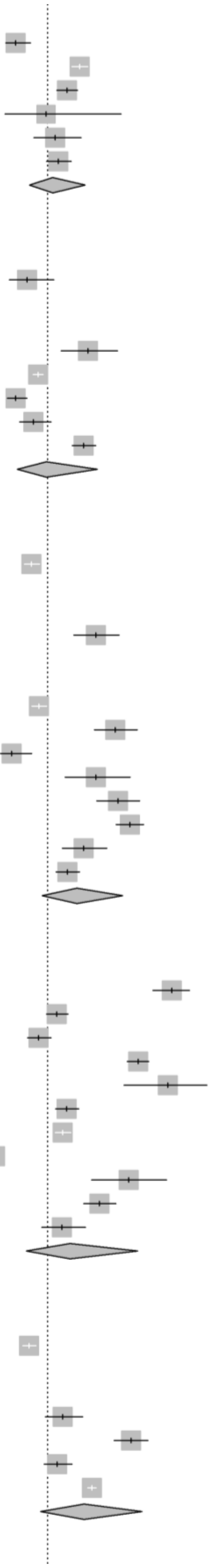

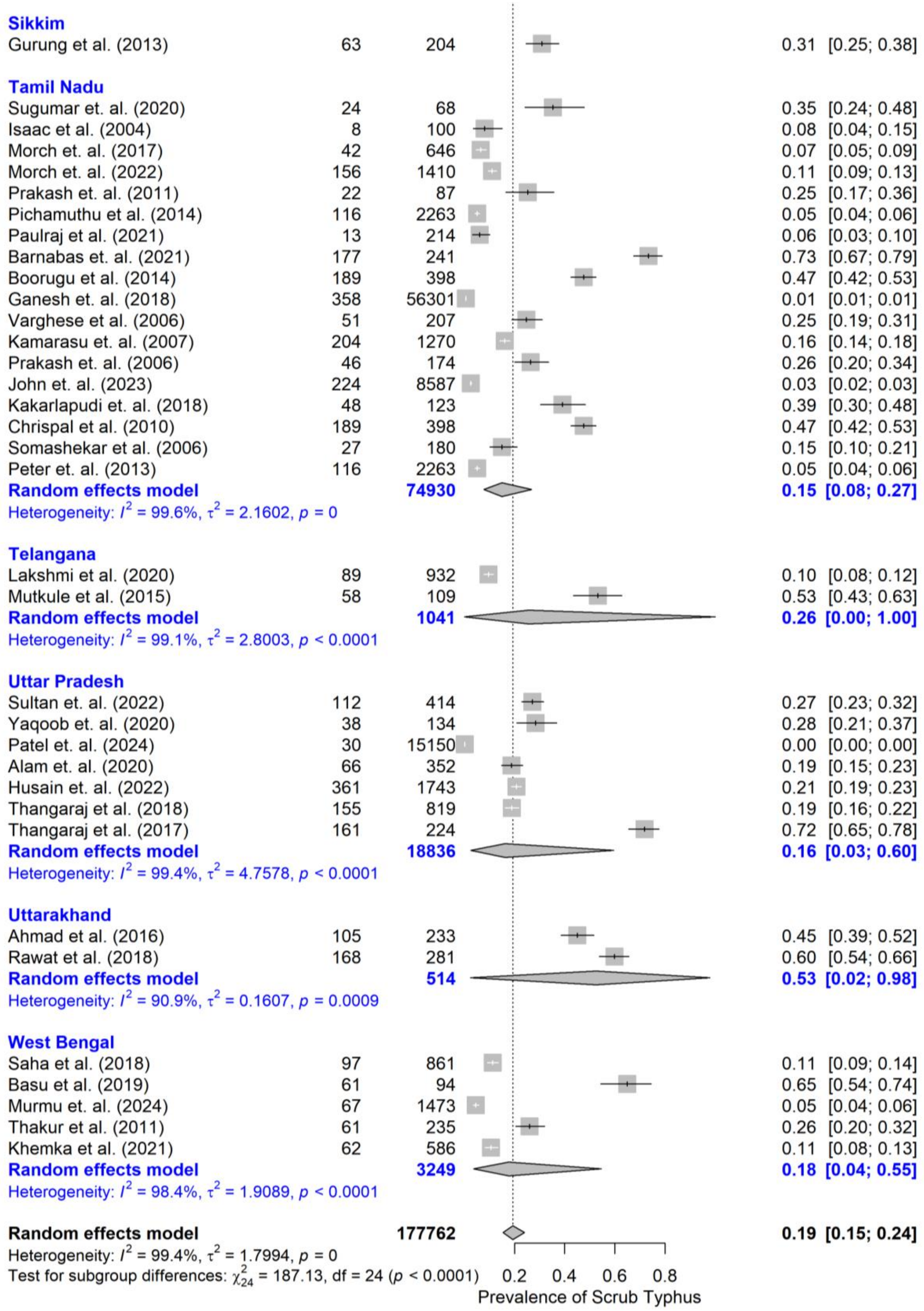

Forest Plot 1. Pooled prevalence of scrub typhus in different states of India using a random effects model

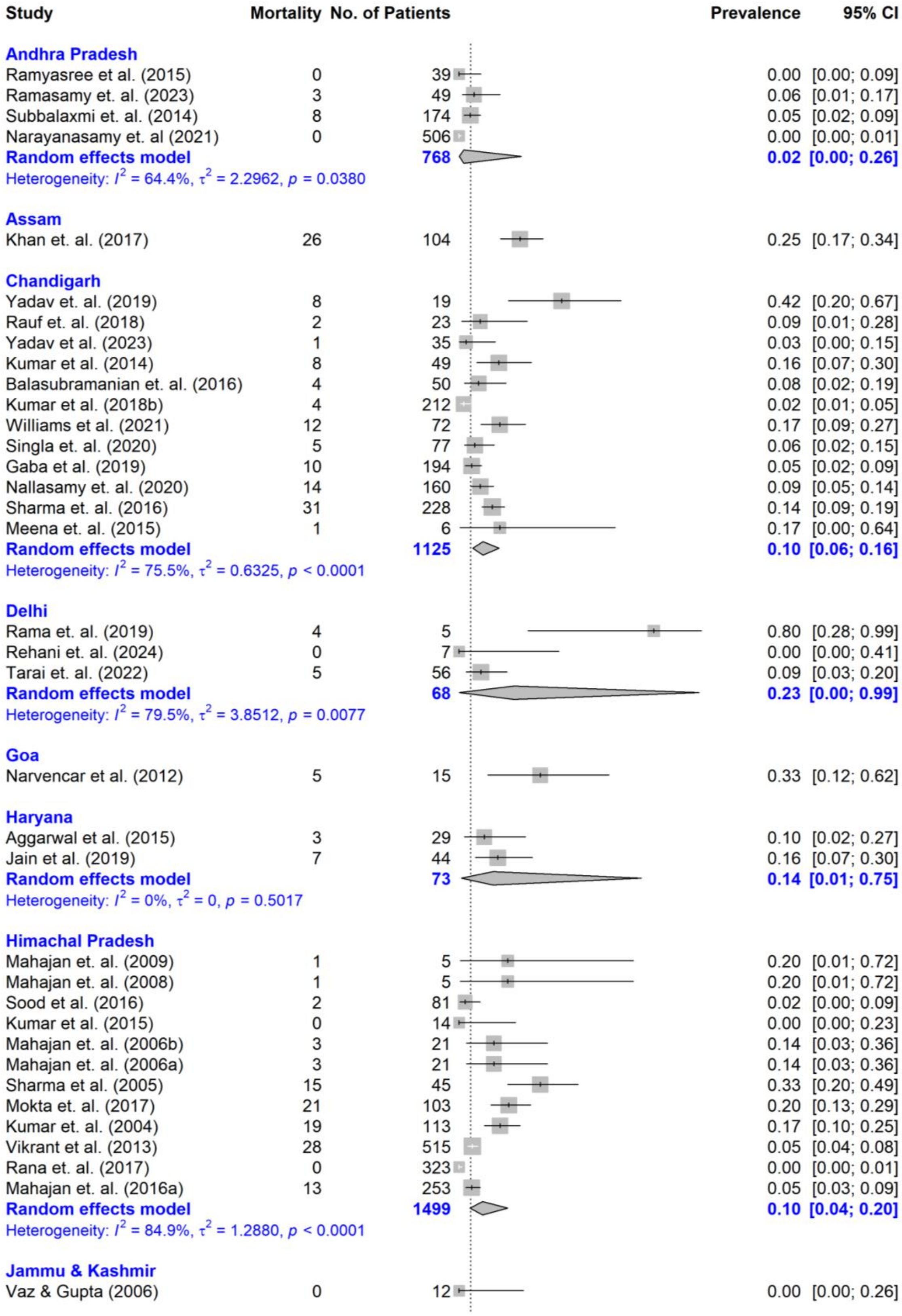
Study Mortality No. of Patients Prevalence 95% CI
Andhra Pradesh
Ramyasree et al. (2015) 0 39 0.00 [0.00; 0.09]
Ramasamy et. al. (2023) 3 49 0.06 [0.01; 0.17]
Subbalaxmi et. al. (2014) 8 174 0.05 [0.02; 0.09]
Narayanasamy et. al (2021) 0 506 0.00 [0.00; 0.01]
Random effects model 768 0.02 [0.00; 0.26]
Heterogeneity: I^2 = 64.4%, τ^2 = 2.2962, p = 0.0380
Assam
Khan et. al. (2017) 26 104 0.25 [0.17; 0.34]
Chandigarh
Yadav et. al. (2019) 8 19 0.42 [0.20; 0.67]
Rauf et. al. (2018) 2 23 0.09 [0.01; 0.28]
Yadav et al. (2023) 1 35 0.03 [0.00; 0.15]
Kumar et al. (2014) 8 49 0.16 [0.07; 0.30]
Balasubramanian et. al. (2016) 4 50 0.08 [0.02; 0.19]
Kumar et al. (2018b) 4 212 0.02 [0.01; 0.05]
Williams et al. (2021) 12 72 0.17 [0.09; 0.27]
Singla et. al. (2020) 5 77 0.06 [0.02; 0.15]
Gaba et al. (2019) 10 194 0.05 [0.02; 0.09]
Nallasamy et. al. (2020) 14 160 0.09 [0.05; 0.14]
Sharma et. al. (2016) 31 228 0.14 [0.09; 0.19]
Meena et. al. (2015) 1 6 0.17 [0.00; 0.64]
Random effects model 1125 0.10 [0.06; 0.16]
Heterogeneity: I^2 = 75.5%, τ^2 = 0.6325, p < 0.0001
Delhi
Rama et. al. (2019) 4 5 0.80 [0.28; 0.99]
Rehani et. al. (2024) 0 7 0.00 [0.00; 0.41]
Tarai et. al. (2022) 5 56 0.09 [0.03; 0.20]
Random effects model 68 0.23 [0.00; 0.99]
Heterogeneity: I^2 = 79.5%, τ^2 = 3.8512, p = 0.0077
Goa
Narvencar et al. (2012) 5 15 0.33 [0.12; 0.62]
Haryana
Aggarwal et al. (2015) 3 29 0.10 [0.02; 0.27]
Jain et al. (2019) 7 44 0.16 [0.07; 0.30]
Random effects model 73 0.14 [0.01; 0.75]
Heterogeneity: I^2 = 0%, τ^2 = 0, p = 0.5017
Himachal Pradesh
Mahajan et. al. (2009) 1 5 0.20 [0.01; 0.72]
Mahajan et. al. (2008) 1 5 0.20 [0.01; 0.72]
Sood et al. (2016) 2 81 0.02 [0.00; 0.09]
Kumar et al. (2015) 0 14 0.00 [0.00; 0.23]
Mahajan et. al. (2006b) 3 21 0.14 [0.03; 0.36]
Mahajan et. al. (2006a) 3 21 0.14 [0.03; 0.36]
Sharma et al. (2005) 15 45 0.33 [0.20; 0.49]
Mokta et. al. (2017) 21 103 0.20 [0.13; 0.29]
Kumar et. al. (2004) 19 113 0.17 [0.10; 0.25]
Vikrant et al. (2013) 28 515 0.05 [0.04; 0.08]
Rana et. al. (2017) 0 323 0.00 [0.00; 0.01]
Mahajan et. al. (2016a) 13 253 0.05 [0.03; 0.09]
Random effects model 1499 0.10 [0.04; 0.20]
Heterogeneity: I^2 = 84.9%, τ^2 = 1.2880, p < 0.0001
Jammu & Kashmir
Vaz & Gupta (2006) 0 12 0.00 [0.00; 0.26]

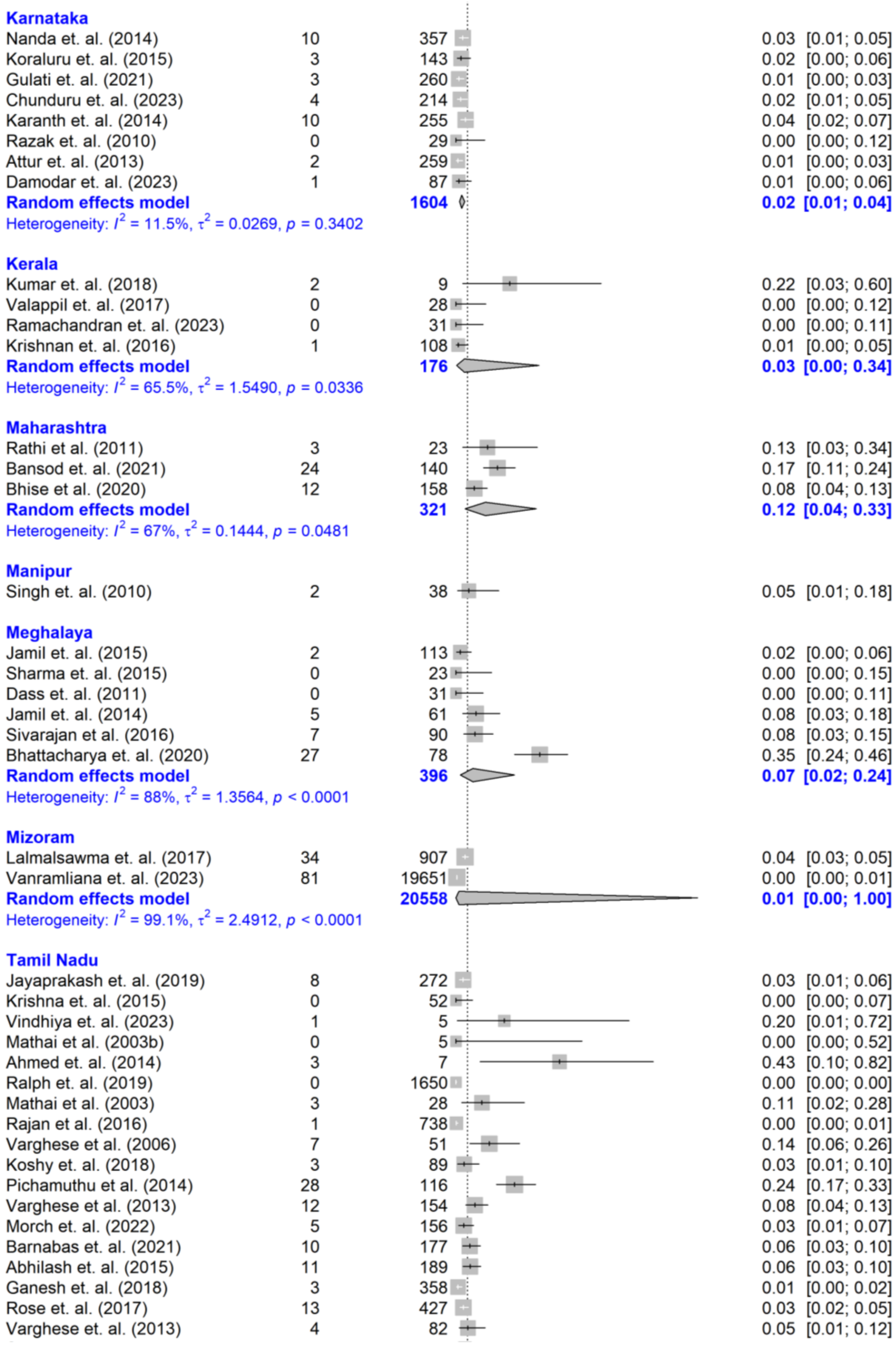
Karnataka
Nanda et. al. (2014) 10 357 0.03 [0.01; 0.05]
Koraluru et. al. (2015) 3 143 0.02 [0.00; 0.06]
Gulati et. al. (2021) 3 260 0.01 [0.00; 0.03]
Chunduru et. al. (2023) 4 214 0.02 [0.01; 0.05]
Karanth et. al. (2014) 10 255 0.04 [0.02; 0.07]
Razak et. al. (2010) 0 29 0.00 [0.00; 0.12]
Attur et. al. (2013) 2 259 0.01 [0.00; 0.03]
Damodar et. al. (2023) 1 87 0.01 [0.00; 0.06]
Random effects model 1604 0.02 [0.01; 0.04]
Heterogeneity: I^2 = 11.5%, τ^2 = 0.0269, p = 0.3402
Kerala
Kumar et. al. (2018) 2 9 0.22 [0.03; 0.60]
Valappil et. al. (2017) 0 28 0.00 [0.00; 0.12]
Ramachandran et. al. (2023) 0 31 0.00 [0.00; 0.11]
Krishnan et. al. (2016) 1 108 0.01 [0.00; 0.05]
Random effects model 176 0.03 [0.00; 0.34]
Heterogeneity: I^2 = 65.5%, τ^2 = 1.5490, p = 0.0336
Maharashtra
Rathi et al. (2011) 3 23 0.13 [0.03; 0.34]
Bansod et. al. (2021) 24 140 0.17 [0.11; 0.24]
Bhise et al. (2020) 12 158 0.08 [0.04; 0.13]
Random effects model 321 0.12 [0.04; 0.33]
Heterogeneity: I^2 = 67%, τ^2 = 0.1444, p = 0.0481
Manipur
Singh et. al. (2010) 2 38 0.05 [0.01; 0.18]
Meghalaya
Jamil et. al. (2015) 2 113 0.02 [0.00; 0.06]
Sharma et. al. (2015) 0 23 0.00 [0.00; 0.15]
Dass et. al. (2011) 0 31 0.00 [0.00; 0.11]
Jamil et. al. (2014) 5 61 0.08 [0.03; 0.18]
Sivarajan et al. (2016) 7 90 0.08 [0.03; 0.15]
Bhattacharya et. al. (2020) 27 78 0.35 [0.24; 0.46]
Random effects model 396 0.07 [0.02; 0.24]
Heterogeneity: I^2 = 88%, τ^2 = 1.3564, p < 0.0001
Mizoram
Lalmalsawma et. al. (2017) 34 907 0.04 [0.03; 0.05]
Vanramliana et. al. (2023) 81 19651 0.00 [0.00; 0.01]
Random effects model 20558 0.01 [0.00; 1.00]
Heterogeneity: I^2 = 99.1%, τ^2 = 2.4912, p < 0.0001
Tamil Nadu
Jayaprakash et. al. (2019) 8 272 0.03 [0.01; 0.06]
Krishna et. al. (2015) 0 52 0.00 [0.00; 0.07]
Vindhiya et. al. (2023) 1 5 0.20 [0.01; 0.72]
Mathai et al. (2003b) 0 5 0.00 [0.00; 0.52]
Ahmed et. al. (2014) 3 7 0.43 [0.10; 0.82]
Ralph et. al. (2019) 0 1650 0.00 [0.00; 0.00]
Mathai et al. (2003) 3 28 0.11 [0.02; 0.28]
Rajan et al. (2016) 1 738 0.00 [0.00; 0.01]
Varghese et al. (2006) 7 51 0.14 [0.06; 0.26]
Koshy et. al. (2018) 3 89 0.03 [0.01; 0.10]
Pichamuthu et al. (2014) 28 116 0.24 [0.17; 0.33]
Varghese et al. (2013) 12 154 0.08 [0.04; 0.13]
Morch et. al. (2022) 5 156 0.03 [0.01; 0.07]
Barnabas et. al. (2021) 10 177 0.06 [0.03; 0.10]
Abhilash et. al. (2015) 11 189 0.06 [0.03; 0.10]
Ganesh et. al. (2018) 3 358 0.01 [0.00; 0.02]
Rose et. al. (2017) 13 427 0.03 [0.02; 0.05]
Varghese et. al. (2013) 4 82 0.05 [0.01; 0.12]

Somashekar et al. (2006)	1	27	0.04 [0.00; 0.19]
Sivathanu et. al. (2017)	0	31	0.00 [0.00; 0.11]
Sivaprakasam et. al. (2020)	3	235	0.01 [0.00; 0.04]
Prakash et. al. (2006)	6	46	0.13 [0.05; 0.26]
Kakarlapudi et. al. (2018)	0	48	0.00 [0.00; 0.07]
Muthukrishnan et. al. (2020)	4	120	0.03 [0.01; 0.08]
Chrispal et al. (2010)	23	189	0.12 [0.08; 0.18]
John et. al. (2023)	7	224	0.03 [0.01; 0.06]
Loganathan et. al. (2021)	13	431	0.03 [0.02; 0.05]
Varghese et. al. (2014)	56	623	0.09 [0.07; 0.12]
Peter et. al. (2013)	20	116	0.17 [0.11; 0.25]
**Random effects model**		**6646**	**0.05 [0.03; 0.08]**
Heterogeneity: $I^2 = 85.1\%$, $\tau^2 = 1.6017$, $p < 0.0001$			
**Uttarakhand**			
Bhat et. al. (2014a)	5	66	0.08 [0.03; 0.17]
Bhat et. al. (2016)	7	96	0.07 [0.03; 0.14]
Ahmad et. al. (2010)	1	9	0.11 [0.00; 0.48]
Ahmad et al. (2016)	6	105	0.06 [0.02; 0.12]
Bhargava et. al. (2016)	24	284	0.08 [0.05; 0.12]
Bhat et. al. (2014b)	4	62	0.06 [0.02; 0.16]
Rawat et al. (2018)	5	168	0.03 [0.01; 0.07]
**Random effects model**		**790**	**0.07 [0.05; 0.09]**
Heterogeneity: $I^2 = 0\%$, $\tau^2 = 0$, $p = 0.4947$			
**Odisha**			
Karthika et. al. (2021)	0	124	0.00 [0.00; 0.03]
Behara et. al. (2021)	0	374	0.00 [0.00; 0.01]
Bal et al. (2021)	9	45	0.20 [0.10; 0.35]
Dhar et. al. (2018)	2	65	0.03 [0.00; 0.11]
Behera et al. (2021)	0	76	0.00 [0.00; 0.05]
Behera et al. (2019)	0	114	0.00 [0.00; 0.03]
Bal et al. (2019)	0	201	0.00 [0.00; 0.02]
Agrawal et. al. (2022)	2	218	0.01 [0.00; 0.03]
**Random effects model**		**1217**	**0.01 [0.00; 0.05]**
Heterogeneity: $I^2 = 84.1\%$, $\tau^2 = 2.0675$, $p < 0.0001$			
**Pondicherry**			
Poomalar & Rekha (2014)	1	340	0.00 [0.00; 0.02]
Vivekanandan et. al. (2010)	1	50	0.02 [0.00; 0.11]
Viswanathan et. al. (2013)	0	65	0.00 [0.00; 0.06]
Narayanasamy et al. (2016)	0	117	0.00 [0.00; 0.03]
Narayanasamy et. al (2018)	0	230	0.00 [0.00; 0.02]
Kumar et al. (2018a)	0	270	0.00 [0.00; 0.01]
Stephen et. al. (2013)	0	28	0.00 [0.00; 0.12]
Basheer et. al. (2016)	0	14	0.00 [0.00; 0.23]
Veerappan et. al. (2021)	0	165	0.00 [0.00; 0.02]
Stephen et. al. (2018)	0	156	0.00 [0.00; 0.02]
Kumar et al. (2012)	1	35	0.03 [0.00; 0.15]
Baidya et. al. (2022)	0	31	0.00 [0.00; 0.11]
**Random effects model**		**1501**	**0.01 [0.00; 0.02]**
Heterogeneity: $I^2 = 0\%$, $\tau^2 = 0$, $p = 0.7064$			
**Punjab**			
Oberoi & Varghese (2014)	3	98	0.03 [0.01; 0.09]
Loomba et. al. (2014)	3	62	0.05 [0.01; 0.13]
**Random effects model**		**160**	**0.04 [0.00; 0.45]**
Heterogeneity: $I^2 = 0\%$, $\tau^2 = 0$, $p = 0.5675$			
**Rajasthan**			
Sinha et al. (2014)	7	42	0.17 [0.07; 0.31]
Sardana et. al. (2020)	1	5	0.20 [0.01; 0.72]
Masand et. al. (2016)	2	30	0.07 [0.01; 0.22]
Takhar et al. (2017)	14	66	0.21 [0.12; 0.33]
Bithu et. al. (2014)	13	133	0.10 [0.05; 0.16]
Dave et al. (2022)	30	1340	0.02 [0.02; 0.03]
**Random effects model**		**1616**	**0.10 [0.04; 0.23]**
Heterogeneity: $I^2 = 92.4\%$, $\tau^2 = 0.7423$, $p < 0.0001$			

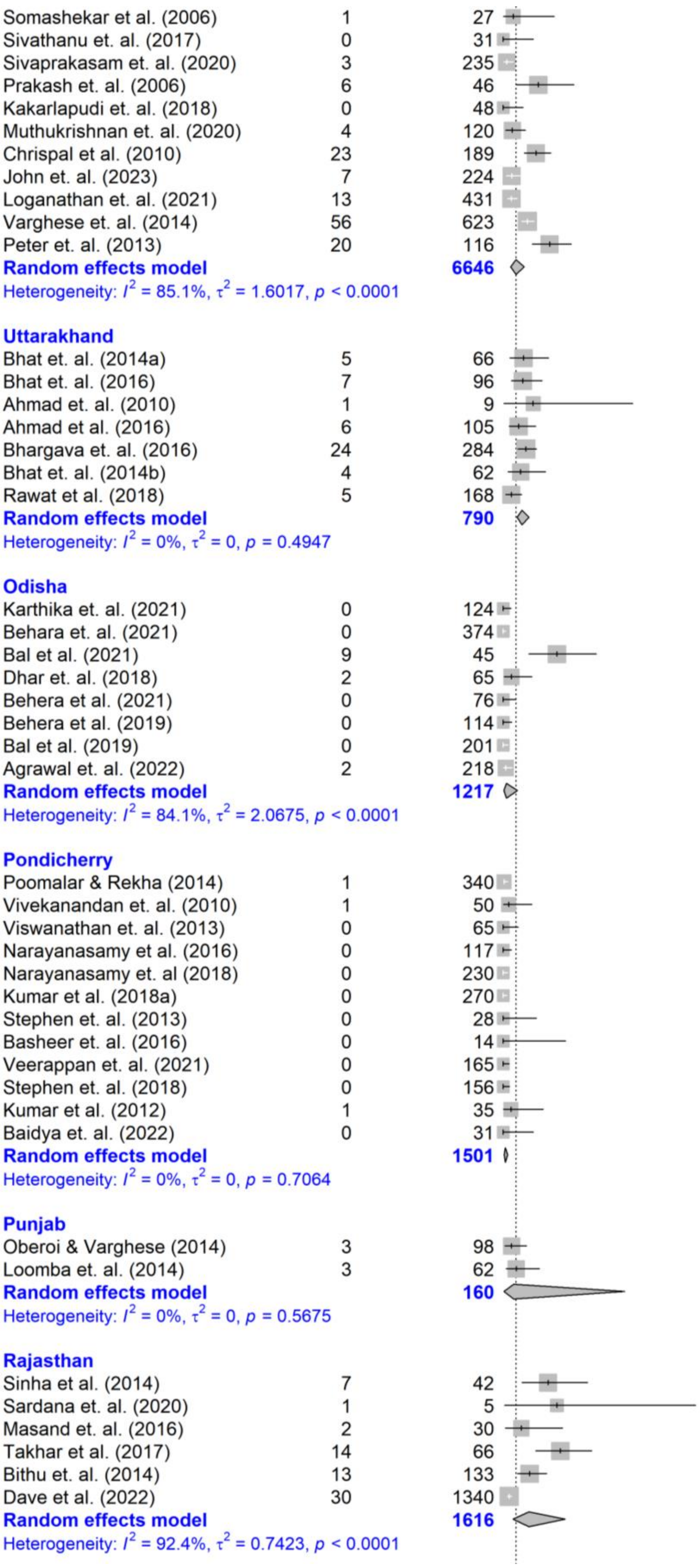

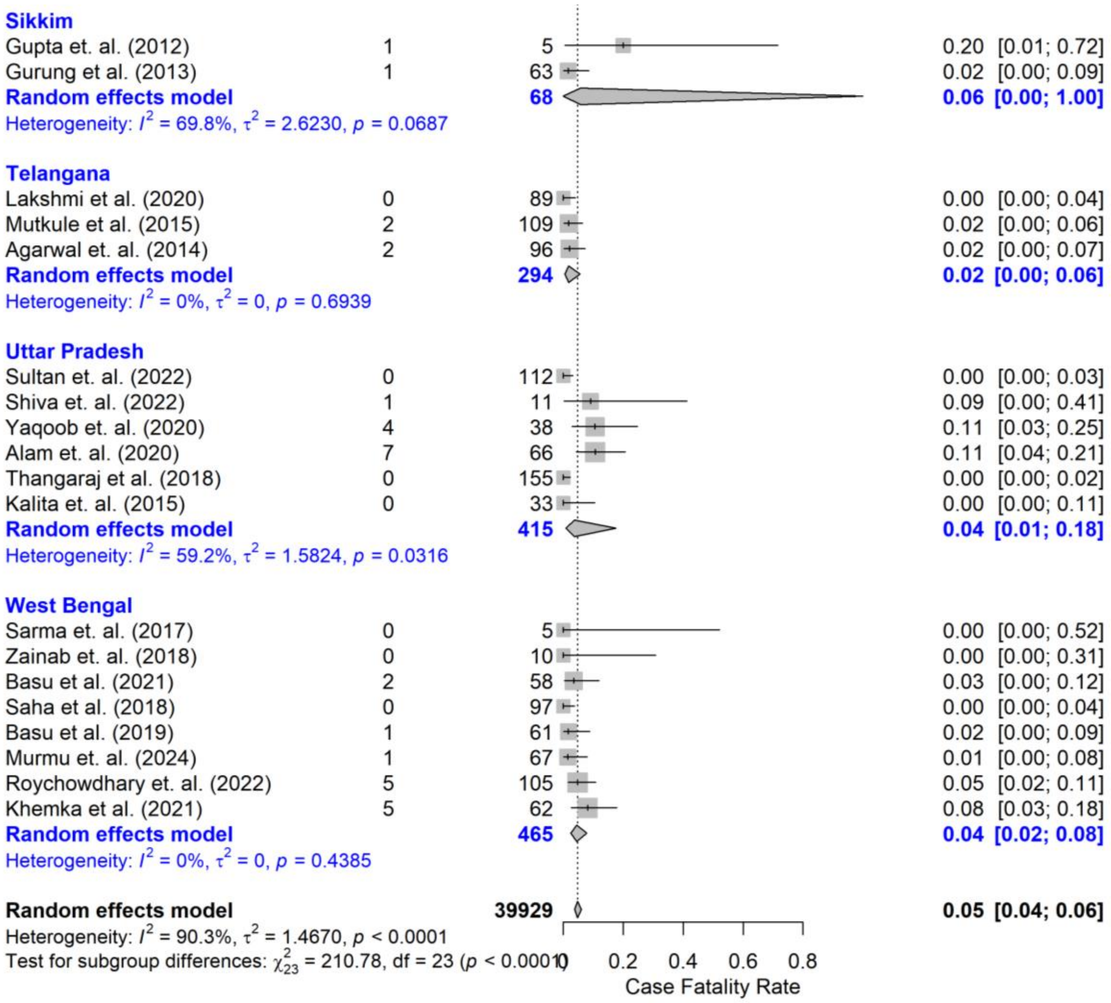

Forest plot 2. Pooled estimates of case fatality ratio of scrub typhus cases in different states of India using a random effects model

Study	No. with symtom	Total Patients	Prevalence	95% CI
**clincal_manifestation = Fever**				
Mathai et al. (2003)	27	28	0.96	[0.82; 1.00]
Mathai et al. (2003b)	2	5	0.40	[0.05; 0.85]
Isaac et al. (2004)	1	8	0.12	[0.00; 0.53]
Mahajan et. al. (2006a)	21	21	1.00	[0.84; 1.00]
Varghese et al. (2006)	50	51	0.98	[0.90; 1.00]
Vivekanandan et. al. (2010)	50	50	1.00	[0.93; 1.00]
Ahmad et. al. (2010)	9	9	1.00	[0.66; 1.00]
Razak et. al. (2010)	29	29	1.00	[0.88; 1.00]
Mahajan et. al. (2010)	4	4	1.00	[0.40; 1.00]
Vaz & Gupta (2006)	12	12	1.00	[0.74; 1.00]
Dass et. al. (2011)	24	24	1.00	[0.86; 1.00]
Rathi et al. (2011)	13	23	0.57	[0.34; 0.77]
Narvencar et al. (2012)	15	15	1.00	[0.78; 1.00]
Kumar et al. (2012)	35	35	1.00	[0.90; 1.00]
Khan et. al. (2012)	8	18	0.44	[0.22; 0.69]
Gupta et. al. (2012)	5	5	1.00	[0.48; 1.00]
Vikrant et al. (2013)	174	174	1.00	[0.98; 1.00]
Viswanathan et. al. (2013)	65	65	1.00	[0.94; 1.00]
Gurung et al. (2013)	37	63	0.59	[0.46; 0.71]
Sinha et al. (2014)	42	42	1.00	[0.92; 1.00]
Loomba et. al. (2014)	31	62	0.50	[0.37; 0.63]
Sethi et. al. (2014)	45	45	1.00	[0.92; 1.00]
Bhat et. al. (2014a)	66	66	1.00	[0.95; 1.00]
Kumar et al. (2014)	48	49	0.98	[0.89; 1.00]
Abhilash et. al. (2015)	189	189	1.00	[0.98; 1.00]
Stephen et al. (2015)	28	28	1.00	[0.88; 1.00]
Kumar et al. (2015)	14	14	1.00	[0.77; 1.00]
Sharma et. al. (2015)	23	23	1.00	[0.85; 1.00]
Ramyasree et al. (2015)	39	39	1.00	[0.91; 1.00]
Kalita et. al. (2016)	10	10	1.00	[0.69; 1.00]
Narayanasamy et al. (2016)	117	117	1.00	[0.97; 1.00]
Rajan et al. (2016)	7	33	0.21	[0.09; 0.39]
Sivarajan et al. (2016)	75	90	0.83	[0.74; 0.90]
Bhargava et. al. (2016)	284	284	1.00	[0.99; 1.00]
Farhana et al. (2016)	37	37	1.00	[0.91; 1.00]
Mahajan et. al. (2016a)	228	253	0.90	[0.86; 0.94]
Basheer et. al. (2016)	6	6	1.00	[0.54; 1.00]
Sharma et. al. (2016)	194	228	0.85	[0.80; 0.89]
Sood et al. (2016)	6	6	1.00	[0.54; 1.00]
Anithraj et. al. (2016)	140	140	1.00	[0.97; 1.00]
Meena et. al. (2015)	6	6	1.00	[0.54; 1.00]
Takhar et al. (2017)	66	66	1.00	[0.95; 1.00]
Sarma et. al. (2017)	5	5	1.00	[0.48; 1.00]
Mokta et. al. (2017)	101	103	0.98	[0.93; 1.00]
Rana et. al. (2017)	37	37	1.00	[0.91; 1.00]
Ganesh et. al. (2018)	358	358	1.00	[0.99; 1.00]
Rawat et al. (2018)	168	168	1.00	[0.98; 1.00]
Kumar et al. (2018a)	14	14	1.00	[0.77; 1.00]
Bal et al. (2019)	70	201	0.35	[0.28; 0.42]
Jain et al. (2019)	39	39	1.00	[0.91; 1.00]
Vikram et al. (2020)	35	35	1.00	[0.90; 1.00]
Bhise et al. (2020)	158	158	1.00	[0.98; 1.00]
Singla et. al. (2020)	13	77	0.17	[0.09; 0.27]
Bhattacharya et. al. (2020)	78	78	1.00	[0.95; 1.00]
Lakshmi et al. (2020)	29	89	0.33	[0.23; 0.43]
Alam et. al. (2020)	66	66	1.00	[0.95; 1.00]
Kispotta et al. (2020)	262	262	1.00	[0.99; 1.00]
Kaur et. al. (2020)	8	8	1.00	[0.63; 1.00]
Bal et al. (2021)	39	45	0.87	[0.73; 0.95]
Williams et al. (2021)	72	72	1.00	[0.95; 1.00]
Verma et. al. (2021)	52	52	1.00	[0.93; 1.00]
Das et. al. (2021)	92	92	1.00	[0.96; 1.00]
Khan et. al. (2017)	104	104	1.00	[0.97; 1.00]
Basu et al. (2021)	18	18	1.00	[0.81; 1.00]
Behara et. al. (2021)	34	34	1.00	[0.90; 1.00]
Paulraj et al. (2021)	13	13	1.00	[0.75; 1.00]
Khemka et al. (2021)	62	62	1.00	[0.94; 1.00]
Bansod et. al. (2021)	140	140	1.00	[0.97; 1.00]
Agrawal et. al. (2022)	209	209	1.00	[0.98; 1.00]
Dave et al. (2022)	170	173	0.98	[0.95; 1.00]
Husain et. al. (2022)	70	361	0.19	[0.15; 0.24]
John et. al. (2023)	198	198	1.00	[0.98; 1.00]

Yadav et al. (2023)	27	27	1.00	[0.87; 1.00]
Damodar et. al. (2023)	87	87	1.00	[0.96; 1.00]
Jana et. al. (2023)	100	206	0.49	[0.42; 0.56]
Narayanasamy et. al (2023)	384	560	0.69	[0.65; 0.72]
Mahajan et. al. (2006b)	21	21	1.00	[0.84; 1.00]
Mahajan et. al. (2008)	5	5	1.00	[0.48; 1.00]
Mahajan et. al. (2009)	5	5	1.00	[0.48; 1.00]
Palanivel et. al. (2012)	67	67	1.00	[0.95; 1.00]
Mittal et al. (2012)	16	90	0.18	[0.11; 0.27]
Stephen et. al. (2013)	28	28	1.00	[0.88; 1.00]
Peter et. al. (2013)	84	84	1.00	[0.96; 1.00]
Varghese et. al. (2014)	620	623	1.00	[0.99; 1.00]
Oberoi & Varghese (2014)	98	98	1.00	[0.96; 1.00]
Kalita et. al. (2015)	33	33	1.00	[0.89; 1.00]
Ramasamy et. al. (2023)	49	49	1.00	[0.93; 1.00]
Bharathi et. al. (2023)	28	28	1.00	[0.88; 1.00]
Vindhiya et. al. (2023)	5	5	1.00	[0.48; 1.00]
Ramachandran et. al. (2023)	31	31	1.00	[0.89; 1.00]
Shiva et. al. (2022)	10	11	0.91	[0.59; 1.00]
Alam et. al. (2022)	47	50	0.94	[0.83; 0.99]
Sardana et. al. (2020)	4	5	0.80	[0.28; 0.99]
Rama et. al. (2019)	5	5	1.00	[0.48; 1.00]
Chunduru et. al. (2023)	20	214	0.09	[0.06; 0.14]
Vikas et. al. (2023)	8	8	1.00	[0.63; 1.00]
Baidya et. al. (2022)	31	31	1.00	[0.89; 1.00]
Loganathan et. al. (2021)	431	431	1.00	[0.99; 1.00]
Kumar et. al. (2021)	125	126	0.99	[0.96; 1.00]
Muthukrishnan et. al. (2020)	84	84	1.00	[0.96; 1.00]
Nallasamy et. al. (2020)	160	160	1.00	[0.98; 1.00]
Sivaprakasam et. al. (2020)	38	39	0.97	[0.87; 1.00]
Jayaprakash et. al. (2019)	193	193	1.00	[0.98; 1.00]
Basu et al. (2019)	61	61	1.00	[0.94; 1.00]
Behera et al. (2019)	114	114	1.00	[0.97; 1.00]
Ralph et. al. (2019)	18	18	1.00	[0.81; 1.00]
Stephen et. al. (2018)	156	156	1.00	[0.98; 1.00]
Biswal et. al. (2018)	8	8	1.00	[0.63; 1.00]
Dhar et. al. (2018)	65	65	1.00	[0.94; 1.00]
Kakarlapudi et. al. (2018)	48	48	1.00	[0.93; 1.00]
Kalal et. al. (2016)	53	53	1.00	[0.93; 1.00]
Bhat et. al. (2016)	96	96	1.00	[0.96; 1.00]
Karanth et. al. (2014)	220	255	0.86	[0.81; 0.90]
Aggarwal et al. (2015)	23	25	0.92	[0.74; 0.99]
Pichamuthu et al. (2014)	112	116	0.97	[0.91; 0.99]
Ahmed et. al. (2014)	7	7	1.00	[0.59; 1.00]
Zainab et. al. (2018)	10	10	1.00	[0.69; 1.00]
Kumar et al. (2018b)	45	50	0.90	[0.78; 0.97]
Valappil et. al. (2017)	28	28	1.00	[0.88; 1.00]
Sivathanu et. al. (2017)	31	31	1.00	[0.89; 1.00]
Balasubramanian et. al. (2016)	50	50	1.00	[0.93; 1.00]
Masand et. al. (2016)	30	30	1.00	[0.88; 1.00]
Misra et. al. (2014)	37	37	1.00	[0.91; 1.00]
Mutkule et al. (2015)	56	58	0.97	[0.88; 1.00]
Bhat et. al. (2014b)	62	62	1.00	[0.94; 1.00]
Jamil et. al. (2014)	59	59	1.00	[0.94; 1.00]
Bhat et. al. (2016)	96	96	1.00	[0.96; 1.00]
Sugumar et. al. (2020)	24	24	1.00	[0.86; 1.00]
Rose et. al. (2017)	427	427	1.00	[0.99; 1.00]
Koshy et. al. (2018)	89	89	1.00	[0.96; 1.00]
Ganesh et. al. (2018)	358	358	1.00	[0.99; 1.00]
Narayanasamy et. al (2018)	230	230	1.00	[0.98; 1.00]
Basu et al. (2019)	61	61	1.00	[0.94; 1.00]
Anitharaj et. al. (2020)	7	140	0.05	[0.02; 0.10]
Barnabas et. al. (2021)	177	177	1.00	[0.98; 1.00]
Basu et al. (2021)	18	18	1.00	[0.81; 1.00]
Veerappan et. al. (2021)	138	155	0.89	[0.83; 0.93]
Narayanasamy et. al (2021)	256	256	1.00	[0.99; 1.00]
Roychowdhary et. al. (2022)	105	105	1.00	[0.97; 1.00]
Karthika et. al. (2021)	124	124	1.00	[0.97; 1.00]
Varghese et. al. (2023)	794	794	1.00	[1.00; 1.00]
Govindaraj et. al. (2024)	36	36	1.00	[0.90; 1.00]
Rehani et. al. (2024)	7	7	1.00	[0.59; 1.00]
**Random effects model**		**13681**	**0.97**	**[0.95; 0.98]**

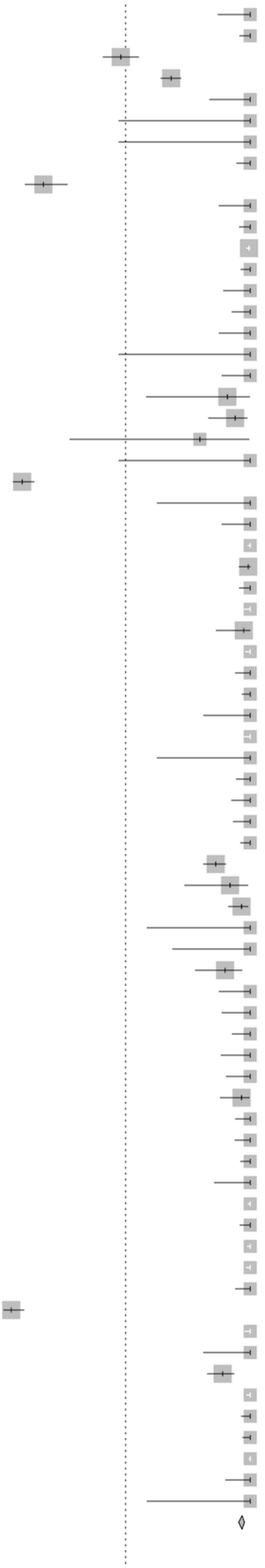

Heterogeneity: I^2 = 93%, τ^2 = 3.4390, p = 0

clincal_manifestation = Myalgia/Arthralgia

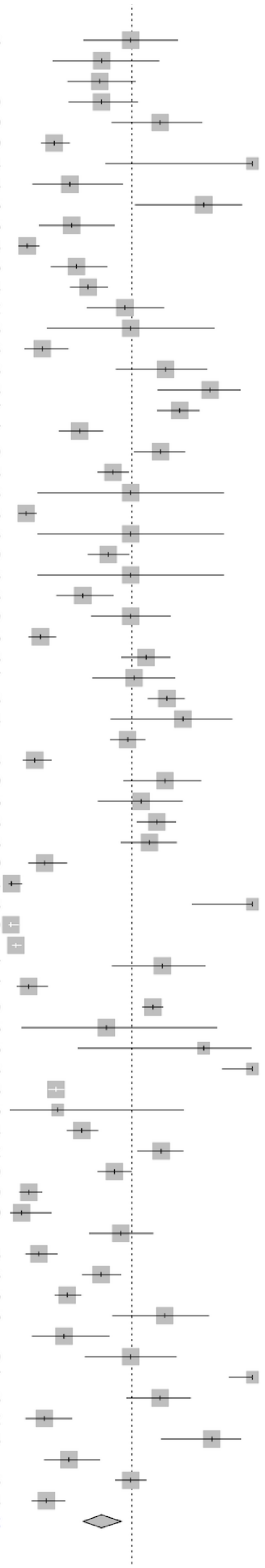

Mathai et al. (2003)	14	28	0.50	[0.31; 0.69]
Mahajan et. al. (2006a)	8	21	0.38	[0.18; 0.62]
Varghese et al. (2006)	19	51	0.37	[0.24; 0.52]
Vivekanandan et. al. (2010)	19	50	0.38	[0.25; 0.53]
Razak et. al. (2010)	18	29	0.62	[0.42; 0.79]
Chrispal et. al. (2010)	35	189	0.19	[0.13; 0.25]
Mahajan et. al. (2010)	4	4	1.00	[0.40; 1.00]
Dass et. al. (2011)	6	24	0.25	[0.10; 0.47]
Narvencar et al. (2012)	12	15	0.80	[0.52; 0.96]
Kumar et al. (2012)	9	35	0.26	[0.12; 0.43]
Vikrant et al. (2013)	13	174	0.07	[0.04; 0.12]
Viswanathan et. al. (2013)	18	65	0.28	[0.17; 0.40]
Varghese et al. (2013)	50	154	0.32	[0.25; 0.40]
Sinha et al. (2014)	20	42	0.48	[0.32; 0.64]
Poomalar & Rekha (2014)	4	8	0.50	[0.16; 0.84]
Bhat et. al. (2014a)	9	66	0.14	[0.06; 0.24]
Stephen et al. (2015)	18	28	0.64	[0.44; 0.81]
Sharma et. al. (2015)	19	23	0.83	[0.61; 0.95]
Narayanasamy et al. (2016)	82	117	0.70	[0.61; 0.78]
Arun Babu et al. (2017)	31	107	0.29	[0.21; 0.39]
Sivarajan et al. (2016)	56	90	0.62	[0.51; 0.72]
Mahajan et. al. (2016a)	108	253	0.43	[0.37; 0.49]
Basheer et. al. (2016)	3	6	0.50	[0.12; 0.88]
Sharma et. al. (2016)	16	228	0.07	[0.04; 0.11]
Sood et al. (2016)	3	6	0.50	[0.12; 0.88]
Anithraj et. al. (2016)	57	140	0.41	[0.32; 0.49]
Meena et. al. (2015)	3	6	0.50	[0.12; 0.88]
Takhar et al. (2017)	20	66	0.30	[0.20; 0.43]
Thangaraj et al. (2017)	20	40	0.50	[0.34; 0.66]
Thangaraj et al. (2018)	20	155	0.13	[0.08; 0.19]
Mokta et. al. (2017)	58	103	0.56	[0.46; 0.66]
Rana et. al. (2017)	19	37	0.51	[0.34; 0.68]
Rawat et al. (2018)	109	168	0.65	[0.57; 0.72]
Kumar et al. (2018a)	10	14	0.71	[0.42; 0.92]
Bal et al. (2019)	98	201	0.49	[0.42; 0.56]
Gaba et al. (2019)	13	123	0.11	[0.06; 0.17]
Jain et al. (2019)	25	39	0.64	[0.47; 0.79]
Vikram et al. (2020)	19	35	0.54	[0.37; 0.71]
Bhise et al. (2020)	96	158	0.61	[0.53; 0.68]
Bhattacharya et. al. (2020)	45	78	0.58	[0.46; 0.69]
Lakshmi et al. (2020)	13	89	0.15	[0.08; 0.24]
Khan et. al. (2017)	1	104	0.01	[0.00; 0.05]
Paulraj et al. (2021)	13	13	1.00	[0.75; 1.00]
Bansod et. al. (2021)	1	140	0.01	[0.00; 0.04]
Husain et. al. (2022)	10	361	0.03	[0.01; 0.05]
Yadav et al. (2023)	17	27	0.63	[0.42; 0.81]
Damodar et. al. (2023)	7	87	0.08	[0.03; 0.16]
Narayanasamy et. al (2023)	331	560	0.59	[0.55; 0.63]
Mahajan et. al. (2008)	2	5	0.40	[0.05; 0.85]
Mahajan et. al. (2009)	4	5	0.80	[0.28; 0.99]
Stephen et. al. (2013)	28	28	1.00	[0.88; 1.00]
Varghese et. al. (2014)	120	623	0.19	[0.16; 0.23]
Rama et. al. (2019)	1	5	0.20	[0.01; 0.72]
Chunduru et. al. (2023)	64	214	0.30	[0.24; 0.37]
Sultan et. al. (2022)	70	112	0.62	[0.53; 0.71]
Thakur et. al. (2019)	91	210	0.43	[0.37; 0.50]
Nallasamy et. al. (2020)	13	160	0.08	[0.04; 0.13]
Sivaprakasam et. al. (2020)	2	39	0.05	[0.01; 0.17]
Basu et al. (2019)	28	61	0.46	[0.33; 0.59]
Behera et al. (2019)	14	114	0.12	[0.07; 0.20]
Stephen et. al. (2018)	59	156	0.38	[0.30; 0.46]
Karanth et. al. (2014)	61	255	0.24	[0.19; 0.30]
Aggarwal et al. (2015)	16	25	0.64	[0.43; 0.82]
Sivathanu et. al. (2017)	7	31	0.23	[0.10; 0.41]
Masand et. al. (2016)	15	30	0.50	[0.31; 0.69]
Misra et. al. (2014)	37	37	1.00	[0.91; 1.00]
Mutkule et al. (2015)	36	58	0.62	[0.48; 0.74]
Bhat et. al. (2014b)	9	62	0.15	[0.07; 0.26]
Sugumar et. al. (2020)	20	24	0.83	[0.63; 0.95]
Basu et al. (2019)	15	61	0.25	[0.14; 0.37]
Narayanasamy et. al (2021)	128	256	0.50	[0.44; 0.56]
Karthika et. al. (2021)	19	124	0.15	[0.09; 0.23]
**Random effects model**		**7252**	**0.38**	**[0.30; 0.46]**

Heterogeneity: $I^2 = 94\%$, $\tau^2 = 1.8593$, $p < 0.01$

clincal_manifestation = Headache

Mathai et al. (2003)	9	28	0.32	[0.16; 0.52]
Mahajan et. al. (2006a)	8	21	0.38	[0.18; 0.62]
Varghese et al. (2006)	33	51	0.65	[0.50; 0.78]
Somashekar et al. (2006)	12	27	0.44	[0.25; 0.65]
Vivekanandan et. al. (2010)	26	50	0.52	[0.37; 0.66]
Razak et. al. (2010)	15	29	0.52	[0.33; 0.71]
Chrispal et. al. (2010)	79	189	0.42	[0.35; 0.49]
Mahajan et. al. (2010)	3	4	0.75	[0.19; 0.99]
Dass et. al. (2011)	6	24	0.25	[0.10; 0.47]
Thakur et al. (2011)	46	61	0.75	[0.63; 0.86]
Kumar et al. (2012)	4	35	0.11	[0.03; 0.27]
Khan et. al. (2012)	2	18	0.11	[0.01; 0.35]
Gupta et. al. (2012)	1	5	0.20	[0.01; 0.72]
Vikrant et al. (2013)	15	174	0.09	[0.05; 0.14]
Viswanathan et. al. (2013)	39	65	0.60	[0.47; 0.72]
Varghese et al. (2013)	66	154	0.43	[0.35; 0.51]
Gurung et al. (2013)	16	63	0.25	[0.15; 0.38]
Sinha et al. (2014)	16	42	0.38	[0.24; 0.54]
Poomalar & Rekha (2014)	4	8	0.50	[0.16; 0.84]
Sethi et. al. (2014)	17	45	0.38	[0.24; 0.53]
Kumar et al. (2014)	14	49	0.29	[0.17; 0.43]
Abhilash et. al. (2015)	122	189	0.65	[0.57; 0.71]
Stephen et al. (2015)	18	28	0.64	[0.44; 0.81]
Stephen et al. (2015)	5	28	0.18	[0.06; 0.37]
Jamil et. al. (2015)	13	13	1.00	[0.75; 1.00]
Sharma et. al. (2015)	21	23	0.91	[0.72; 0.99]
Narayanasamy et al. (2016)	53	117	0.45	[0.36; 0.55]
Rajan et al. (2016)	11	33	0.33	[0.18; 0.52]
Arun Babu et al. (2017)	36	107	0.34	[0.25; 0.43]
Sivarajan et al. (2016)	24	90	0.27	[0.18; 0.37]
Bhargava et. al. (2016)	104	284	0.37	[0.31; 0.43]
Farhana et al. (2016)	22	37	0.59	[0.42; 0.75]
Mahajan et. al. (2016a)	104	253	0.41	[0.35; 0.47]
Basheer et. al. (2016)	4	6	0.67	[0.22; 0.96]
Ahmad et al. (2016)	10	65	0.15	[0.08; 0.26]
Anithraj et. al. (2016)	68	140	0.49	[0.40; 0.57]
Takhar et al. (2017)	22	66	0.33	[0.22; 0.46]
Sarma et. al. (2017)	5	5	1.00	[0.48; 1.00]
Thangaraj et al. (2017)	15	40	0.38	[0.23; 0.54]
Thangaraj et al. (2018)	54	155	0.35	[0.27; 0.43]
Mokta et. al. (2017)	60	103	0.58	[0.48; 0.68]
Rawat et al. (2018)	40	168	0.24	[0.18; 0.31]
Kumar et al. (2018a)	14	14	1.00	[0.77; 1.00]
Bal et al. (2019)	97	201	0.48	[0.41; 0.55]
Jain et al. (2019)	13	39	0.33	[0.19; 0.50]
Vikram et al. (2020)	20	35	0.57	[0.39; 0.74]
Bhise et al. (2020)	98	158	0.62	[0.54; 0.70]
Bhattacharya et. al. (2020)	41	78	0.53	[0.41; 0.64]
Lakshmi et al. (2020)	9	89	0.10	[0.05; 0.18]
Alam et. al. (2020)	11	66	0.17	[0.09; 0.28]
Kaur et. al. (2020)	2	8	0.25	[0.03; 0.65]
Bal et al. (2021)	16	45	0.36	[0.22; 0.51]
Williams et al. (2021)	14	72	0.19	[0.11; 0.30]
Verma et. al. (2021)	36	52	0.69	[0.55; 0.81]
Das et. al. (2021)	47	92	0.51	[0.40; 0.62]
Khan et. al. (2017)	70	104	0.67	[0.57; 0.76]
Paulraj et al. (2021)	5	13	0.38	[0.14; 0.68]
Bansod et. al. (2021)	11	140	0.08	[0.04; 0.14]
Agrawal et. al. (2022)	12	209	0.06	[0.03; 0.10]
Dave et al. (2022)	9	173	0.05	[0.02; 0.10]
Husain et. al. (2022)	20	361	0.06	[0.03; 0.08]
John et. al. (2023)	30	198	0.15	[0.10; 0.21]
Yadav et al. (2023)	18	27	0.67	[0.46; 0.83]
Damodar et. al. (2023)	32	87	0.37	[0.27; 0.48]
Jana et. al. (2023)	28	206	0.14	[0.09; 0.19]
Mahajan et. al. (2008)	2	5	0.40	[0.05; 0.85]
Mahajan et. al. (2009)	4	5	0.80	[0.28; 0.99]
Chrispal et al. (2010)	79	189	0.42	[0.35; 0.49]
Mittal et al. (2012)	12	90	0.13	[0.07; 0.22]
Stephen et. al. (2013)	28	28	1.00	[0.88; 1.00]
Varghese et. al. (2013)	13	16	0.81	[0.54; 0.96]
Varghese et. al. (2014)	285	623	0.46	[0.42; 0.50]
Boorugu et al. (2014)	79	189	0.42	[0.35; 0.49]

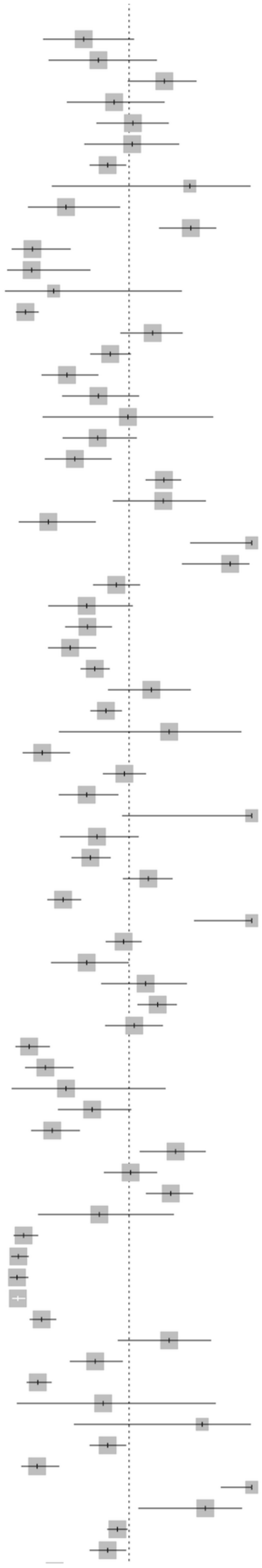

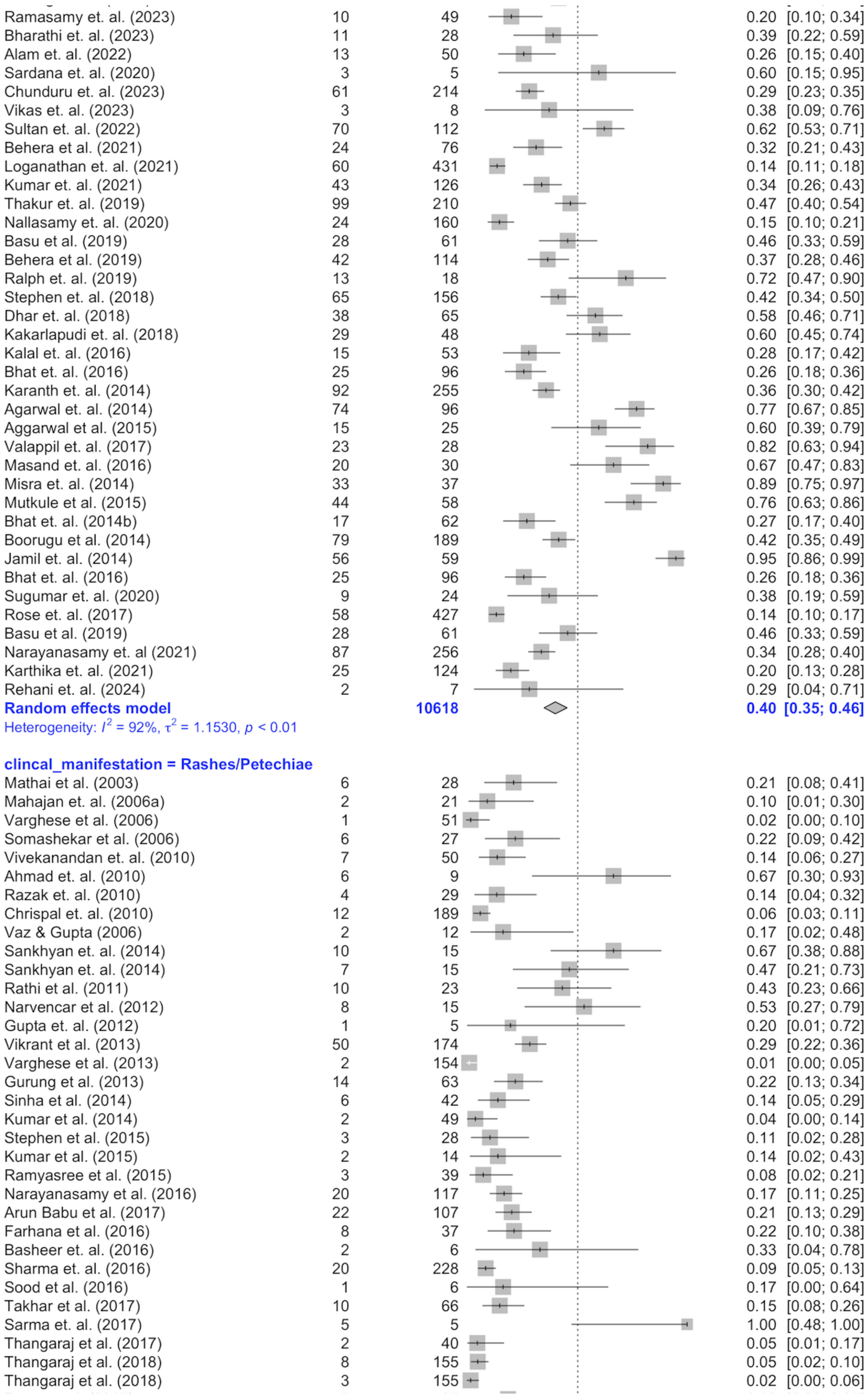
Ramasamy et. al. (2023) 10 49 0.20 [0.10; 0.34]
Bharathi et. al. (2023) 11 28 0.39 [0.22; 0.59]
Alam et. al. (2022) 13 50 0.26 [0.15; 0.40]
Sardana et. al. (2020) 3 5 0.60 [0.15; 0.95]
Chunduru et. al. (2023) 61 214 0.29 [0.23; 0.35]
Vikas et. al. (2023) 3 8 0.38 [0.09; 0.76]
Sultan et. al. (2022) 70 112 0.62 [0.53; 0.71]
Behera et al. (2021) 24 76 0.32 [0.21; 0.43]
Loganathan et. al. (2021) 60 431 0.14 [0.11; 0.18]
Kumar et. al. (2021) 43 126 0.34 [0.26; 0.43]
Thakur et. al. (2019) 99 210 0.47 [0.40; 0.54]
Nallasamy et. al. (2020) 24 160 0.15 [0.10; 0.21]
Basu et al. (2019) 28 61 0.46 [0.33; 0.59]
Behera et al. (2019) 42 114 0.37 [0.28; 0.46]
Ralph et. al. (2019) 13 18 0.72 [0.47; 0.90]
Stephen et. al. (2018) 65 156 0.42 [0.34; 0.50]
Dhar et. al. (2018) 38 65 0.58 [0.46; 0.71]
Kakarlapudi et. al. (2018) 29 48 0.60 [0.45; 0.74]
Kalal et. al. (2016) 15 53 0.28 [0.17; 0.42]
Bhat et. al. (2016) 25 96 0.26 [0.18; 0.36]
Karanth et. al. (2014) 92 255 0.36 [0.30; 0.42]
Agarwal et. al. (2014) 74 96 0.77 [0.67; 0.85]
Aggarwal et al. (2015) 15 25 0.60 [0.39; 0.79]
Valappil et. al. (2017) 23 28 0.82 [0.63; 0.94]
Masand et. al. (2016) 20 30 0.67 [0.47; 0.83]
Misra et. al. (2014) 33 37 0.89 [0.75; 0.97]
Mutkule et al. (2015) 44 58 0.76 [0.63; 0.86]
Bhat et. al. (2014b) 17 62 0.27 [0.17; 0.40]
Boorugu et al. (2014) 79 189 0.42 [0.35; 0.49]
Jamil et. al. (2014) 56 59 0.95 [0.86; 0.99]
Bhat et. al. (2016) 25 96 0.26 [0.18; 0.36]
Sugumar et. al. (2020) 9 24 0.38 [0.19; 0.59]
Rose et. al. (2017) 58 427 0.14 [0.10; 0.17]
Basu et al. (2019) 28 61 0.46 [0.33; 0.59]
Narayanasamy et. al (2021) 87 256 0.34 [0.28; 0.40]
Karthika et. al. (2021) 25 124 0.20 [0.13; 0.28]
Rehani et. al. (2024) 2 7 0.29 [0.04; 0.71]
Random effects model 10618 0.40 [0.35; 0.46]
Heterogeneity: I^2 = 92%, τ^2 = 1.1530, p < 0.01
clincal_manifestation = Rashes/Petechiae
Mathai et al. (2003) 6 28 0.21 [0.08; 0.41]
Mahajan et. al. (2006a) 2 21 0.10 [0.01; 0.30]
Varghese et al. (2006) 1 51 0.02 [0.00; 0.10]
Somashekar et al. (2006) 6 27 0.22 [0.09; 0.42]
Vivekanandan et. al. (2010) 7 50 0.14 [0.06; 0.27]
Ahmad et. al. (2010) 6 9 0.67 [0.30; 0.93]
Razak et. al. (2010) 4 29 0.14 [0.04; 0.32]
Chrispal et. al. (2010) 12 189 0.06 [0.03; 0.11]
Vaz & Gupta (2006) 2 12 0.17 [0.02; 0.48]
Sankhyan et. al. (2014) 10 15 0.67 [0.38; 0.88]
Sankhyan et. al. (2014) 7 15 0.47 [0.21; 0.73]
Rathi et al. (2011) 10 23 0.43 [0.23; 0.66]
Narvencar et al. (2012) 8 15 0.53 [0.27; 0.79]
Gupta et. al. (2012) 1 5 0.20 [0.01; 0.72]
Vikrant et al. (2013) 50 174 0.29 [0.22; 0.36]
Varghese et al. (2013) 2 154 0.01 [0.00; 0.05]
Gurung et al. (2013) 14 63 0.22 [0.13; 0.34]
Sinha et al. (2014) 6 42 0.14 [0.05; 0.29]
Kumar et al. (2014) 2 49 0.04 [0.00; 0.14]
Stephen et al. (2015) 3 28 0.11 [0.02; 0.28]
Kumar et al. (2015) 2 14 0.14 [0.02; 0.43]
Ramyasree et al. (2015) 3 39 0.08 [0.02; 0.21]
Narayanasamy et al. (2016) 20 117 0.17 [0.11; 0.25]
Arun Babu et al. (2017) 22 107 0.21 [0.13; 0.29]
Farhana et al. (2016) 8 37 0.22 [0.10; 0.38]
Basheer et. al. (2016) 2 6 0.33 [0.04; 0.78]
Sharma et. al. (2016) 20 228 0.09 [0.05; 0.13]
Sood et al. (2016) 1 6 0.17 [0.00; 0.64]
Takhar et al. (2017) 10 66 0.15 [0.08; 0.26]
Sarma et. al. (2017) 5 5 1.00 [0.48; 1.00]
Thangaraj et al. (2017) 2 40 0.05 [0.01; 0.17]
Thangaraj et al. (2018) 8 155 0.05 [0.02; 0.10]
Thangaraj et al. (2018) 3 155 0.02 [0.00; 0.06]

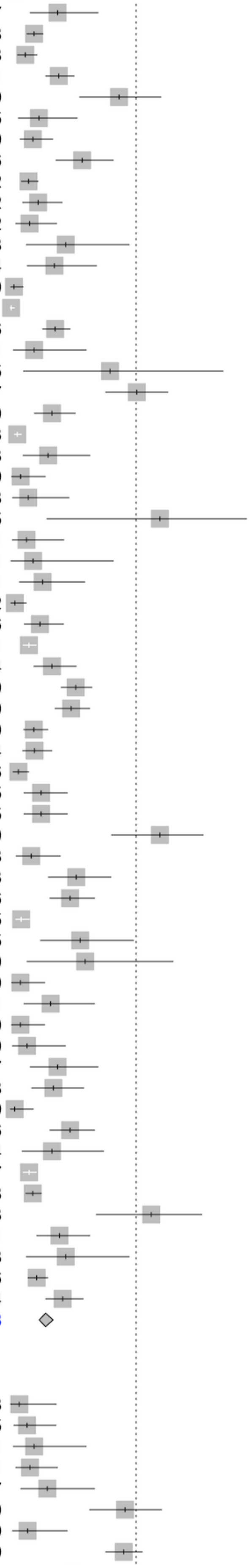

Rana et. al. (2017)	7	37	0.19 [0.08; 0.35]
Ganesh et. al. (2018)	34	358	0.09 [0.07; 0.13]
Rawat et al. (2018)	10	168	0.06 [0.03; 0.11]
Bal et al. (2019)	39	201	0.19 [0.14; 0.26]
Jain et al. (2019)	17	39	0.44 [0.28; 0.60]
Vikram et al. (2020)	4	35	0.11 [0.03; 0.27]
Lakshmi et al. (2020)	8	89	0.09 [0.04; 0.17]
Alam et. al. (2020)	19	66	0.29 [0.18; 0.41]
Kispotta et al. (2020)	19	262	0.07 [0.04; 0.11]
Williams et al. (2021)	8	72	0.11 [0.05; 0.21]
Verma et. al. (2021)	4	52	0.08 [0.02; 0.19]
Basu et al. (2021)	4	18	0.22 [0.06; 0.48]
Behara et. al. (2021)	6	34	0.18 [0.07; 0.35]
Bansod et. al. (2021)	2	140	0.01 [0.00; 0.05]
Husain et. al. (2022)	1	361	0.00 [0.00; 0.02]
Jana et. al. (2023)	37	206	0.18 [0.13; 0.24]
Mahajan et. al. (2006b)	2	21	0.10 [0.01; 0.30]
Mahajan et. al. (2009)	2	5	0.40 [0.05; 0.85]
Palanivel et. al. (2012)	34	67	0.51 [0.38; 0.63]
Mittal et al. (2012)	15	90	0.17 [0.10; 0.26]
Varghese et. al. (2014)	17	623	0.03 [0.02; 0.04]
Kalita et. al. (2015)	5	33	0.15 [0.05; 0.32]
Ramasamy et. al. (2023)	2	49	0.04 [0.00; 0.14]
Bharathi et. al. (2023)	2	28	0.07 [0.01; 0.24]
Vindhiya et. al. (2023)	3	5	0.60 [0.15; 0.95]
Ramachandran et. al. (2023)	2	31	0.06 [0.01; 0.21]
Shiva et. al. (2022)	1	11	0.09 [0.00; 0.41]
Baidya et. al. (2022)	4	31	0.13 [0.04; 0.30]
Sultan et. al. (2022)	2	112	0.02 [0.00; 0.06]
Behera et al. (2021)	9	76	0.12 [0.06; 0.21]
Loganathan et. al. (2021)	32	431	0.07 [0.05; 0.10]
Muthukrishnan et. al. (2020)	14	84	0.17 [0.09; 0.26]
Thakur et. al. (2019)	55	210	0.26 [0.20; 0.33]
Nallasamy et. al. (2020)	39	160	0.24 [0.18; 0.32]
Nallasamy et. al. (2020)	15	160	0.09 [0.05; 0.15]
Behera et al. (2019)	11	114	0.10 [0.05; 0.17]
Stephen et. al. (2018)	5	156	0.03 [0.01; 0.07]
Dhar et. al. (2018)	8	65	0.12 [0.05; 0.23]
Dhar et. al. (2018)	8	65	0.12 [0.05; 0.23]
Saha et al. (2018)	18	30	0.60 [0.41; 0.77]
Kakarlapudi et. al. (2018)	4	48	0.08 [0.02; 0.20]
Kalal et. al. (2016)	14	53	0.26 [0.15; 0.40]
Bhat et. al. (2016)	23	96	0.24 [0.16; 0.34]
Karanth et. al. (2014)	11	255	0.04 [0.02; 0.08]
Aggarwal et al. (2015)	7	25	0.28 [0.12; 0.49]
Zainab et. al. (2018)	3	10	0.30 [0.07; 0.65]
Kumar et al. (2018b)	2	50	0.04 [0.00; 0.14]
Sivathanu et. al. (2017)	5	31	0.16 [0.05; 0.34]
Balasubramanian et. al. (2016)	2	50	0.04 [0.00; 0.14]
Masand et. al. (2016)	2	30	0.07 [0.01; 0.22]
Misra et. al. (2014)	7	37	0.19 [0.08; 0.35]
Mutkule et al. (2015)	10	58	0.17 [0.09; 0.29]
Jamil et. al. (2014)	1	59	0.02 [0.00; 0.09]
Bhat et. al. (2016)	23	96	0.24 [0.16; 0.34]
Sugumar et. al. (2020)	4	24	0.17 [0.05; 0.37]
Rose et. al. (2017)	32	427	0.07 [0.05; 0.10]
Ganesh et. al. (2018)	32	358	0.09 [0.06; 0.12]
Rauf et. al. (2018)	13	23	0.57 [0.34; 0.77]
Basu et al. (2019)	12	61	0.20 [0.11; 0.32]
Basu et al. (2021)	4	18	0.22 [0.06; 0.48]
Narayanasamy et. al (2021)	27	256	0.11 [0.07; 0.15]
Karthika et. al. (2021)	26	124	0.21 [0.14; 0.29]
**Random effects model**		**8958**	**0.14 [0.12; 0.17]**
Heterogeneity: I^2 = 85%, τ^2 = 1.0295, p < 0.01			
**clincal_manifestation = Eschar**			
Mathai et al. (2003)	1	28	0.04 [0.00; 0.18]
Sharma et al. (2005)	3	45	0.07 [0.01; 0.18]
Mahajan et. al. (2006a)	2	21	0.10 [0.01; 0.30]
Varghese et al. (2006)	4	51	0.08 [0.02; 0.19]
Somashekar et al. (2006)	4	27	0.15 [0.04; 0.34]
Vivekanandan et. al. (2010)	23	50	0.46 [0.32; 0.61]
Razak et. al. (2010)	2	29	0.07 [0.01; 0.23]
Chrispal et. al. (2010)	86	189	0.46 [0.38; 0.53]

Mahajan et. al. (2010)	1	4	0.25 [0.01; 0.81]
Sankhyan et. al. (2014)	2	15	0.13 [0.02; 0.40]
Dass et. al. (2011)	10	24	0.42 [0.22; 0.63]
Thakur et al. (2011)	47	61	0.77 [0.65; 0.87]
Rathi et al. (2011)	5	23	0.22 [0.07; 0.44]
Narvencar et al. (2012)	2	15	0.13 [0.02; 0.40]
Kumar et al. (2012)	4	35	0.11 [0.03; 0.27]
Vikrant et al. (2013)	32	174	0.18 [0.13; 0.25]
Viswanathan et. al. (2013)	13	65	0.20 [0.11; 0.32]
Varghese et al. (2013)	86	154	0.56 [0.48; 0.64]
Gurung et al. (2013)	12	63	0.19 [0.10; 0.31]
Loomba et. al. (2014)	9	62	0.15 [0.07; 0.26]
Poomalar & Rekha (2014)	2	8	0.25 [0.03; 0.65]
Sethi et. al. (2014)	2	45	0.04 [0.01; 0.15]
Bhat et. al. (2014a)	13	66	0.20 [0.11; 0.31]
Subbalaxmi et. al. (2014)	23	174	0.13 [0.09; 0.19]
Kumar et al. (2014)	9	49	0.18 [0.09; 0.32]
Abhilash et. al. (2015)	52	189	0.28 [0.21; 0.34]
Stephen et al. (2015)	6	28	0.21 [0.08; 0.41]
Jamil et. al. (2015)	4	13	0.31 [0.09; 0.61]
Krishna et. al. (2015)	35	52	0.67 [0.53; 0.80]
Kumar et al. (2015)	3	14	0.21 [0.05; 0.51]
Kalita et. al. (2016)	5	10	0.50 [0.19; 0.81]
Narayanasamy et al. (2016)	49	117	0.42 [0.33; 0.51]
Rajan et al. (2016)	17	33	0.52 [0.34; 0.69]
Arun Babu et al. (2017)	37	107	0.35 [0.26; 0.44]
Sivarajan et al. (2016)	10	90	0.11 [0.05; 0.19]
Bhargava et. al. (2016)	49	284	0.17 [0.13; 0.22]
Mahajan et. al. (2016a)	112	253	0.44 [0.38; 0.51]
Basheer et. al. (2016)	2	6	0.33 [0.04; 0.78]
Sood et al. (2016)	1	6	0.17 [0.00; 0.64]
Anithraj et. al. (2016)	34	140	0.24 [0.17; 0.32]
Takhar et al. (2017)	8	66	0.12 [0.05; 0.22]
Thangaraj et al. (2017)	2	40	0.05 [0.01; 0.17]
Thangaraj et al. (2018)	2	155	0.01 [0.00; 0.05]
Mokta et. al. (2017)	32	103	0.31 [0.22; 0.41]
Rana et. al. (2017)	15	37	0.41 [0.25; 0.58]
Ganesh et. al. (2018)	241	358	0.67 [0.62; 0.72]
Rawat et al. (2018)	8	168	0.05 [0.02; 0.09]
Kumar et al. (2018a)	6	14	0.43 [0.18; 0.71]
Bal et al. (2019)	36	201	0.18 [0.13; 0.24]
Bhattacharya et. al. (2019)	32	129	0.25 [0.18; 0.33]
Gaba et al. (2019)	40	123	0.33 [0.24; 0.42]
Jain et al. (2019)	5	39	0.13 [0.04; 0.27]
Vikram et al. (2020)	2	35	0.06 [0.01; 0.19]
Jamil et. al. (2019)	9	12	0.75 [0.43; 0.95]
Bhise et al. (2020)	46	158	0.29 [0.22; 0.37]
Bhattacharya et. al. (2020)	31	78	0.40 [0.29; 0.51]
Lakshmi et al. (2020)	4	89	0.04 [0.01; 0.11]
Alam et. al. (2020)	5	66	0.08 [0.03; 0.17]
Kispotta et al. (2020)	83	262	0.32 [0.26; 0.38]
Kaur et. al. (2020)	1	8	0.12 [0.00; 0.53]
Bal et al. (2021)	7	45	0.16 [0.06; 0.29]
Williams et al. (2021)	14	72	0.19 [0.11; 0.30]
Verma et. al. (2021)	11	52	0.21 [0.11; 0.35]
Basu et al. (2021)	6	18	0.33 [0.13; 0.59]
Behara et. al. (2021)	6	34	0.18 [0.07; 0.35]
Paulraj et al. (2021)	1	13	0.08 [0.00; 0.36]
Khemka et al. (2021)	9	62	0.15 [0.07; 0.26]
Bansod et. al. (2021)	33	140	0.24 [0.17; 0.31]
Agrawal et. al. (2022)	57	209	0.27 [0.21; 0.34]
Dave et al. (2022)	67	173	0.39 [0.31; 0.46]
Roychowdhary et. al. (2022)	35	105	0.33 [0.24; 0.43]
Jana et. al. (2023)	41	206	0.20 [0.15; 0.26]
Narayanasamy et. al (2023)	279	560	0.50 [0.46; 0.54]
Mahajan et. al. (2006b)	2	21	0.10 [0.01; 0.30]
Mahajan et. al. (2009)	2	5	0.40 [0.05; 0.85]
Palanivel et. al. (2012)	31	67	0.46 [0.34; 0.59]
Mittal et al. (2012)	6	90	0.07 [0.02; 0.14]
Stephen et. al. (2013)	1	28	0.04 [0.00; 0.18]
Peter et. al. (2013)	80	84	0.95 [0.88; 0.99]
Varghese et. al. (2014)	271	623	0.43 [0.40; 0.47]
Oberoi & Varghese (2014)	10	98	0.10 [0.05; 0.18]
Kalita et. al. (2015)	6	33	0.18 [0.07; 0.35]
[illegible]	[illegible]	[illegible]	[illegible]

Ramasamy et. al. (2023)	13	49	0.27 [0.15; 0.41]
Ramachandran et. al. (2023)	26	31	0.84 [0.66; 0.95]
Chunduru et. al. (2023)	22	214	0.10 [0.07; 0.15]
Baidya et. al. (2022)	19	31	0.61 [0.42; 0.78]
Behera et al. (2021)	4	76	0.05 [0.01; 0.13]
Kumar et. al. (2021)	27	126	0.21 [0.15; 0.30]
Veerappan et. al. (2021)	78	155	0.50 [0.42; 0.58]
Muthukrishnan et. al. (2020)	31	84	0.37 [0.27; 0.48]
Thakur et. al. (2019)	36	210	0.17 [0.12; 0.23]
Nallasamy et. al. (2020)	40	160	0.25 [0.19; 0.32]
Sivaprakasam et. al. (2020)	10	39	0.26 [0.13; 0.42]
Jayaprakash et. al. (2019)	55	193	0.28 [0.22; 0.35]
Behera et al. (2019)	7	114	0.06 [0.03; 0.12]
Ralph et. al. (2019)	8	18	0.44 [0.22; 0.69]
Stephen et. al. (2018)	30	156	0.19 [0.13; 0.26]
Biswal et. al. (2018)	8	8	1.00 [0.63; 1.00]
Dhar et. al. (2018)	15	65	0.23 [0.14; 0.35]
Saha et al. (2018)	6	30	0.20 [0.08; 0.39]
Bhat et. al. (2016)	22	96	0.23 [0.15; 0.33]
Krishnan et. al. (2016)	48	108	0.44 [0.35; 0.54]
Karanth et. al. (2014)	64	255	0.25 [0.20; 0.31]
Agarwal et. al. (2014)	20	96	0.21 [0.13; 0.30]
Aggarwal et al. (2015)	3	25	0.12 [0.03; 0.31]
Nanda et. al. (2014)	70	123	0.57 [0.48; 0.66]
Ahmed et. al. (2014)	3	7	0.43 [0.10; 0.82]
Zainab et. al. (2018)	2	10	0.20 [0.03; 0.56]
Kumar et al. (2018b)	3	50	0.06 [0.01; 0.17]
Valappil et. al. (2017)	7	28	0.25 [0.11; 0.45]
Sivathanu et. al. (2017)	23	31	0.74 [0.55; 0.88]
Balasubramanian et. al. (2016)	3	50	0.06 [0.01; 0.17]
Masand et. al. (2016)	1	30	0.03 [0.00; 0.17]
Misra et. al. (2014)	17	37	0.46 [0.29; 0.63]
Mutkule et al. (2015)	10	58	0.17 [0.09; 0.29]
Jamil et. al. (2014)	16	59	0.27 [0.16; 0.40]
Bhat et. al. (2016)	22	96	0.23 [0.15; 0.33]
Sugumar et. al. (2020)	8	24	0.33 [0.16; 0.55]
Koshy et. al. (2018)	67	89	0.75 [0.65; 0.84]
Ganesh et. al. (2018)	241	358	0.67 [0.62; 0.72]
Basu et al. (2019)	30	61	0.49 [0.36; 0.62]
Anitharaj et. al. (2020)	9	140	0.06 [0.03; 0.12]
Barnabas et. al. (2021)	115	177	0.65 [0.57; 0.72]
Basu et al. (2021)	6	18	0.33 [0.13; 0.59]
Veerappan et. al. (2021)	73	155	0.47 [0.39; 0.55]
Narayanasamy et. al (2021)	132	256	0.52 [0.45; 0.58]
Roychowdhary et. al. (2022)	37	105	0.35 [0.26; 0.45]
Karthika et. al. (2021)	16	124	0.13 [0.08; 0.20]
Rehani et. al. (2024)	2	7	0.29 [0.04; 0.71]
**Random effects model**		**12130**	**0.26 [0.22; 0.30]**
Heterogeneity: $I^2 = 92\%$, $\tau^2 = 1.1118$, $p < 0.01$			
**clincal_manifestation = Chills and Rigor**			
Mathai et al. (2003b)	1	5	0.20 [0.01; 0.72]
Mahajan et. al. (2006a)	15	21	0.71 [0.48; 0.89]
Razak et. al. (2010)	23	29	0.79 [0.60; 0.92]
Mahajan et. al. (2010)	3	4	0.75 [0.19; 0.99]
Viswanathan et. al. (2013)	60	65	0.92 [0.83; 0.97]
Sethi et. al. (2014)	5	45	0.11 [0.04; 0.24]
Stephen et al. (2015)	20	28	0.71 [0.51; 0.87]
Ramyasree et al. (2015)	39	39	1.00 [0.91; 1.00]
Mahajan et. al. (2016a)	172	253	0.68 [0.62; 0.74]
Sharma et. al. (2016)	194	228	0.85 [0.80; 0.89]
Sood et al. (2016)	3	6	0.50 [0.12; 0.88]
Anithraj et. al. (2016)	88	140	0.63 [0.54; 0.71]
Vikram et al. (2020)	16	35	0.46 [0.29; 0.63]
Dave et al. (2022)	65	173	0.38 [0.30; 0.45]
Husain et. al. (2022)	20	361	0.06 [0.03; 0.08]
Mahajan et. al. (2006b)	15	21	0.71 [0.48; 0.89]
Mahajan et. al. (2008)	2	5	0.40 [0.05; 0.85]
Mahajan et. al. (2009)	3	5	0.60 [0.15; 0.95]
Mittal et al. (2012)	16	90	0.18 [0.11; 0.27]
Chunduru et. al. (2023)	78	214	0.36 [0.30; 0.43]
Sultan et. al. (2022)	91	112	0.81 [0.73; 0.88]
Stephen et. al. (2018)	74	156	0.47 [0.39; 0.56]
**Random effects model**		**2035**	**0.56 [0.40; 0.71]**
Heterogeneity: $I^2 = 95\%$, $\tau^2 = 1.9050$, $p < 0.01$			

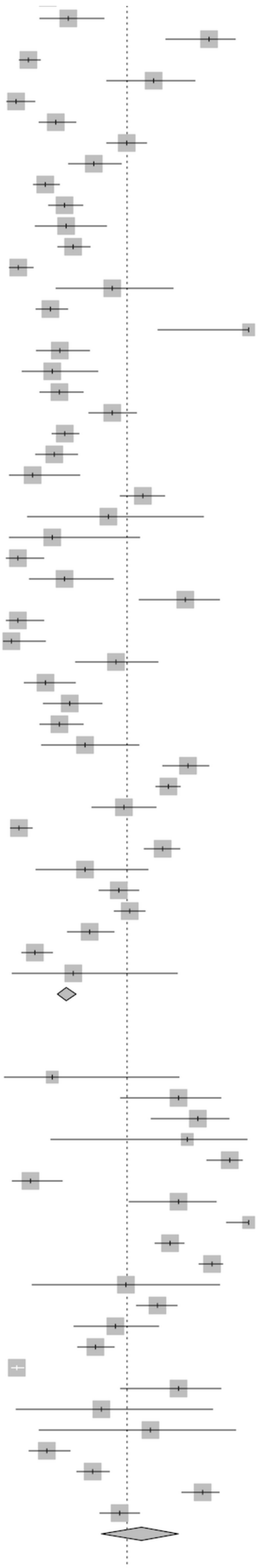

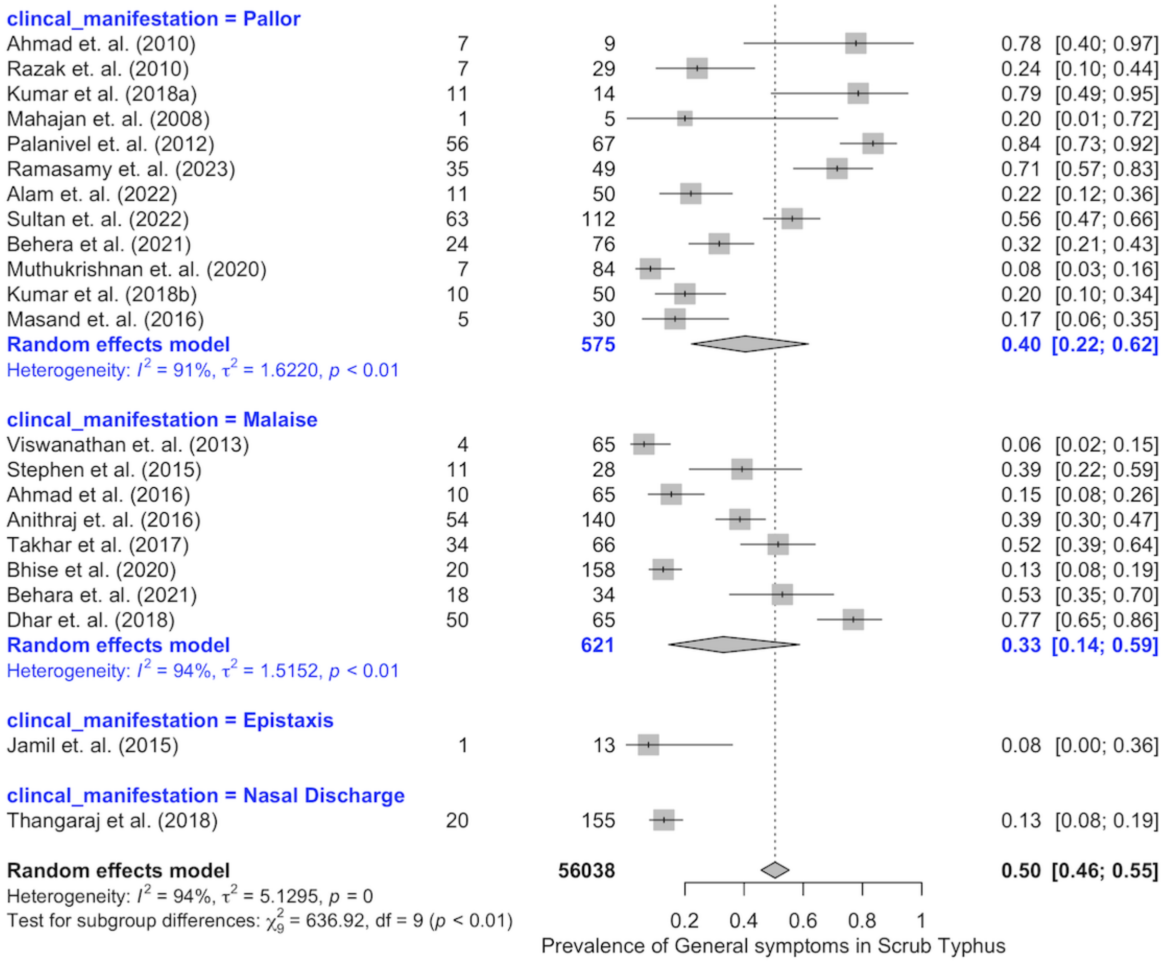

Forest plot 3. Pooled prevalence of different types of general symptoms of scrub typhus

Study	No. with symtom	Total Patients	Prevalence	95% CI
**clincal_manifestation = Nausea**				
Mathai et al. (2003)	13	28	0.46	[0.28; 0.66]
Varghese et al. (2006)	28	51	0.55	[0.40; 0.69]
Somashekar et al. (2006)	12	27	0.44	[0.25; 0.65]
Vivekanandan et. al. (2010)	29	50	0.58	[0.43; 0.72]
Narvencar et al. (2012)	15	15	1.00	[0.78; 1.00]
Khan et. al. (2012)	2	18	0.11	[0.01; 0.35]
Varghese et al. (2013)	83	154	0.54	[0.46; 0.62]
Sinha et al. (2014)	17	42	0.40	[0.26; 0.57]
Abhilash et. al. (2015)	114	189	0.60	[0.53; 0.67]
Stephen et al. (2015)	13	28	0.46	[0.28; 0.66]
Sharma et. al. (2015)	17	23	0.74	[0.52; 0.90]
Sivarajan et al. (2016)	21	90	0.23	[0.15; 0.33]
Ahmad et al. (2016)	7	65	0.11	[0.04; 0.21]
Anithraj et. al. (2016)	48	140	0.34	[0.26; 0.43]
Takhar et al. (2017)	18	66	0.27	[0.17; 0.40]
Mokta et. al. (2017)	72	103	0.70	[0.60; 0.79]
Rawat et al. (2018)	78	168	0.46	[0.39; 0.54]
Bal et al. (2019)	25	201	0.12	[0.08; 0.18]
Gaba et al. (2019)	79	123	0.64	[0.55; 0.73]
Jain et al. (2019)	23	39	0.59	[0.42; 0.74]
Verma et. al. (2021)	31	52	0.60	[0.45; 0.73]
Khan et. al. (2017)	42	104	0.40	[0.31; 0.50]
Dave et al. (2022)	9	173	0.05	[0.02; 0.10]
Varghese et. al. (2013)	12	16	0.75	[0.48; 0.93]
Varghese et. al. (2014)	335	623	0.54	[0.50; 0.58]
Kumar et. al. (2021)	41	126	0.33	[0.24; 0.41]
Nallasamy et. al. (2020)	55	160	0.34	[0.27; 0.42]
Jayaprakash et. al. (2019)	41	193	0.21	[0.16; 0.28]
Behera et al. (2019)	39	114	0.34	[0.26; 0.44]
Ralph et. al. (2019)	12	18	0.67	[0.41; 0.87]
Stephen et. al. (2018)	50	156	0.32	[0.25; 0.40]
Dhar et. al. (2018)	31	65	0.48	[0.35; 0.60]
Bhat et. al. (2016)	54	96	0.56	[0.46; 0.66]
Agarwal et. al. (2014)	41	96	0.43	[0.33; 0.53]
Aggarwal et al. (2015)	13	25	0.52	[0.31; 0.72]
Mutkule et al. (2015)	30	58	0.52	[0.38; 0.65]
**Random effects model**		**3695**	**0.43**	**[0.35; 0.51]**
Heterogeneity: $I^2 = 91\%$, $\tau^2 = 0.8119$, $p < 0.01$				
**clincal_manifestation = Jaundice/Icterus**				
Mathai et al. (2003)	7	28	0.25	[0.11; 0.45]
Mathai et al. (2003b)	1	5	0.20	[0.01; 0.72]
Mahajan et. al. (2006a)	11	21	0.52	[0.30; 0.74]
Varghese et al. (2006)	6	51	0.12	[0.04; 0.24]
Ahmad et. al. (2010)	6	9	0.67	[0.30; 0.93]
Razak et. al. (2010)	10	29	0.34	[0.18; 0.54]
Chrispal et. al. (2010)	25	189	0.13	[0.09; 0.19]
Mahajan et. al. (2010)	4	4	1.00	[0.40; 1.00]
Narvencar et al. (2012)	8	15	0.53	[0.27; 0.79]
Narvencar et al. (2012)	8	15	0.53	[0.27; 0.79]
Vikrant et al. (2013)	9	174	0.05	[0.02; 0.10]
Vikrant et al. (2013)	19	174	0.11	[0.07; 0.17]
Poomalar & Rekha (2014)	1	8	0.12	[0.00; 0.53]
Bhat et. al. (2014a)	6	66	0.09	[0.03; 0.19]
Kumar et al. (2014)	21	49	0.43	[0.29; 0.58]
Jamil et. al. (2015)	7	13	0.54	[0.25; 0.81]
Sharma et. al. (2015)	6	23	0.26	[0.10; 0.48]
Bhargava et. al. (2016)	14	284	0.05	[0.03; 0.08]
Sharma et. al. (2016)	72	228	0.32	[0.26; 0.38]
Sood et al. (2016)	4	6	0.67	[0.22; 0.96]
Meena et. al. (2015)	4	6	0.67	[0.22; 0.96]
Takhar et al. (2017)	33	66	0.50	[0.37; 0.63]
Thangaraj et al. (2018)	15	155	0.10	[0.06; 0.15]
Mokta et. al. (2017)	79	103	0.77	[0.67; 0.84]
Rana et. al. (2017)	3	37	0.08	[0.02; 0.22]
Rawat et al. (2018)	7	168	0.04	[0.02; 0.08]
Gaba et al. (2019)	40	123	0.33	[0.24; 0.42]
Jain et al. (2019)	16	39	0.41	[0.26; 0.58]
Vikram et al. (2020)	4	35	0.11	[0.03; 0.27]
Bhattacharya et. al. (2020)	47	78	0.60	[0.49; 0.71]
Kispotta et al. (2020)	1	262	0.00	[0.00; 0.02]
Bal et al. (2021)	23	45	0.51	[0.36; 0.66]
[illegible]	[illegible]	[illegible]	[illegible]	[illegible]

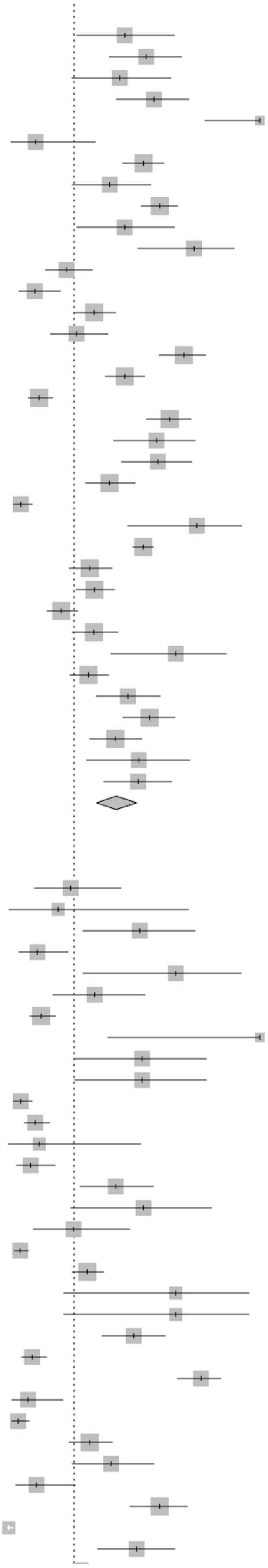

Verma et. al. (2021)	15	52	0.29 [0.17; 0.43]
Bansod et. al. (2021)	5	140	0.04 [0.01; 0.08]
Agrawal et. al. (2022)	4	209	0.02 [0.01; 0.05]
Husain et. al. (2022)	5	361	0.01 [0.00; 0.03]
John et. al. (2023)	4	198	0.02 [0.01; 0.05]
Yadav et al. (2023)	8	27	0.30 [0.14; 0.50]
Mahajan et. al. (2006b)	11	21	0.52 [0.30; 0.74]
Mahajan et. al. (2009)	2	5	0.40 [0.05; 0.85]
Palanivel et. al. (2012)	15	67	0.22 [0.13; 0.34]
Mittal et al. (2012)	3	90	0.03 [0.01; 0.09]
Varghese et. al. (2013)	5	16	0.31 [0.11; 0.59]
Ramasamy et. al. (2023)	2	49	0.04 [0.00; 0.14]
Ramachandran et. al. (2023)	1	31	0.03 [0.00; 0.17]
Shiva et. al. (2022)	3	11	0.27 [0.06; 0.61]
Alam et. al. (2022)	15	50	0.30 [0.18; 0.45]
Sultan et. al. (2022)	10	112	0.09 [0.04; 0.16]
Behera et al. (2021)	7	76	0.09 [0.04; 0.18]
Kumar et. al. (2021)	46	126	0.37 [0.28; 0.46]
Nallasamy et. al. (2020)	14	160	0.09 [0.05; 0.14]
Sivaprakasam et. al. (2020)	1	39	0.03 [0.00; 0.13]
Jayaprakash et. al. (2019)	12	193	0.06 [0.03; 0.11]
Basu et al. (2019)	2	61	0.03 [0.00; 0.11]
Behera et al. (2019)	11	114	0.10 [0.05; 0.17]
Dhar et. al. (2018)	10	65	0.15 [0.08; 0.26]
Karanth et. al. (2014)	27	255	0.11 [0.07; 0.15]
Aggarwal et al. (2015)	3	25	0.12 [0.03; 0.31]
Zainab et. al. (2018)	2	10	0.20 [0.03; 0.56]
Kumar et al. (2018b)	14	50	0.28 [0.16; 0.42]
Balasubramanian et. al. (2016)	14	50	0.28 [0.16; 0.42]
Masand et. al. (2016)	1	30	0.03 [0.00; 0.17]
Bhat et. al. (2014b)	7	62	0.11 [0.05; 0.22]
Jamil et. al. (2014)	9	59	0.15 [0.07; 0.27]
**Random effects model**		**5322**	**0.18 [0.13; 0.24]**
Heterogeneity: $I^2 = 92\%$, $\tau^2 = 1.7726$, $p < 0.01$			
**clincal_manifestation = Other Gastric Abnormalities**			
Mathai et al. (2003)	13	28	0.46 [0.28; 0.66]
Mahajan et. al. (2006a)	15	21	0.71 [0.48; 0.89]
Varghese et al. (2006)	28	51	0.55 [0.40; 0.69]
Damodar et. al. (2023)	29	87	0.33 [0.24; 0.44]
Mittal et al. (2012)	1	90	0.01 [0.00; 0.06]
Ramasamy et. al. (2023)	25	49	0.51 [0.36; 0.66]
Bhat et. al. (2016)	54	96	0.56 [0.46; 0.66]
Sugumar et. al. (2020)	10	24	0.42 [0.22; 0.63]
Basu et al. (2019)	33	61	0.54 [0.41; 0.67]
Karthika et. al. (2021)	58	124	0.47 [0.38; 0.56]
Rehani et. al. (2024)	2	7	0.29 [0.04; 0.71]
**Random effects model**		**638**	**0.43 [0.25; 0.62]**
Heterogeneity: $I^2 = 73\%$, $\tau^2 = 1.2061$, $p < 0.01$			
**clincal_manifestation = Vomitting**			
Mahajan et. al. (2006a)	9	21	0.43 [0.22; 0.66]
Somashekar et al. (2006)	12	27	0.44 [0.25; 0.65]
Ahmad et. al. (2010)	9	9	1.00 [0.66; 1.00]
Razak et. al. (2010)	13	29	0.45 [0.26; 0.64]
Chrispal et. al. (2010)	70	189	0.37 [0.30; 0.44]
Dass et. al. (2011)	5	24	0.21 [0.07; 0.42]
Kumar et al. (2012)	17	35	0.49 [0.31; 0.66]
Khan et. al. (2012)	2	18	0.11 [0.01; 0.35]
Vikrant et al. (2013)	33	174	0.19 [0.13; 0.26]
Varghese et al. (2013)	83	154	0.54 [0.46; 0.62]
Gurung et al. (2013)	10	63	0.16 [0.08; 0.27]
Poomalar & Rekha (2014)	3	8	0.38 [0.09; 0.76]
Sethi et. al. (2014)	27	45	0.60 [0.44; 0.74]
Bhat et. al. (2014a)	37	66	0.56 [0.43; 0.68]
Kumar et al. (2014)	15	49	0.31 [0.18; 0.45]
Krishna et. al. (2015)	23	52	0.44 [0.30; 0.59]
Kumar et al. (2015)	2	14	0.14 [0.02; 0.43]
Narayanasamy et al. (2016)	25	117	0.21 [0.14; 0.30]
Rajan et al. (2016)	9	33	0.27 [0.13; 0.46]
Arun Babu et al. (2017)	61	107	0.57 [0.47; 0.67]
Sivarajan et al. (2016)	21	90	0.23 [0.15; 0.33]
Bhargava et. al. (2016)	113	284	0.40 [0.34; 0.46]
Mahajan et. al. (2016a)	38	253	0.15 [0.11; 0.20]

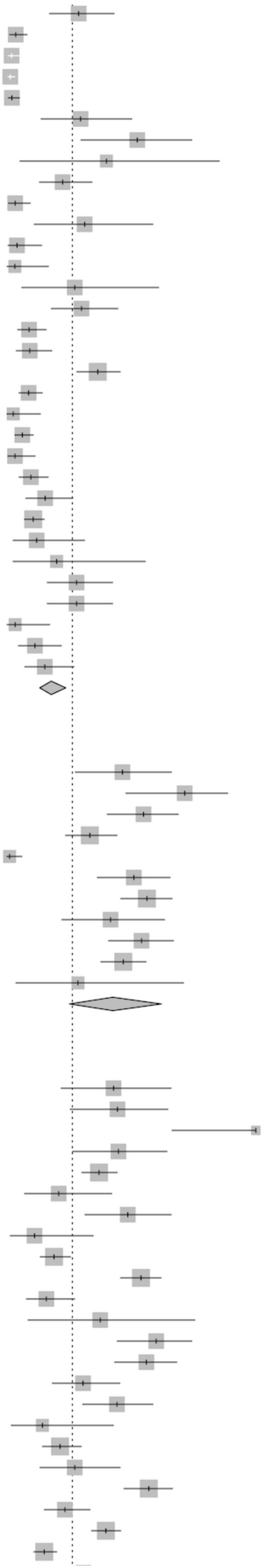

Ahmad et al. (2016)	20	65	0.31 [0.20; 0.43]
Sharma et. al. (2016)	52	228	0.23 [0.18; 0.29]
Anithraj et. al. (2016)	66	140	0.47 [0.39; 0.56]
Sarma et. al. (2017)	4	5	0.80 [0.28; 0.99]
Thangaraj et al. (2017)	7	40	0.17 [0.07; 0.33]
Thangaraj et al. (2018)	34	155	0.22 [0.16; 0.29]
Mokta et. al. (2017)	72	103	0.70 [0.60; 0.79]
Rana et. al. (2017)	5	37	0.14 [0.05; 0.29]
Ganesh et. al. (2018)	132	358	0.37 [0.32; 0.42]
Kumar et al. (2018a)	7	14	0.50 [0.23; 0.77]
Gaba et al. (2019)	46	123	0.37 [0.29; 0.47]
Bhise et al. (2020)	5	158	0.03 [0.01; 0.07]
Lakshmi et al. (2020)	12	89	0.13 [0.07; 0.22]
Alam et. al. (2020)	19	66	0.29 [0.18; 0.41]
Kispotta et al. (2020)	79	262	0.30 [0.25; 0.36]
Kaur et. al. (2020)	3	8	0.38 [0.09; 0.76]
Bal et al. (2021)	34	45	0.76 [0.60; 0.87]
Das et. al. (2021)	24	92	0.26 [0.17; 0.36]
Khemka et al. (2021)	17	62	0.27 [0.17; 0.40]
Bansod et. al. (2021)	2	140	0.01 [0.00; 0.05]
Agrawal et. al. (2022)	48	209	0.23 [0.17; 0.29]
Dave et al. (2022)	13	173	0.08 [0.04; 0.13]
Husain et. al. (2022)	15	361	0.04 [0.02; 0.07]
John et. al. (2023)	44	198	0.22 [0.17; 0.29]
Damodar et. al. (2023)	46	87	0.53 [0.42; 0.64]
Jana et. al. (2023)	46	206	0.22 [0.17; 0.29]
Narayanasamy et. al (2023)	364	560	0.65 [0.61; 0.69]
Mahajan et. al. (2008)	5	5	1.00 [0.48; 1.00]
Mahajan et. al. (2009)	4	5	0.80 [0.28; 0.99]
Palanivel et. al. (2012)	40	67	0.60 [0.47; 0.72]
Mittal et al. (2012)	11	90	0.12 [0.06; 0.21]
Varghese et. al. (2013)	12	16	0.75 [0.48; 0.93]
Varghese et. al. (2014)	335	623	0.54 [0.50; 0.58]
Ramasamy et. al. (2023)	14	49	0.29 [0.17; 0.43]
Rama et. al. (2019)	1	5	0.20 [0.01; 0.72]
Chunduru et. al. (2023)	58	214	0.27 [0.21; 0.34]
Vikas et. al. (2023)	4	8	0.50 [0.16; 0.84]
Sultan et. al. (2022)	58	112	0.52 [0.42; 0.61]
Behera et al. (2021)	24	76	0.32 [0.21; 0.43]
Loganathan et. al. (2021)	153	431	0.35 [0.31; 0.40]
Thakur et. al. (2019)	52	210	0.25 [0.19; 0.31]
Sivaprakasam et. al. (2020)	13	39	0.33 [0.19; 0.50]
Basu et al. (2019)	39	61	0.64 [0.51; 0.76]
Ralph et. al. (2019)	12	18	0.67 [0.41; 0.87]
Stephen et. al. (2018)	57	156	0.37 [0.29; 0.45]
Dhar et. al. (2018)	31	65	0.48 [0.35; 0.60]
Karanth et. al. (2014)	65	255	0.25 [0.20; 0.31]
Agarwal et. al. (2014)	42	96	0.44 [0.34; 0.54]
Aggarwal et al. (2015)	13	25	0.52 [0.31; 0.72]
Valappil et. al. (2017)	20	28	0.71 [0.51; 0.87]
Mittal et. al. (2017)	17	46	0.37 [0.23; 0.52]
Sivathanu et. al. (2017)	23	31	0.74 [0.55; 0.88]
Balasubramanian et. al. (2016)	10	50	0.20 [0.10; 0.34]
Masand et. al. (2016)	3	30	0.10 [0.02; 0.27]
Misra et. al. (2014)	27	37	0.73 [0.56; 0.86]
Mutkule et al. (2015)	30	58	0.52 [0.38; 0.65]
Bhat et. al. (2014b)	37	62	0.60 [0.46; 0.72]
Jamil et. al. (2014)	10	59	0.17 [0.08; 0.29]
Bhat et. al. (2016)	54	96	0.56 [0.46; 0.66]
Rose et. al. (2017)	151	427	0.35 [0.31; 0.40]
**Random effects model**		**9395**	**0.35 [0.30; 0.41]**
Heterogeneity: I^2 = 92%, τ^2 = 0.9830, p < 0.01			
**clincal_manifestation = Abdominal Pain**			
Mahajan et. al. (2006a)	6	21	0.29 [0.11; 0.52]
Vivekanandan et. al. (2010)	10	50	0.20 [0.10; 0.34]
Razak et. al. (2010)	12	29	0.41 [0.24; 0.61]
Chrispal et. al. (2010)	41	189	0.22 [0.16; 0.28]
Mahajan et. al. (2010)	2	4	0.50 [0.07; 0.93]
Dass et. al. (2011)	6	24	0.25 [0.10; 0.47]
Narvencar et al. (2012)	7	15	0.47 [0.21; 0.73]
Kumar et al. (2012)	12	35	0.34 [0.19; 0.52]
Khan et. al. (2012)	2	18	0.11 [0.01; 0.35]
Gupta et. al. (2012)	1	5	0.20 [0.01; 0.72]

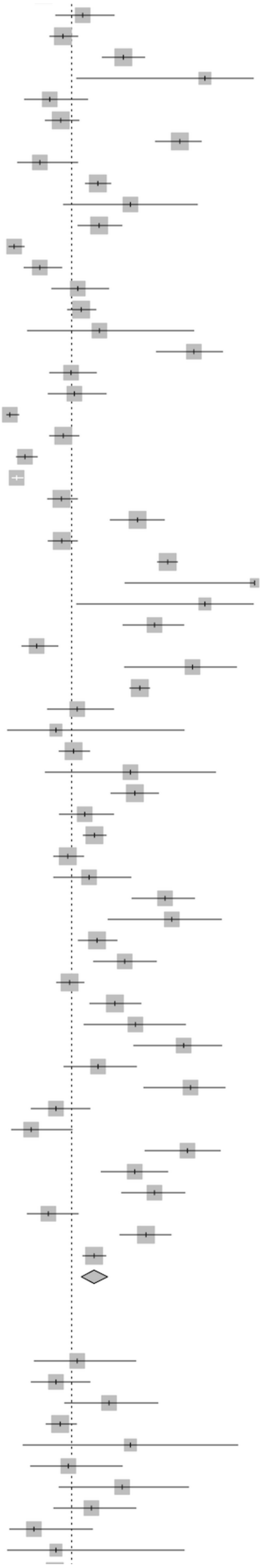

Vikrant et al. (2013)	34	174	0.20 [0.14; 0.26]
Vikrant et al. (2013)	14	174	0.08 [0.04; 0.13]
Viswanathan et. al. (2013)	37	65	0.57 [0.44; 0.69]
Sinha et al. (2014)	6	42	0.14 [0.05; 0.29]
Poomalar & Rekha (2014)	2	8	0.25 [0.03; 0.65]
Bhat et. al. (2014a)	22	66	0.33 [0.22; 0.46]
Kumar et al. (2014)	15	49	0.31 [0.18; 0.45]
Stephen et al. (2015)	12	28	0.43 [0.24; 0.63]
Kumar et al. (2015)	4	14	0.29 [0.08; 0.58]
Narayanasamy et al. (2016)	50	117	0.43 [0.34; 0.52]
Sivarajan et al. (2016)	24	90	0.27 [0.18; 0.37]
Bhargava et. al. (2016)	66	284	0.23 [0.18; 0.29]
Farhana et al. (2016)	18	37	0.49 [0.32; 0.66]
Mahajan et. al. (2016a)	53	253	0.21 [0.16; 0.26]
Ahmad et al. (2016)	21	65	0.32 [0.21; 0.45]
Sharma et. al. (2016)	63	228	0.28 [0.22; 0.34]
Anithraj et. al. (2016)	44	140	0.31 [0.24; 0.40]
Thangaraj et al. (2017)	12	40	0.30 [0.17; 0.47]
Thangaraj et al. (2018)	27	155	0.17 [0.12; 0.24]
Mokta et. al. (2017)	40	103	0.39 [0.29; 0.49]
Rana et. al. (2017)	3	37	0.08 [0.02; 0.22]
Ganesh et. al. (2018)	82	358	0.23 [0.19; 0.28]
Rawat et al. (2018)	8	168	0.05 [0.02; 0.09]
Kumar et al. (2018a)	5	14	0.36 [0.13; 0.65]
Bal et al. (2019)	13	201	0.06 [0.03; 0.11]
Vikram et al. (2020)	18	35	0.51 [0.34; 0.69]
Kispotta et al. (2020)	33	262	0.13 [0.09; 0.17]
Williams et al. (2021)	25	72	0.35 [0.24; 0.47]
Khemka et al. (2021)	21	62	0.34 [0.22; 0.47]
Bansod et. al. (2021)	9	140	0.06 [0.03; 0.12]
Agrawal et. al. (2022)	49	209	0.23 [0.18; 0.30]
Husain et. al. (2022)	10	361	0.03 [0.01; 0.05]
John et. al. (2023)	22	198	0.11 [0.07; 0.16]
Jana et. al. (2023)	35	206	0.17 [0.12; 0.23]
Mahajan et. al. (2006b)	6	21	0.29 [0.11; 0.52]
Mahajan et. al. (2008)	1	5	0.20 [0.01; 0.72]
Mahajan et. al. (2009)	2	5	0.40 [0.05; 0.85]
Mittal et al. (2012)	6	90	0.07 [0.02; 0.14]
Kalita et. al. (2015)	8	33	0.24 [0.11; 0.42]
Ramasamy et. al. (2023)	10	49	0.20 [0.10; 0.34]
Bharathi et. al. (2023)	1	28	0.04 [0.00; 0.18]
Vindhiya et. al. (2023)	1	5	0.20 [0.01; 0.72]
Alam et. al. (2022)	15	50	0.30 [0.18; 0.45]
Chunduru et. al. (2023)	56	214	0.26 [0.20; 0.33]
Sultan et. al. (2022)	20	112	0.18 [0.11; 0.26]
Behera et al. (2021)	23	76	0.30 [0.20; 0.42]
Loganathan et. al. (2021)	90	431	0.21 [0.17; 0.25]
Kumar et. al. (2021)	62	126	0.49 [0.40; 0.58]
Thakur et. al. (2019)	33	210	0.16 [0.11; 0.21]
Nallasamy et. al. (2020)	37	160	0.23 [0.17; 0.30]
Sivaprakasam et. al. (2020)	11	39	0.28 [0.15; 0.45]
Basu et al. (2019)	33	61	0.54 [0.41; 0.67]
Behera et al. (2019)	38	114	0.33 [0.25; 0.43]
Stephen et. al. (2018)	96	156	0.62 [0.53; 0.69]
Dhar et. al. (2018)	27	65	0.42 [0.29; 0.54]
Kakarlapudi et. al. (2018)	9	48	0.19 [0.09; 0.33]
Bhat et. al. (2016)	28	96	0.29 [0.20; 0.39]
Karanth et. al. (2014)	60	255	0.24 [0.18; 0.29]
Aggarwal et al. (2015)	12	25	0.48 [0.28; 0.69]
Zainab et. al. (2018)	5	10	0.50 [0.19; 0.81]
Kumar et al. (2018b)	13	50	0.26 [0.15; 0.40]
Sivathanu et. al. (2017)	13	31	0.42 [0.25; 0.61]
Balasubramanian et. al. (2016)	13	50	0.26 [0.15; 0.40]
Masand et. al. (2016)	5	30	0.17 [0.06; 0.35]
Misra et. al. (2014)	18	37	0.49 [0.32; 0.66]
Mutkule et al. (2015)	27	58	0.47 [0.33; 0.60]
Bhat et. al. (2014b)	22	62	0.35 [0.24; 0.49]
Bhat et. al. (2016)	28	96	0.29 [0.20; 0.39]
Rose et. al. (2017)	90	427	0.21 [0.17; 0.25]
**Random effects model**		**8170**	**0.26 [0.23; 0.29]**

Heterogeneity: $I^2 = 87\%$, $\tau^2 = 0.5061$, $p < 0.01$

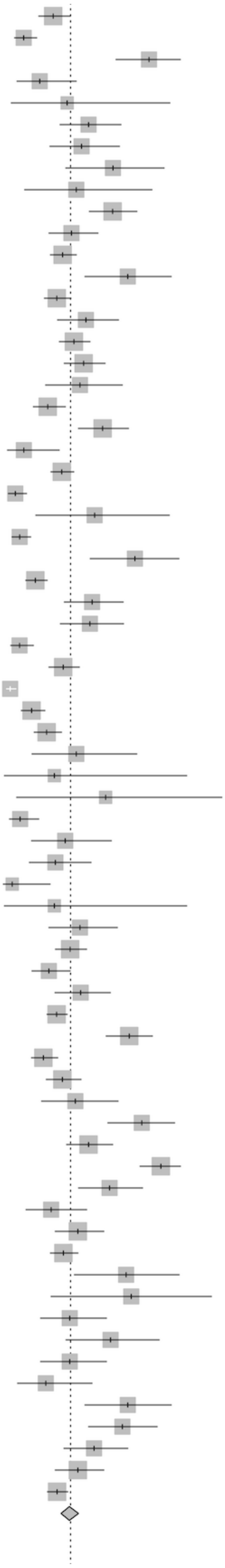

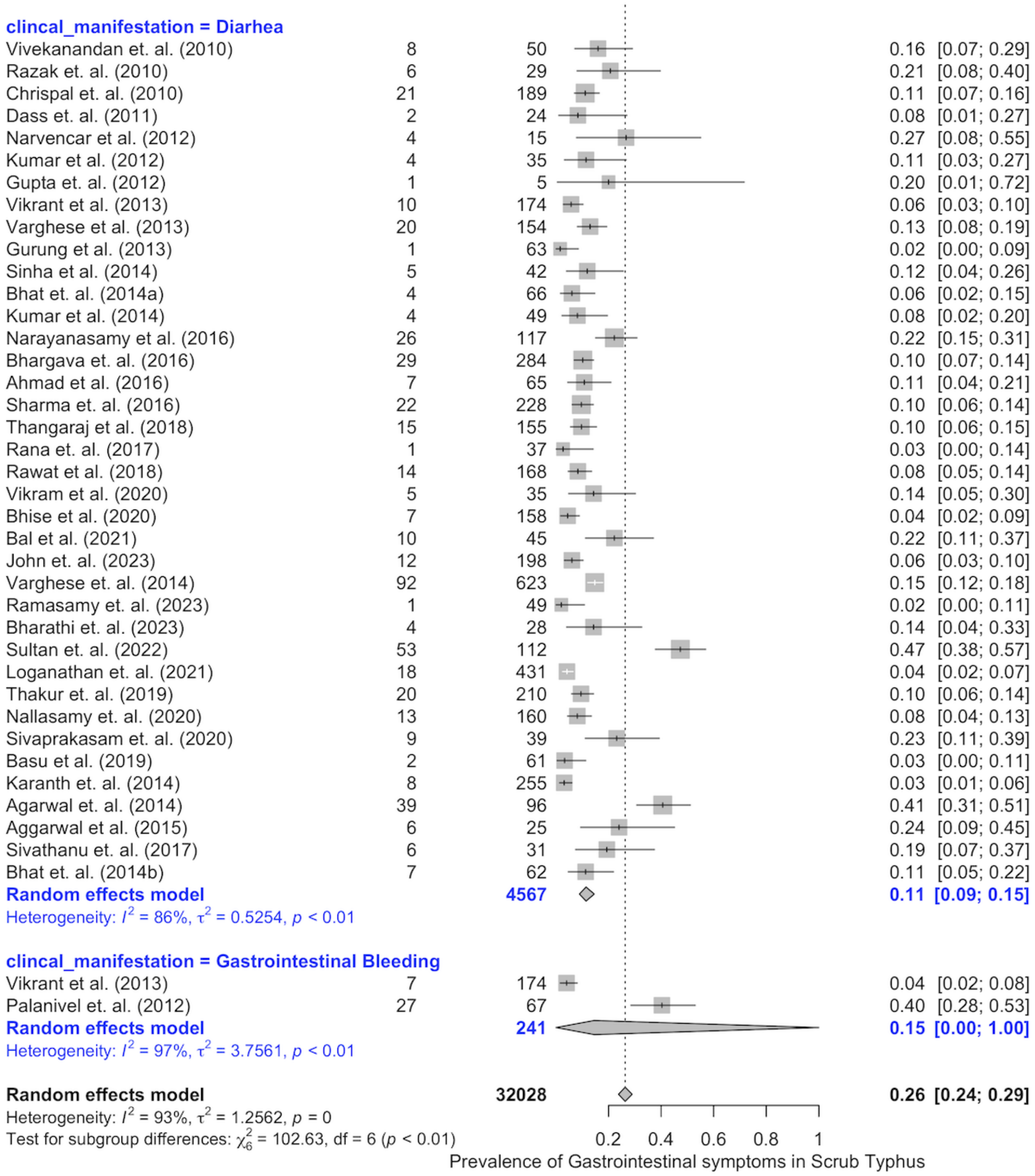

Forest plot 4. Pooled prevalence of different types of gastrointestinal symptoms of scrub typhus

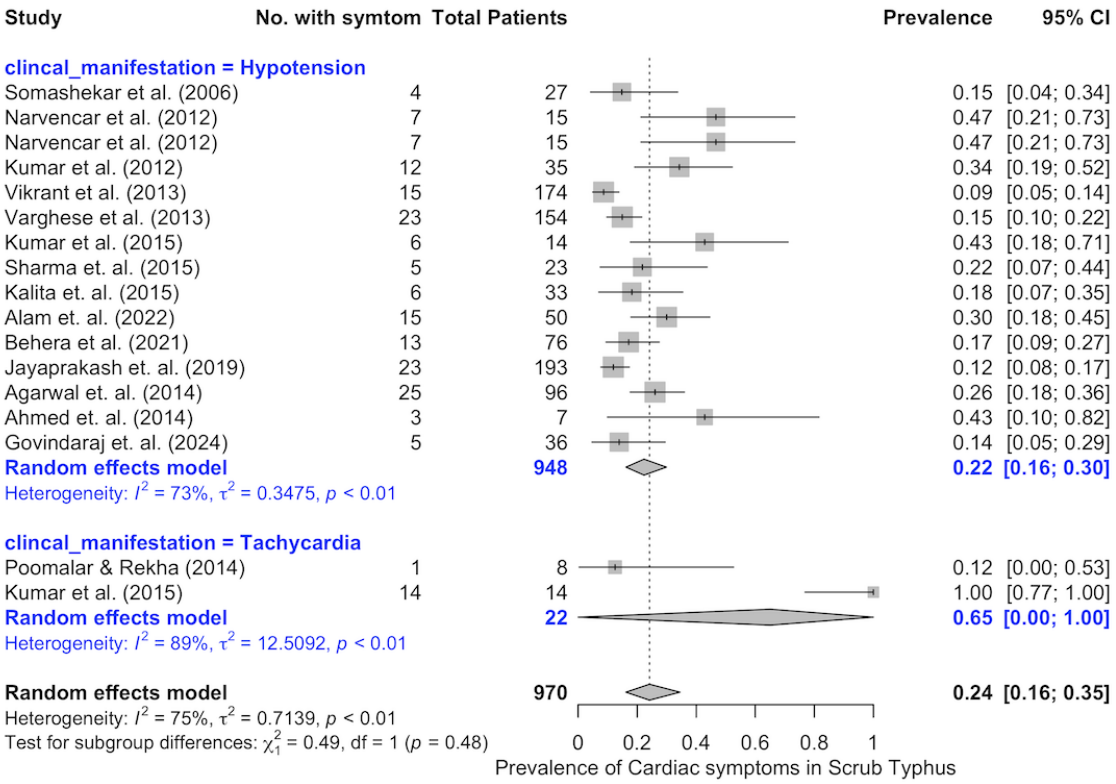

Forest plot 5. Pooled prevalence of different types of cardiac symptoms of scrub typhus

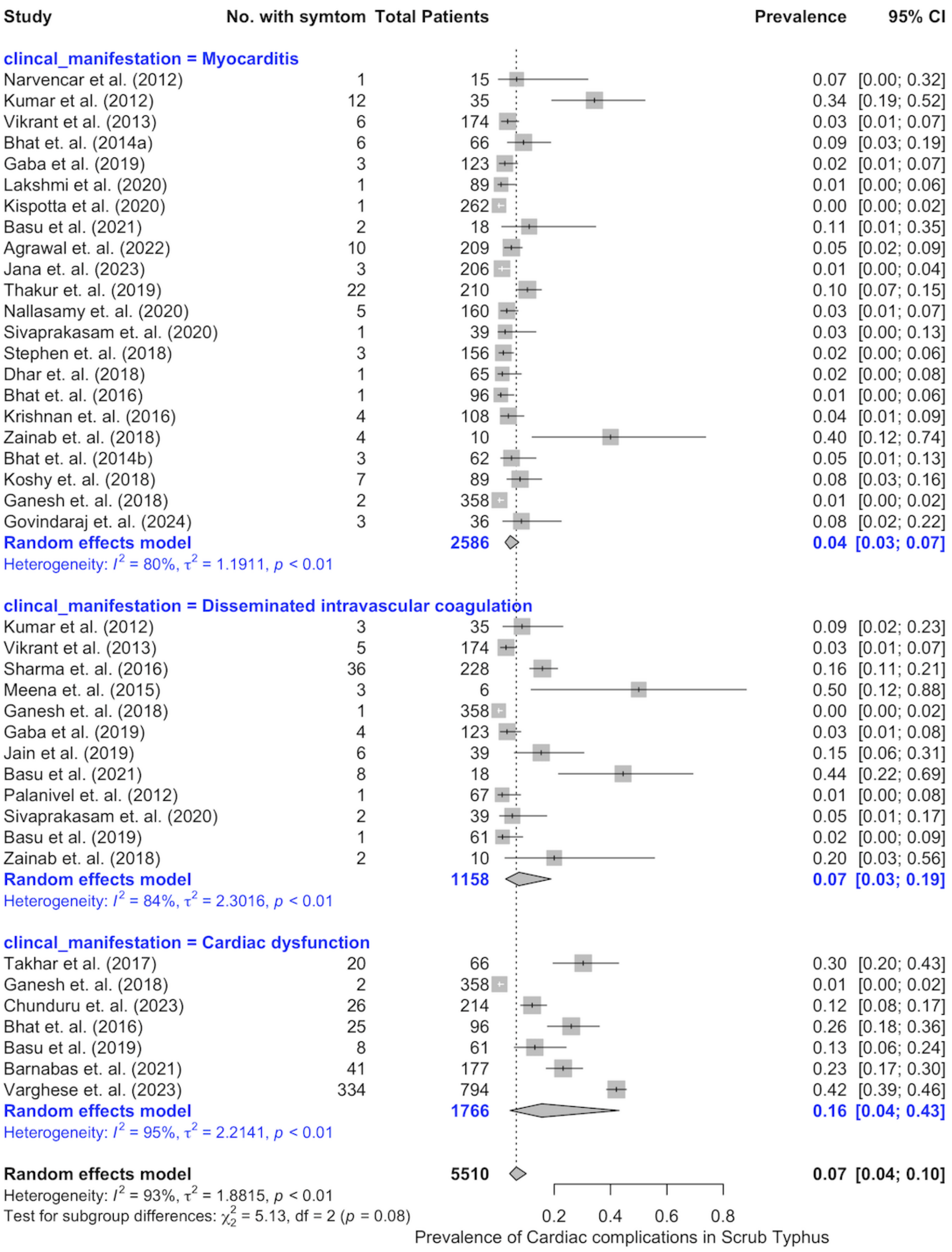

Forest plot 6. Pooled prevalence of different types of cardiac complications of scrub typhus

Study	No. with symtom	Total Patients	Prevalence	95% CI
**clincal_manifestation = Other Liver problems**				
Mathai et al. (2003)	7	28	0.25	[0.11; 0.45]
Mathai et al. (2003b)	1	5	0.20	[0.01; 0.72]
Sharma et al. (2005)	13	45	0.29	[0.16; 0.44]
Varghese et al. (2006)	6	51	0.12	[0.04; 0.24]
Mahajan et. al. (2016a)	124	253	0.49	[0.43; 0.55]
Singla et. al. (2020)	10	77	0.13	[0.06; 0.23]
Bhat et. al. (2016)	75	96	0.78	[0.69; 0.86]
Sugumar et. al. (2020)	4	24	0.17	[0.05; 0.37]
Ganesh et. al. (2018)	339	358	0.95	[0.92; 0.97]
Narayanasamy et. al (2018)	6	230	0.03	[0.01; 0.06]
Rauf et. al. (2018)	23	23	1.00	[0.85; 1.00]
Basu et al. (2019)	25	61	0.41	[0.29; 0.54]
Barnabas et. al. (2021)	111	177	0.63	[0.55; 0.70]
Basu et al. (2021)	17	18	0.94	[0.73; 1.00]
Veerappan et. al. (2021)	5	155	0.03	[0.01; 0.07]
Narayanasamy et. al (2021)	137	256	0.54	[0.47; 0.60]
Roychowdhary et. al. (2022)	35	105	0.33	[0.24; 0.43]
Karthika et. al. (2021)	38	124	0.31	[0.23; 0.40]
Varghese et. al. (2023)	425	794	0.54	[0.50; 0.57]
**Random effects model**		**2880**	**0.40**	**[0.21; 0.62]**
Heterogeneity: $I^2 = 96\%$, $\tau^2 = 3.3352$, $p < 0.01$				
**clincal_manifestation = Hepatomegaly**				
Mahajan et. al. (2006a)	9	21	0.43	[0.22; 0.66]
Mahajan et. al. (2006a)	1	21	0.05	[0.00; 0.24]
Somashekar et al. (2006)	16	27	0.59	[0.39; 0.78]
Vivekanandan et. al. (2010)	14	50	0.28	[0.16; 0.42]
Ahmad et. al. (2010)	7	9	0.78	[0.40; 0.97]
Razak et. al. (2010)	16	29	0.55	[0.36; 0.74]
Chrispal et. al. (2010)	70	189	0.37	[0.30; 0.44]
Vaz & Gupta (2006)	1	12	0.08	[0.00; 0.38]
Sankhyan et. al. (2014)	14	15	0.93	[0.68; 1.00]
Dass et. al. (2011)	8	24	0.33	[0.16; 0.55]
Rathi et al. (2011)	22	23	0.96	[0.78; 1.00]
Narvencar et al. (2012)	9	15	0.60	[0.32; 0.84]
Kumar et al. (2012)	32	35	0.91	[0.77; 0.98]
Gupta et. al. (2012)	3	5	0.60	[0.15; 0.95]
Vikrant et al. (2013)	25	174	0.14	[0.10; 0.20]
Viswanathan et. al. (2013)	26	65	0.40	[0.28; 0.53]
Gurung et al. (2013)	17	63	0.27	[0.17; 0.40]
Sinha et al. (2014)	25	42	0.60	[0.43; 0.74]
Sethi et. al. (2014)	22	45	0.49	[0.34; 0.64]
Bhat et. al. (2014a)	54	66	0.82	[0.70; 0.90]
Kumar et al. (2014)	34	49	0.69	[0.55; 0.82]
Stephen et al. (2015)	11	28	0.39	[0.22; 0.59]
Jamil et. al. (2015)	5	13	0.38	[0.14; 0.68]
Krishna et. al. (2015)	49	52	0.94	[0.84; 0.99]
Narayanasamy et al. (2016)	82	117	0.70	[0.61; 0.78]
Rajan et al. (2016)	4	33	0.12	[0.03; 0.28]
Arun Babu et al. (2017)	80	107	0.75	[0.65; 0.83]
Sivarajan et al. (2016)	24	90	0.27	[0.18; 0.37]
Bhargava et. al. (2016)	94	284	0.33	[0.28; 0.39]
Mahajan et. al. (2016a)	33	253	0.13	[0.09; 0.18]
Basheer et al. (2016)	1	6	0.17	[0.00; 0.64]
Ahmad et al. (2016)	20	65	0.31	[0.20; 0.43]
Sood et al. (2016)	3	6	0.50	[0.12; 0.88]
Anithraj et. al. (2016)	24	140	0.17	[0.11; 0.24]
Meena et. al. (2015)	1	6	0.17	[0.00; 0.64]
Takhar et al. (2017)	23	66	0.35	[0.24; 0.48]
Thangaraj et al. (2017)	12	40	0.30	[0.17; 0.47]
Thangaraj et al. (2018)	6	155	0.04	[0.01; 0.08]
Rana et. al. (2017)	2	37	0.05	[0.01; 0.18]
Ganesh et. al. (2018)	339	358	0.95	[0.92; 0.97]
Rawat et al. (2018)	12	168	0.07	[0.04; 0.12]
Kumar et al. (2018a)	4	14	0.29	[0.08; 0.58]
Bal et al. (2019)	67	201	0.33	[0.27; 0.40]
Gaba et al. (2019)	53	123	0.43	[0.34; 0.52]
Jain et al. (2019)	18	39	0.46	[0.30; 0.63]
Vikram et al. (2020)	10	35	0.29	[0.15; 0.46]
Singla et. al. (2020)	34	77	0.44	[0.33; 0.56]
Alam et. al. (2020)	42	66	0.64	[0.51; 0.75]

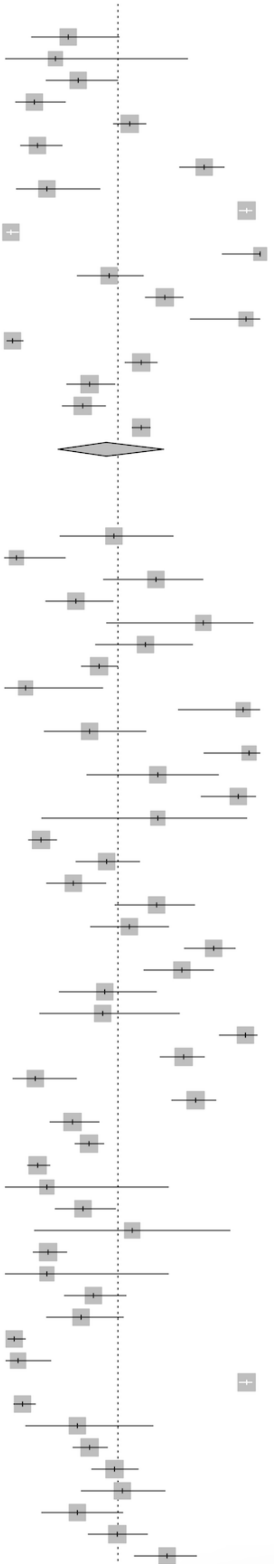

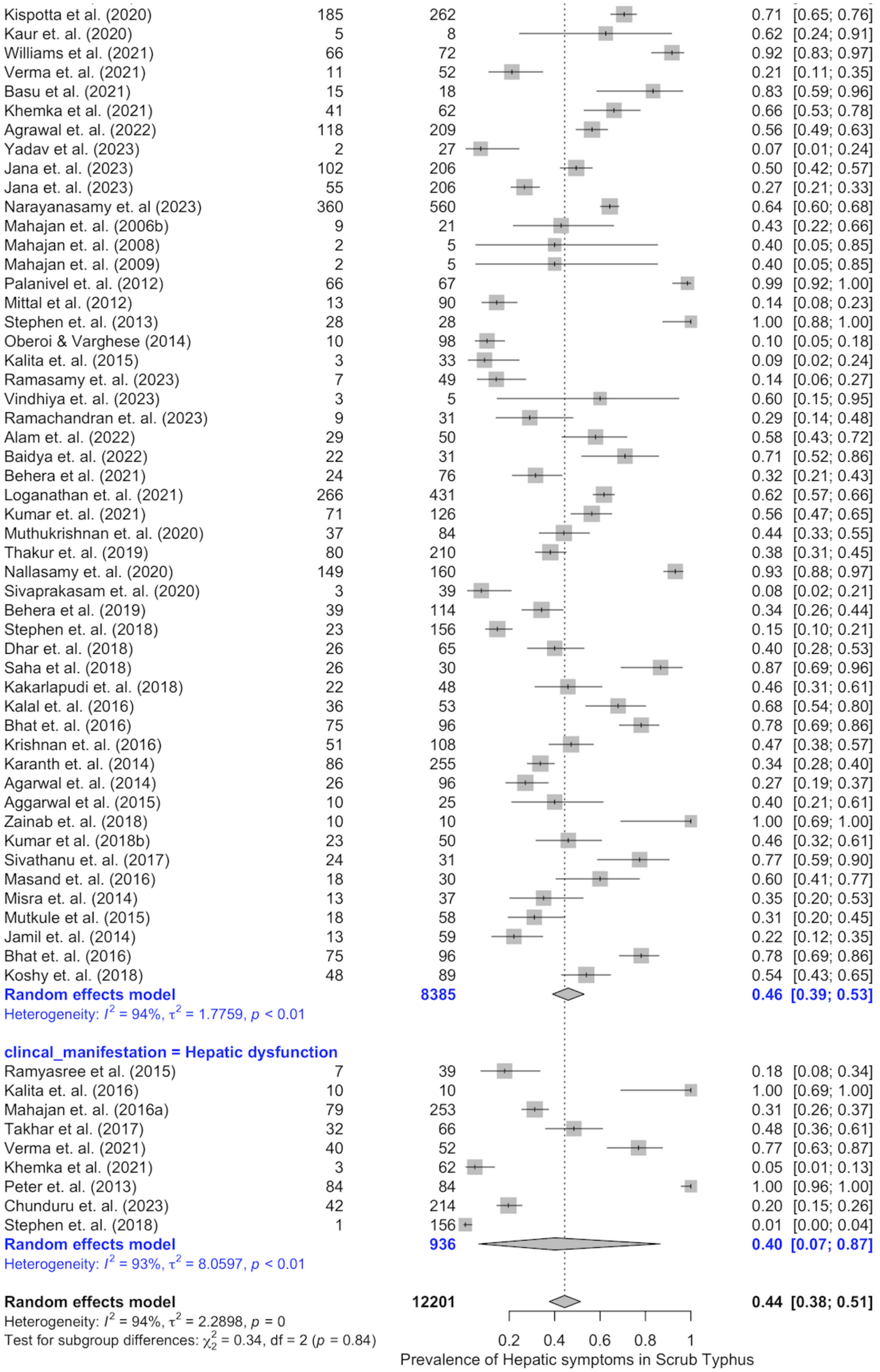

Forest plot 7. Pooled prevalence of different types of hepatic symptoms of scrub typhus

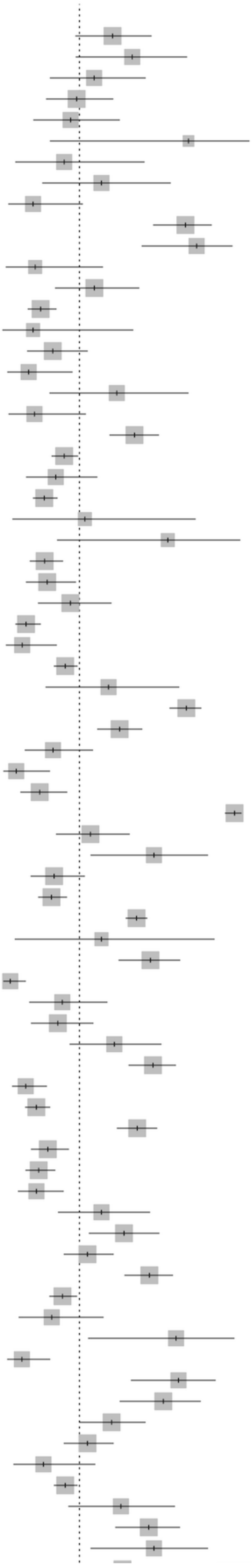

Study	No. with symtom	Total Patients	Prevalence	95% CI
**clincal_manifestation = Lymphadenopathy**				
Sharma et al. (2005)	20	45	0.44	[0.30; 0.60]
Mahajan et. al. (2006a)	11	21	0.52	[0.30; 0.74]
Somashekar et al. (2006)	10	27	0.37	[0.19; 0.58]
Vivekanandan et. al. (2010)	15	50	0.30	[0.18; 0.45]
Razak et. al. (2010)	8	29	0.28	[0.13; 0.47]
Mahajan et. al. (2010)	3	4	0.75	[0.19; 0.99]
Vaz & Gupta (2006)	3	12	0.25	[0.05; 0.57]
Sankhyan et. al. (2014)	6	15	0.40	[0.16; 0.68]
Dass et. al. (2011)	3	24	0.12	[0.03; 0.32]
Thakur et al. (2011)	45	61	0.74	[0.61; 0.84]
Rathi et al. (2011)	18	23	0.78	[0.56; 0.93]
Narvencar et al. (2012)	2	15	0.13	[0.02; 0.40]
Kumar et al. (2012)	13	35	0.37	[0.21; 0.55]
Vikrant et al. (2013)	27	174	0.16	[0.10; 0.22]
Poomalar & Rekha (2014)	1	8	0.12	[0.00; 0.53]
Kumar et al. (2014)	10	49	0.20	[0.10; 0.34]
Stephen et al. (2015)	3	28	0.11	[0.02; 0.28]
Jamil et. al. (2015)	6	13	0.46	[0.19; 0.75]
Sharma et. al. (2015)	3	23	0.13	[0.03; 0.34]
Arun Babu et al. (2017)	57	107	0.53	[0.43; 0.63]
Bhargava et. al. (2016)	71	284	0.25	[0.20; 0.30]
Farhana et al. (2016)	8	37	0.22	[0.10; 0.38]
Mahajan et. al. (2016a)	43	253	0.17	[0.13; 0.22]
Basheer et. al. (2016)	2	6	0.33	[0.04; 0.78]
Sood et al. (2016)	4	6	0.67	[0.22; 0.96]
Anithraj et. al. (2016)	24	140	0.17	[0.11; 0.24]
Takhar et al. (2017)	12	66	0.18	[0.10; 0.30]
Thangaraj et al. (2017)	11	40	0.28	[0.15; 0.44]
Thangaraj et al. (2018)	15	155	0.10	[0.06; 0.15]
Rana et. al. (2017)	3	37	0.08	[0.02; 0.22]
Ganesh et. al. (2018)	91	358	0.25	[0.21; 0.30]
Kumar et al. (2018a)	6	14	0.43	[0.18; 0.71]
Bal et al. (2019)	149	201	0.74	[0.67; 0.80]
Bhattacharya et. al. (2019)	61	129	0.47	[0.38; 0.56]
Jain et al. (2019)	8	39	0.21	[0.09; 0.36]
Vikram et al. (2020)	2	35	0.06	[0.01; 0.19]
Alam et. al. (2020)	10	66	0.15	[0.08; 0.26]
Kispotta et al. (2020)	245	262	0.94	[0.90; 0.96]
Bal et al. (2021)	16	45	0.36	[0.22; 0.51]
Basu et al. (2021)	11	18	0.61	[0.36; 0.83]
Khemka et al. (2021)	13	62	0.21	[0.12; 0.33]
Jana et. al. (2023)	41	206	0.20	[0.15; 0.26]
Narayanasamy et. al (2023)	303	560	0.54	[0.50; 0.58]
Mahajan et. al. (2009)	2	5	0.40	[0.05; 0.85]
Palanivel et. al. (2012)	40	67	0.60	[0.47; 0.72]
Mittal et al. (2012)	3	90	0.03	[0.01; 0.09]
Kalita et. al. (2015)	8	33	0.24	[0.11; 0.42]
Ramasamy et. al. (2023)	11	49	0.22	[0.12; 0.37]
Baidya et. al. (2022)	14	31	0.45	[0.27; 0.64]
Sultan et. al. (2022)	68	112	0.61	[0.51; 0.70]
Muthukrishnan et. al. (2020)	8	84	0.10	[0.04; 0.18]
Thakur et. al. (2019)	29	210	0.14	[0.09; 0.19]
Nallasamy et. al. (2020)	87	160	0.54	[0.46; 0.62]
Behera et al. (2019)	21	114	0.18	[0.12; 0.27]
Stephen et. al. (2018)	23	156	0.15	[0.10; 0.21]
Dhar et. al. (2018)	9	65	0.14	[0.07; 0.25]
Saha et al. (2018)	12	30	0.40	[0.23; 0.59]
Kalal et. al. (2016)	26	53	0.49	[0.35; 0.63]
Bhat et. al. (2016)	33	96	0.34	[0.25; 0.45]
Krishnan et. al. (2016)	64	108	0.59	[0.49; 0.69]
Karanth et. al. (2014)	62	255	0.24	[0.19; 0.30]
Aggarwal et al. (2015)	5	25	0.20	[0.07; 0.41]
Zainab et. al. (2018)	7	10	0.70	[0.35; 0.93]
Kumar et al. (2018b)	4	50	0.08	[0.02; 0.19]
Sivathanu et. al. (2017)	22	31	0.71	[0.52; 0.86]
Misra et. al. (2014)	24	37	0.65	[0.47; 0.80]
Jamil et. al. (2014)	26	59	0.44	[0.31; 0.58]
Bhat et. al. (2016)	33	96	0.34	[0.25; 0.45]
Sugumar et. al. (2020)	4	24	0.17	[0.05; 0.37]
Ganesh et. al. (2018)	91	358	0.25	[0.21; 0.30]
Rauf et. al. (2018)	11	23	0.48	[0.27; 0.69]
Basu et al. (2019)	36	61	0.59	[0.46; 0.71]
Basu et al. (2021)	11	18	0.61	[0.36; 0.83]
[illegible]	[illegible]	[illegible]	[illegible]	[illegible]

Narayanasamy et. al (2021)	124	256	0.48	[0.42; 0.55]
Karthika et. al. (2021)	27	124	0.22	[0.15; 0.30]
**Random effects model**		**6672**	**0.33**	**[0.28; 0.39]**
Heterogeneity: $I^2 = 93\%$, $\tau^2 = 1.0043$, $p < 0.01$				
**clincal_manifestation = Splenomegaly**				
Sharma et al. (2005)	17	45	0.38	[0.24; 0.53]
Mahajan et. al. (2006a)	7	21	0.33	[0.15; 0.57]
Somashekar et al. (2006)	10	27	0.37	[0.19; 0.58]
Vivekanandan et. al. (2010)	10	50	0.20	[0.10; 0.34]
Razak et. al. (2010)	9	29	0.31	[0.15; 0.51]
Chrispal et. al. (2010)	15	189	0.08	[0.05; 0.13]
Vaz & Gupta (2006)	1	12	0.08	[0.00; 0.38]
Sankhyan et. al. (2014)	13	15	0.87	[0.60; 0.98]
Dass et. al. (2011)	11	24	0.46	[0.26; 0.67]
Narvencar et al. (2012)	4	15	0.27	[0.08; 0.55]
Kumar et al. (2012)	21	35	0.60	[0.42; 0.76]
Khan et. al. (2012)	1	18	0.06	[0.00; 0.27]
Gupta et. al. (2012)	3	5	0.60	[0.15; 0.95]
Vikrant et al. (2013)	37	174	0.21	[0.15; 0.28]
Viswanathan et. al. (2013)	15	65	0.23	[0.14; 0.35]
Gurung et al. (2013)	18	63	0.29	[0.18; 0.41]
Sinha et al. (2014)	15	42	0.36	[0.22; 0.52]
Sethi et. al. (2014)	9	45	0.20	[0.10; 0.35]
Bhat et. al. (2014a)	39	66	0.59	[0.46; 0.71]
Kumar et al. (2014)	20	49	0.41	[0.27; 0.56]
Stephen et al. (2015)	10	28	0.36	[0.19; 0.56]
Jamil et. al. (2015)	1	13	0.08	[0.00; 0.36]
Krishna et. al. (2015)	38	52	0.73	[0.59; 0.84]
Sharma et. al. (2015)	6	23	0.26	[0.10; 0.48]
Narayanasamy et al. (2016)	63	117	0.54	[0.44; 0.63]
Rajan et al. (2016)	4	33	0.12	[0.03; 0.28]
Arun Babu et al. (2017)	30	107	0.28	[0.20; 0.38]
Sivarajan et al. (2016)	22	90	0.24	[0.16; 0.35]
Bhargava et. al. (2016)	44	284	0.15	[0.11; 0.20]
Mahajan et. al. (2016a)	43	253	0.17	[0.13; 0.22]
Ahmad et al. (2016)	11	65	0.17	[0.09; 0.28]
Sood et al. (2016)	5	6	0.83	[0.36; 1.00]
Anithraj et. al. (2016)	27	140	0.19	[0.13; 0.27]
Takhar et al. (2017)	23	66	0.35	[0.24; 0.48]
Thangaraj et al. (2017)	1	40	0.03	[0.00; 0.13]
Thangaraj et al. (2018)	4	155	0.03	[0.01; 0.06]
Rana et. al. (2017)	6	37	0.16	[0.06; 0.32]
Ganesh et. al. (2018)	322	358	0.90	[0.86; 0.93]
Rawat et al. (2018)	16	168	0.10	[0.06; 0.15]
Kumar et al. (2018a)	9	14	0.64	[0.35; 0.87]
Bal et al. (2019)	67	201	0.33	[0.27; 0.40]
Gaba et al. (2019)	43	123	0.35	[0.27; 0.44]
Jain et al. (2019)	18	39	0.46	[0.30; 0.63]
Vikram et al. (2020)	8	35	0.23	[0.10; 0.40]
Singla et. al. (2020)	34	77	0.44	[0.33; 0.56]
Alam et. al. (2020)	14	66	0.21	[0.12; 0.33]
Kispotta et al. (2020)	87	262	0.33	[0.28; 0.39]
Kaur et. al. (2020)	1	8	0.12	[0.00; 0.53]
Bal et al. (2021)	18	45	0.40	[0.26; 0.56]
Williams et al. (2021)	43	72	0.60	[0.47; 0.71]
Verma et. al. (2021)	15	52	0.29	[0.17; 0.43]
Basu et al. (2021)	17	18	0.94	[0.73; 1.00]
Khemka et al. (2021)	36	62	0.58	[0.45; 0.70]
Agrawal et. al. (2022)	83	209	0.40	[0.33; 0.47]
Yadav et al. (2023)	1	27	0.04	[0.00; 0.19]
Jana et. al. (2023)	102	206	0.50	[0.42; 0.57]
Jana et. al. (2023)	31	206	0.15	[0.10; 0.21]
Narayanasamy et. al (2023)	323	560	0.58	[0.53; 0.62]
Mahajan et. al. (2006b)	9	21	0.43	[0.22; 0.66]
Mahajan et. al. (2008)	2	5	0.40	[0.05; 0.85]
Mahajan et. al. (2009)	2	5	0.40	[0.05; 0.85]
Palanivel et. al. (2012)	59	67	0.88	[0.78; 0.95]
Mittal et al. (2012)	8	90	0.09	[0.04; 0.17]
Stephen et. al. (2013)	28	28	1.00	[0.88; 1.00]
Oberoi & Varghese (2014)	10	98	0.10	[0.05; 0.18]
Kalita et. al. (2015)	1	33	0.03	[0.00; 0.16]
Ramasamy et. al. (2023)	35	49	0.71	[0.57; 0.83]
Vindhiya et. al. (2023)	3	5	0.60	[0.15; 0.95]

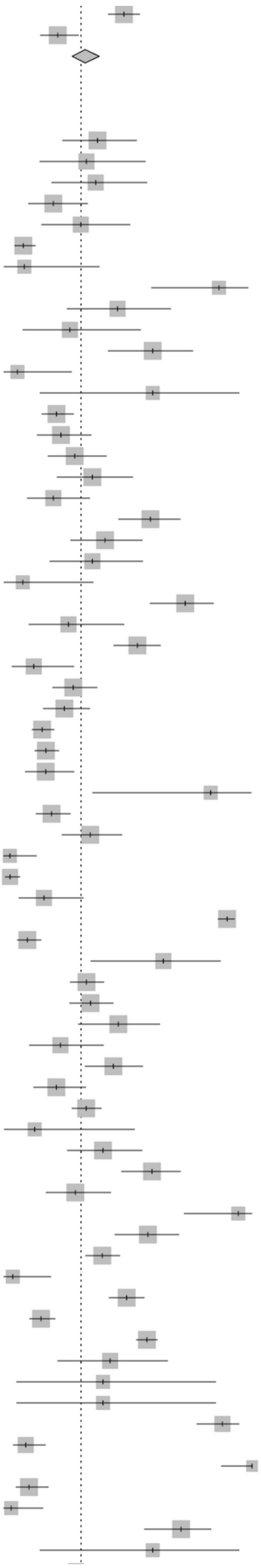

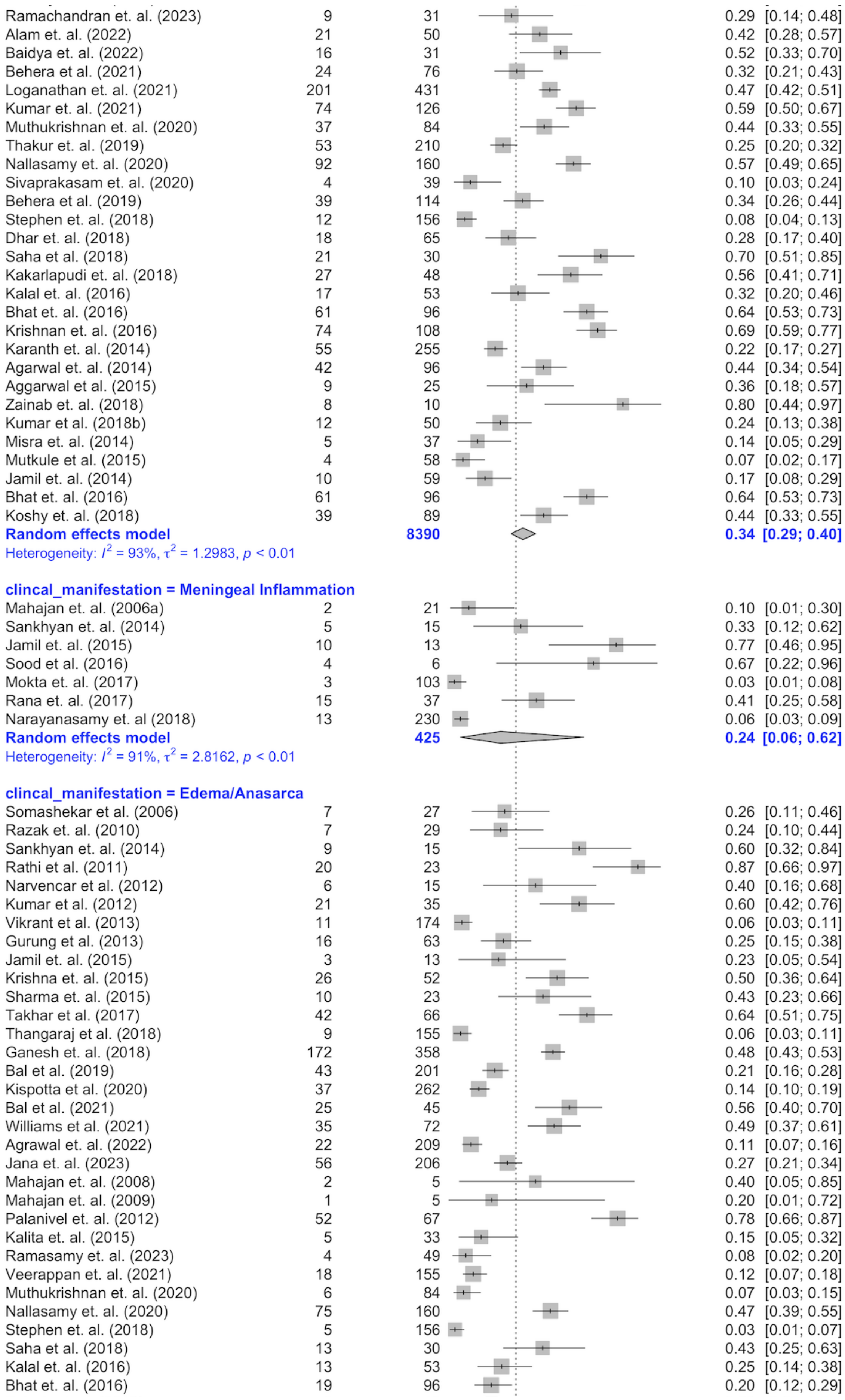

Ramachandran et. al. (2023) 9 31 0.29 [0.14; 0.48]
Alam et. al. (2022) 21 50 0.42 [0.28; 0.57]
Baidya et. al. (2022) 16 31 0.52 [0.33; 0.70]
Behera et al. (2021) 24 76 0.32 [0.21; 0.43]
Loganathan et. al. (2021) 201 431 0.47 [0.42; 0.51]
Kumar et. al. (2021) 74 126 0.59 [0.50; 0.67]
Muthukrishnan et. al. (2020) 37 84 0.44 [0.33; 0.55]
Thakur et. al. (2019) 53 210 0.25 [0.20; 0.32]
Nallasamy et. al. (2020) 92 160 0.57 [0.49; 0.65]
Sivaprakasam et. al. (2020) 4 39 0.10 [0.03; 0.24]
Behera et al. (2019) 39 114 0.34 [0.26; 0.44]
Stephen et. al. (2018) 12 156 0.08 [0.04; 0.13]
Dhar et. al. (2018) 18 65 0.28 [0.17; 0.40]
Saha et al. (2018) 21 30 0.70 [0.51; 0.85]
Kakarlapudi et. al. (2018) 27 48 0.56 [0.41; 0.71]
Kalal et. al. (2016) 17 53 0.32 [0.20; 0.46]
Bhat et. al. (2016) 61 96 0.64 [0.53; 0.73]
Krishnan et. al. (2016) 74 108 0.69 [0.59; 0.77]
Karanth et. al. (2014) 55 255 0.22 [0.17; 0.27]
Agarwal et. al. (2014) 42 96 0.44 [0.34; 0.54]
Aggarwal et al. (2015) 9 25 0.36 [0.18; 0.57]
Zainab et. al. (2018) 8 10 0.80 [0.44; 0.97]
Kumar et al. (2018b) 12 50 0.24 [0.13; 0.38]
Misra et. al. (2014) 5 37 0.14 [0.05; 0.29]
Mutkule et al. (2015) 4 58 0.07 [0.02; 0.17]
Jamil et. al. (2014) 10 59 0.17 [0.08; 0.29]
Bhat et. al. (2016) 61 96 0.64 [0.53; 0.73]
Koshy et. al. (2018) 39 89 0.44 [0.33; 0.55]
Random effects model 8390 0.34 [0.29; 0.40]
Heterogeneity: I^2 = 93%, τ^2 = 1.2983, p < 0.01
clincal_manifestation = Meningeal Inflammation
Mahajan et. al. (2006a) 2 21 0.10 [0.01; 0.30]
Sankhyan et. al. (2014) 5 15 0.33 [0.12; 0.62]
Jamil et. al. (2015) 10 13 0.77 [0.46; 0.95]
Sood et al. (2016) 4 6 0.67 [0.22; 0.96]
Mokta et. al. (2017) 3 103 0.03 [0.01; 0.08]
Rana et. al. (2017) 15 37 0.41 [0.25; 0.58]
Narayanasamy et. al (2018) 13 230 0.06 [0.03; 0.09]
Random effects model 425 0.24 [0.06; 0.62]
Heterogeneity: I^2 = 91%, τ^2 = 2.8162, p < 0.01
clincal_manifestation = Edema/Anasarca
Somashekar et al. (2006) 7 27 0.26 [0.11; 0.46]
Razak et. al. (2010) 7 29 0.24 [0.10; 0.44]
Sankhyan et. al. (2014) 9 15 0.60 [0.32; 0.84]
Rathi et al. (2011) 20 23 0.87 [0.66; 0.97]
Narvencar et al. (2012) 6 15 0.40 [0.16; 0.68]
Kumar et al. (2012) 21 35 0.60 [0.42; 0.76]
Vikrant et al. (2013) 11 174 0.06 [0.03; 0.11]
Gurung et al. (2013) 16 63 0.25 [0.15; 0.38]
Jamil et. al. (2015) 3 13 0.23 [0.05; 0.54]
Krishna et. al. (2015) 26 52 0.50 [0.36; 0.64]
Sharma et. al. (2015) 10 23 0.43 [0.23; 0.66]
Takhar et al. (2017) 42 66 0.64 [0.51; 0.75]
Thangaraj et al. (2018) 9 155 0.06 [0.03; 0.11]
Ganesh et. al. (2018) 172 358 0.48 [0.43; 0.53]
Bal et al. (2019) 43 201 0.21 [0.16; 0.28]
Kispotta et al. (2020) 37 262 0.14 [0.10; 0.19]
Bal et al. (2021) 25 45 0.56 [0.40; 0.70]
Williams et al. (2021) 35 72 0.49 [0.37; 0.61]
Agrawal et. al. (2022) 22 209 0.11 [0.07; 0.16]
Jana et. al. (2023) 56 206 0.27 [0.21; 0.34]
Mahajan et. al. (2008) 2 5 0.40 [0.05; 0.85]
Mahajan et. al. (2009) 1 5 0.20 [0.01; 0.72]
Palanivel et. al. (2012) 52 67 0.78 [0.66; 0.87]
Kalita et. al. (2015) 5 33 0.15 [0.05; 0.32]
Ramasamy et. al. (2023) 4 49 0.08 [0.02; 0.20]
Veerappan et. al. (2021) 18 155 0.12 [0.07; 0.18]
Muthukrishnan et. al. (2020) 6 84 0.07 [0.03; 0.15]
Nallasamy et. al. (2020) 75 160 0.47 [0.39; 0.55]
Stephen et. al. (2018) 5 156 0.03 [0.01; 0.07]
Saha et al. (2018) 13 30 0.43 [0.25; 0.63]
Kalal et. al. (2016) 13 53 0.25 [0.14; 0.38]
Bhat et. al. (2016) 19 96 0.20 [0.12; 0.29]

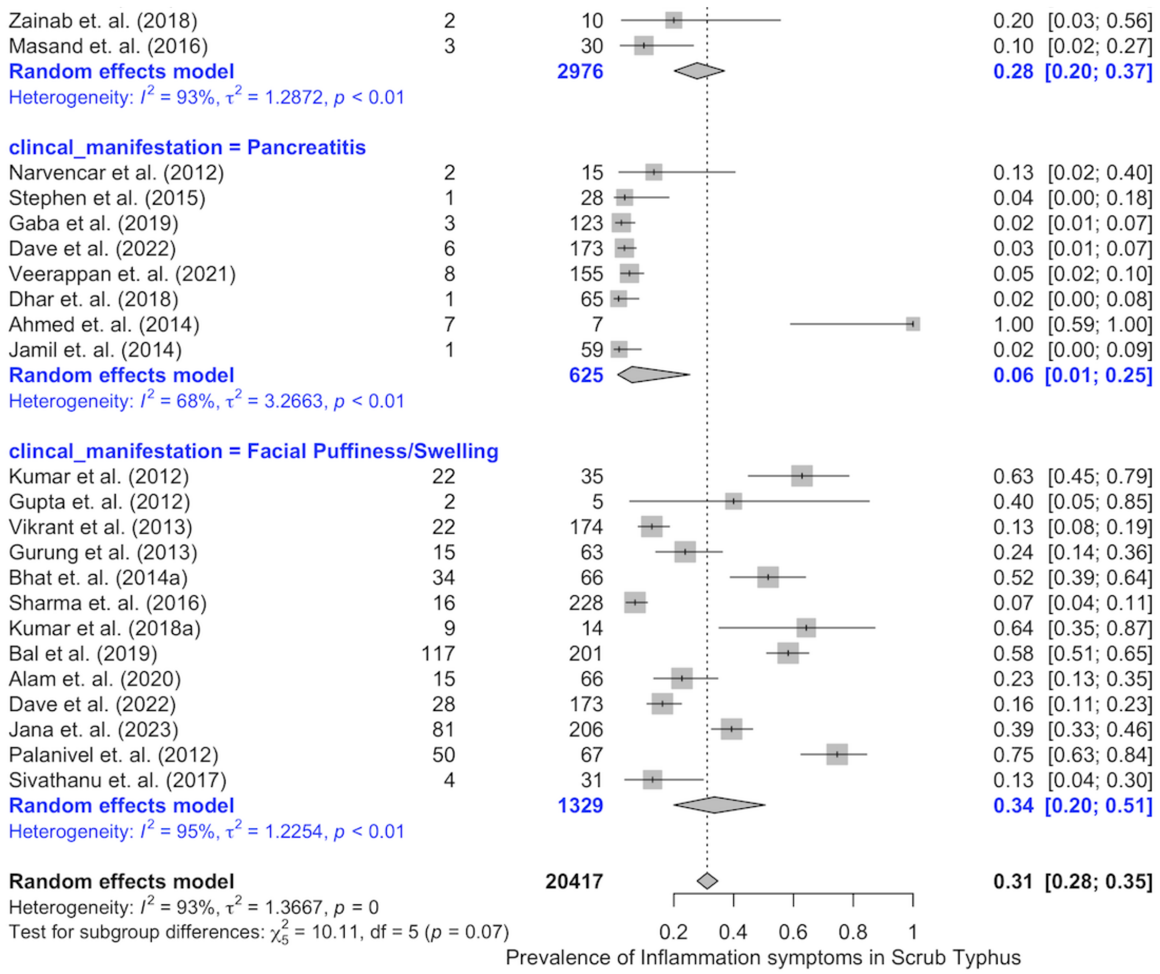

Forest plot 8. Pooled prevalence of different types of inflammation symptoms of scrub typhus

clincal_manifestation = Cough/Sore Throat

Study	No. with symtom	Total Patients	Prevalence	95% CI
Mathai et al. (2003)	12	28	0.43	[0.24; 0.63]
Mathai et al. (2003b)	1	5	0.20	[0.01; 0.72]
Mahajan et. al. (2006a)	4	21	0.19	[0.05; 0.42]
Varghese et al. (2006)	12	51	0.24	[0.13; 0.37]
Somashekar et al. (2006)	10	27	0.37	[0.19; 0.58]
Vivekanandan et. al. (2010)	20	50	0.40	[0.26; 0.55]
Razak et. al. (2010)	6	29	0.21	[0.08; 0.40]
Chrispal et. al. (2010)	57	189	0.30	[0.24; 0.37]
Dass et. al. (2011)	9	24	0.38	[0.19; 0.59]
Narvencar et al. (2012)	7	15	0.47	[0.21; 0.73]
Kumar et al. (2012)	18	35	0.51	[0.34; 0.69]
Gupta et. al. (2012)	1	5	0.20	[0.01; 0.72]
Vikrant et al. (2013)	40	174	0.23	[0.17; 0.30]
Varghese et al. (2013)	57	154	0.37	[0.29; 0.45]
Sinha et al. (2014)	42	42	1.00	[0.92; 1.00]
Poomalar & Rekha (2014)	1	8	0.12	[0.00; 0.53]
Bhat et. al. (2014a)	23	66	0.35	[0.24; 0.48]
Subbalaxmi et. al. (2014)	94	174	0.54	[0.46; 0.62]
Stephen et al. (2015)	11	28	0.39	[0.22; 0.59]
Sharma et. al. (2015)	8	23	0.35	[0.16; 0.57]
Narayanasamy et al. (2016)	96	117	0.82	[0.74; 0.89]
Arun Babu et al. (2017)	74	107	0.69	[0.59; 0.78]
Sivarajan et al. (2016)	21	90	0.23	[0.15; 0.33]
Bhargava et. al. (2016)	89	284	0.31	[0.26; 0.37]
Mahajan et. al. (2016a)	41	253	0.16	[0.12; 0.21]
Sharma et. al. (2016)	34	228	0.15	[0.11; 0.20]
Anithraj et. al. (2016)	82	140	0.59	[0.50; 0.67]
Meena et. al. (2015)	6	6	1.00	[0.54; 1.00]
Takhar et al. (2017)	32	66	0.48	[0.36; 0.61]
Thangaraj et al. (2017)	9	40	0.23	[0.11; 0.38]
Thangaraj et al. (2018)	45	155	0.29	[0.22; 0.37]
Rana et. al. (2017)	2	37	0.05	[0.01; 0.18]
Ganesh et. al. (2018)	98	358	0.27	[0.23; 0.32]
Rawat et al. (2018)	86	168	0.51	[0.43; 0.59]
Rawat et al. (2018)	38	168	0.23	[0.17; 0.30]
Kumar et al. (2018a)	12	14	0.86	[0.57; 0.98]
Bal et al. (2019)	147	201	0.73	[0.66; 0.79]
Jain et al. (2019)	33	39	0.85	[0.69; 0.94]
Vikram et al. (2020)	7	35	0.20	[0.08; 0.37]
Bhise et al. (2020)	12	158	0.08	[0.04; 0.13]
Bhattacharya et. al. (2020)	14	78	0.18	[0.10; 0.28]
Lakshmi et al. (2020)	8	89	0.09	[0.04; 0.17]
Bal et al. (2021)	11	45	0.24	[0.13; 0.40]
Paulraj et al. (2021)	9	13	0.69	[0.39; 0.91]
Bansod et. al. (2021)	54	140	0.39	[0.30; 0.47]
Agrawal et. al. (2022)	71	209	0.34	[0.28; 0.41]
Dave et al. (2022)	35	173	0.20	[0.15; 0.27]
Husain et. al. (2022)	20	361	0.06	[0.03; 0.08]
Yadav et al. (2023)	14	27	0.52	[0.32; 0.71]
Jana et. al. (2023)	45	206	0.22	[0.16; 0.28]
Narayanasamy et. al (2023)	436	560	0.78	[0.74; 0.81]
Mahajan et. al. (2009)	1	5	0.20	[0.01; 0.72]
Chrispal et al. (2010)	57	189	0.30	[0.24; 0.37]
Palanivel et. al. (2012)	49	67	0.73	[0.61; 0.83]
Varghese et. al. (2013)	3	16	0.19	[0.04; 0.46]
Varghese et. al. (2014)	237	623	0.38	[0.34; 0.42]
Ramasamy et. al. (2023)	11	49	0.22	[0.12; 0.37]
Chunduru et. al. (2023)	51	214	0.24	[0.18; 0.30]
Sultan et. al. (2022)	46	112	0.41	[0.32; 0.51]
Loganathan et. al. (2021)	89	431	0.21	[0.17; 0.25]
Thakur et. al. (2019)	46	210	0.22	[0.17; 0.28]
Sivaprakasam et. al. (2020)	12	39	0.31	[0.17; 0.48]
Basu et al. (2019)	24	61	0.39	[0.27; 0.53]
Behera et al. (2019)	27	114	0.24	[0.16; 0.33]
Stephen et. al. (2018)	74	156	0.47	[0.39; 0.56]
Dhar et. al. (2018)	23	65	0.35	[0.24; 0.48]
Bhat et. al. (2016)	18	96	0.19	[0.12; 0.28]
Karanth et. al. (2014)	64	255	0.25	[0.20; 0.31]
Agarwal et. al. (2014)	49	96	0.51	[0.41; 0.61]
Zainab et. al. (2018)	5	10	0.50	[0.19; 0.81]
Sivathanu et. al. (2017)	16	31	0.52	[0.33; 0.70]
Mutkule et al. (2015)	33	58	0.57	[0.43; 0.70]

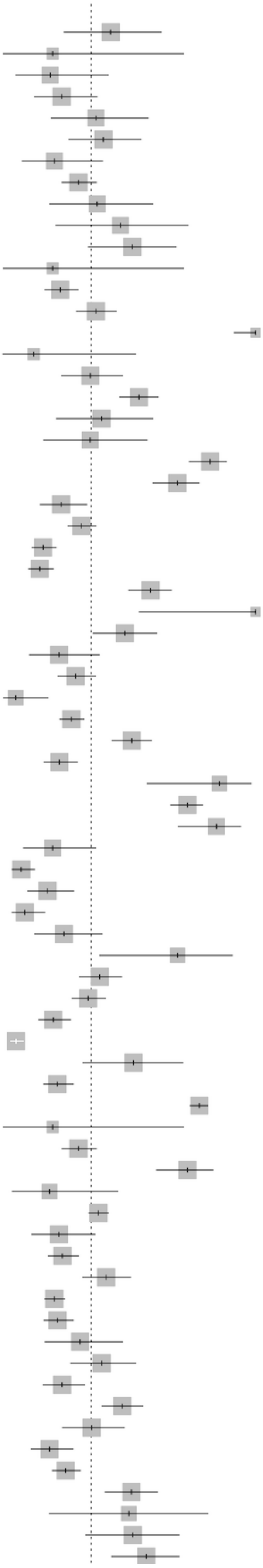

Bhat et. al. (2014b)	15	62	0.24	[0.14; 0.37]
Jamil et. al. (2014)	29	59	0.49	[0.36; 0.63]
Bhat et. al. (2016)	18	96	0.19	[0.12; 0.28]
Sugumar et. al. (2020)	2	24	0.08	[0.01; 0.27]
Rose et. al. (2017)	89	427	0.21	[0.17; 0.25]
Ganesh et. al. (2018)	98	358	0.27	[0.23; 0.32]
Basu et al. (2019)	24	61	0.39	[0.27; 0.53]
Karthika et. al. (2021)	48	124	0.39	[0.30; 0.48]
**Random effects model**		**9821**	**0.34**	**[0.29; 0.40]**
Heterogeneity: $I^2 = 94\%$, $\tau^2 = 0.8864$, $p < 0.01$				
**clincal_manifestation = Breathing problems**				
Mathai et al. (2003b)	1	5	0.20	[0.01; 0.72]
Vivekanandan et. al. (2010)	13	50	0.26	[0.15; 0.40]
Ahmad et. al. (2010)	3	9	0.33	[0.07; 0.70]
Razak et. al. (2010)	4	29	0.14	[0.04; 0.32]
Chrispal et. al. (2010)	71	189	0.38	[0.31; 0.45]
Narvencar et al. (2012)	9	15	0.60	[0.32; 0.84]
Kumar et al. (2012)	13	35	0.37	[0.21; 0.55]
Gupta et. al. (2012)	1	5	0.20	[0.01; 0.72]
Vikrant et al. (2013)	40	174	0.23	[0.17; 0.30]
Viswanathan et. al. (2013)	25	65	0.38	[0.27; 0.51]
Varghese et al. (2013)	110	154	0.71	[0.64; 0.78]
Sinha et al. (2014)	35	42	0.83	[0.69; 0.93]
Poomalar & Rekha (2014)	2	8	0.25	[0.03; 0.65]
Sethi et. al. (2014)	16	45	0.36	[0.22; 0.51]
Bhat et. al. (2014a)	19	66	0.29	[0.18; 0.41]
Subbalaxmi et. al. (2014)	84	174	0.48	[0.41; 0.56]
Kumar et al. (2014)	33	49	0.67	[0.52; 0.80]
Kumar et al. (2015)	5	14	0.36	[0.13; 0.65]
Sharma et. al. (2015)	7	23	0.30	[0.13; 0.53]
Narayanasamy et al. (2016)	31	117	0.26	[0.19; 0.35]
Bhargava et. al. (2016)	66	284	0.23	[0.18; 0.29]
Ahmad et al. (2016)	19	65	0.29	[0.19; 0.42]
Sharma et. al. (2016)	95	228	0.42	[0.35; 0.48]
Takhar et al. (2017)	44	66	0.67	[0.54; 0.78]
Mokta et. al. (2017)	53	103	0.51	[0.41; 0.61]
Rana et. al. (2017)	4	37	0.11	[0.03; 0.25]
Gaba et al. (2019)	44	123	0.36	[0.27; 0.45]
Bhise et al. (2020)	15	158	0.09	[0.05; 0.15]
Bhattacharya et. al. (2020)	24	78	0.31	[0.21; 0.42]
Alam et. al. (2020)	36	66	0.55	[0.42; 0.67]
Kispotta et al. (2020)	12	262	0.05	[0.02; 0.08]
Bal et al. (2021)	24	45	0.53	[0.38; 0.68]
Williams et al. (2021)	27	72	0.38	[0.26; 0.50]
Verma et. al. (2021)	10	52	0.19	[0.10; 0.33]
Basu et al. (2021)	9	18	0.50	[0.26; 0.74]
Behara et. al. (2021)	16	34	0.47	[0.30; 0.65]
Khemka et al. (2021)	42	62	0.68	[0.55; 0.79]
Bansod et. al. (2021)	39	140	0.28	[0.21; 0.36]
Dave et al. (2022)	49	173	0.28	[0.22; 0.36]
John et. al. (2023)	86	198	0.43	[0.36; 0.51]
Yadav et al. (2023)	9	27	0.33	[0.17; 0.54]
Damodar et. al. (2023)	8	87	0.09	[0.04; 0.17]
Varghese et. al. (2013)	4	16	0.25	[0.07; 0.52]
Peter et. al. (2013)	82	84	0.98	[0.92; 1.00]
Varghese et. al. (2014)	303	623	0.49	[0.45; 0.53]
Ramasamy et. al. (2023)	20	49	0.41	[0.27; 0.56]
Ramasamy et. al. (2023)	9	49	0.18	[0.09; 0.32]
Alam et. al. (2022)	41	50	0.82	[0.69; 0.91]
Sardana et. al. (2020)	2	5	0.40	[0.05; 0.85]
Rama et. al. (2019)	2	5	0.40	[0.05; 0.85]
Chunduru et. al. (2023)	43	214	0.20	[0.15; 0.26]
Sultan et. al. (2022)	49	112	0.44	[0.34; 0.53]
Loganathan et. al. (2021)	77	431	0.18	[0.14; 0.22]
Kumar et. al. (2021)	100	126	0.79	[0.71; 0.86]
Nallasamy et. al. (2020)	67	160	0.42	[0.34; 0.50]
Dhar et. al. (2018)	6	65	0.09	[0.03; 0.19]
Kakarlapudi et. al. (2018)	1	48	0.02	[0.00; 0.11]
Bhat et. al. (2016)	25	96	0.26	[0.18; 0.36]
Karanth et. al. (2014)	52	255	0.20	[0.16; 0.26]
Aggarwal et al. (2015)	12	25	0.48	[0.28; 0.69]
Misra et. al. (2014)	30	37	0.81	[0.65; 0.92]
Bhat et. al. (2014b)	16	62	0.26	[0.16; 0.38]
Bhat et. al. (2016)	25	96	0.26	[0.18; 0.36]

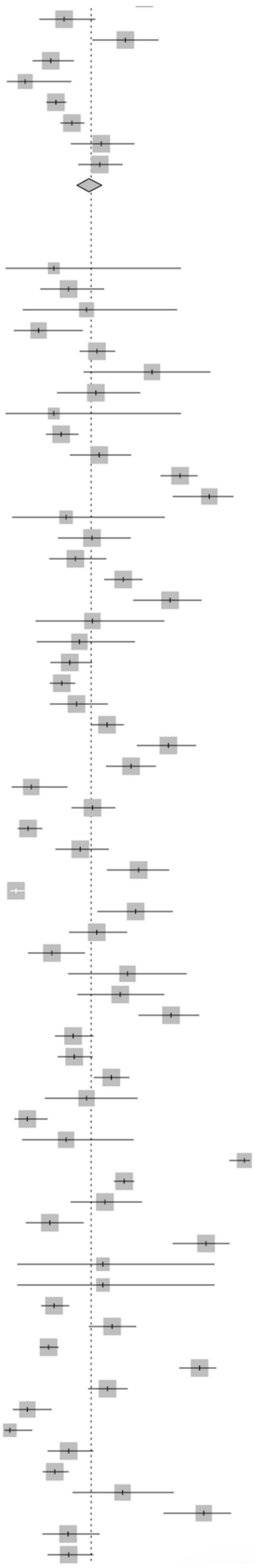

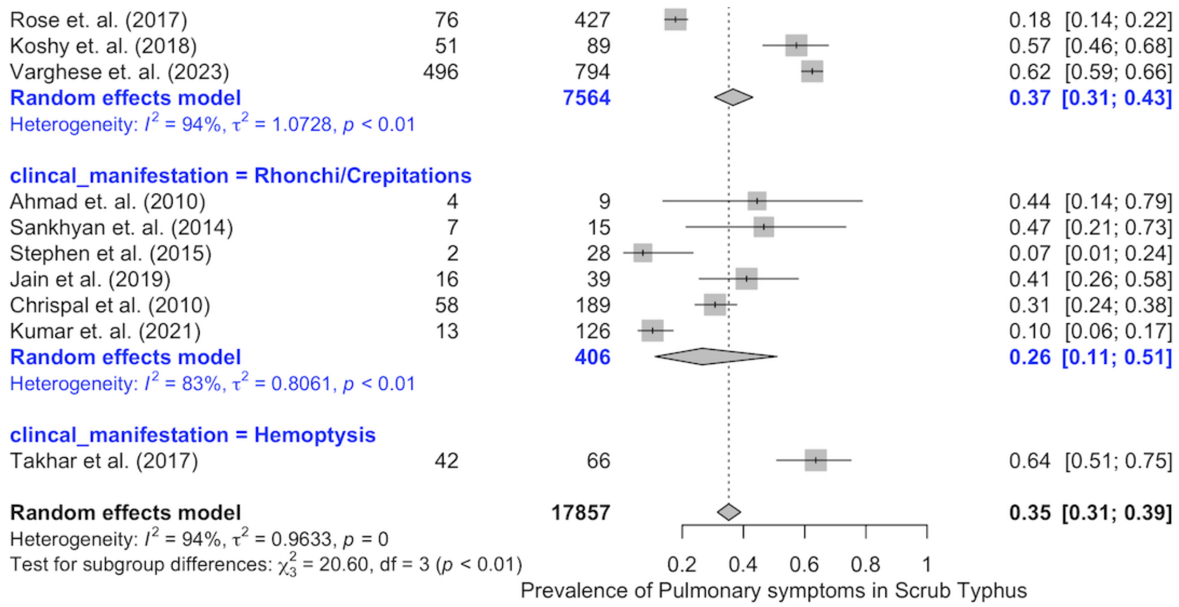

Forest plot 9. Pooled prevalence of different types of pulmonary symptoms of scrub typhus

Study	No. with symtom	Total Patients	Prevalence	95% CI
**clincal_manifestation = Respiratory Tract Infection**				
Isaac et al. (2004)	1	8	0.12	[0.00; 0.53]
Vikrant et al. (2013)	19	174	0.11	[0.07; 0.17]
Jana et. al. (2023)	47	206	0.23	[0.17; 0.29]
**Random effects model**		**388**	**0.16**	**[0.05; 0.42]**
Heterogeneity: $I^2 = 78\%$, $\tau^2 = 0.1578$, $p = 0.01$				
**clincal_manifestation = Pneumonia**				
Sharma et al. (2005)	15	45	0.33	[0.20; 0.49]
Kumar et al. (2012)	1	35	0.03	[0.00; 0.15]
Vikrant et al. (2013)	24	174	0.14	[0.09; 0.20]
Bhat et. al. (2014a)	7	66	0.11	[0.04; 0.21]
Sivarajan et al. (2016)	14	90	0.16	[0.09; 0.25]
Anithraj et. al. (2016)	11	140	0.08	[0.04; 0.14]
Meena et. al. (2015)	1	6	0.17	[0.00; 0.64]
Rawat et al. (2018)	9	168	0.05	[0.02; 0.10]
Lakshmi et al. (2020)	1	89	0.01	[0.00; 0.06]
Verma et. al. (2021)	6	52	0.12	[0.04; 0.23]
Behara et. al. (2021)	18	34	0.53	[0.35; 0.70]
Agrawal et. al. (2022)	43	209	0.21	[0.15; 0.27]
Alam et. al. (2022)	14	50	0.28	[0.16; 0.42]
Sivaprakasam et. al. (2020)	2	39	0.05	[0.01; 0.17]
Basu et al. (2019)	6	61	0.10	[0.04; 0.20]
Stephen et. al. (2018)	13	156	0.08	[0.05; 0.14]
Dhar et. al. (2018)	7	65	0.11	[0.04; 0.21]
Bhat et. al. (2016)	1	96	0.01	[0.00; 0.06]
Bhat et. al. (2014b)	6	62	0.10	[0.04; 0.20]
Veerappan et. al. (2021)	8	155	0.05	[0.02; 0.10]
**Random effects model**		**1792**	**0.11**	**[0.07; 0.17]**
Heterogeneity: $I^2 = 83\%$, $\tau^2 = 0.8484$, $p < 0.01$				
**clincal_manifestation = Acute Respiratory Distress Syndrome**				
Mahajan et. al. (2006a)	2	21	0.10	[0.01; 0.30]
Narvencar et al. (2012)	9	15	0.60	[0.32; 0.84]
Kumar et al. (2012)	3	35	0.09	[0.02; 0.23]
Vikrant et al. (2013)	20	174	0.11	[0.07; 0.17]
Varghese et al. (2013)	67	154	0.44	[0.36; 0.52]
Bhat et. al. (2014a)	16	66	0.24	[0.15; 0.36]
Subbalaxmi et. al. (2014)	11	174	0.06	[0.03; 0.11]
Bhargava et. al. (2016)	17	284	0.06	[0.04; 0.09]
Basheer et. al. (2016)	1	6	0.17	[0.00; 0.64]
Sharma et. al. (2016)	57	228	0.25	[0.20; 0.31]
Takhar et al. (2017)	34	66	0.52	[0.39; 0.64]
Ganesh et. al. (2018)	11	358	0.03	[0.02; 0.05]
Rawat et al. (2018)	3	168	0.02	[0.00; 0.05]
Bal et al. (2019)	6	201	0.03	[0.01; 0.06]
Gaba et al. (2019)	32	123	0.26	[0.19; 0.35]
Jain et al. (2019)	28	39	0.72	[0.55; 0.85]
Bhise et al. (2020)	11	158	0.07	[0.04; 0.12]
Lakshmi et al. (2020)	1	89	0.01	[0.00; 0.06]
Kispotta et al. (2020)	2	262	0.01	[0.00; 0.03]
Bal et al. (2021)	8	45	0.18	[0.08; 0.32]
Williams et al. (2021)	23	72	0.32	[0.21; 0.44]
Verma et. al. (2021)	4	52	0.08	[0.02; 0.19]
Basu et al. (2021)	8	18	0.44	[0.22; 0.69]
Behara et. al. (2021)	4	34	0.12	[0.03; 0.27]
Khemka et al. (2021)	6	62	0.10	[0.04; 0.20]
Dave et al. (2022)	60	173	0.35	[0.28; 0.42]
Roychowdhary et. al. (2022)	10	105	0.10	[0.05; 0.17]
John et. al. (2023)	99	198	0.50	[0.43; 0.57]
Yadav et al. (2023)	1	27	0.04	[0.00; 0.19]
Jana et. al. (2023)	47	206	0.23	[0.17; 0.29]
Chrispal et al. (2010)	47	189	0.25	[0.19; 0.32]
Palanivel et. al. (2012)	3	67	0.04	[0.01; 0.13]
Mittal et al. (2012)	2	90	0.02	[0.00; 0.08]
Varghese et. al. (2014)	210	623	0.34	[0.30; 0.38]
Oberoi & Varghese (2014)	31	98	0.32	[0.23; 0.42]
Ramachandran et. al. (2023)	2	31	0.06	[0.01; 0.21]
Rama et. al. (2019)	4	5	0.80	[0.28; 0.99]
Loganathan et. al. (2021)	65	431	0.15	[0.12; 0.19]
Muthukrishnan et. al. (2020)	10	84	0.12	[0.06; 0.21]
Muthukrishnan et. al. (2020)	7	84	0.08	[0.03; 0.16]

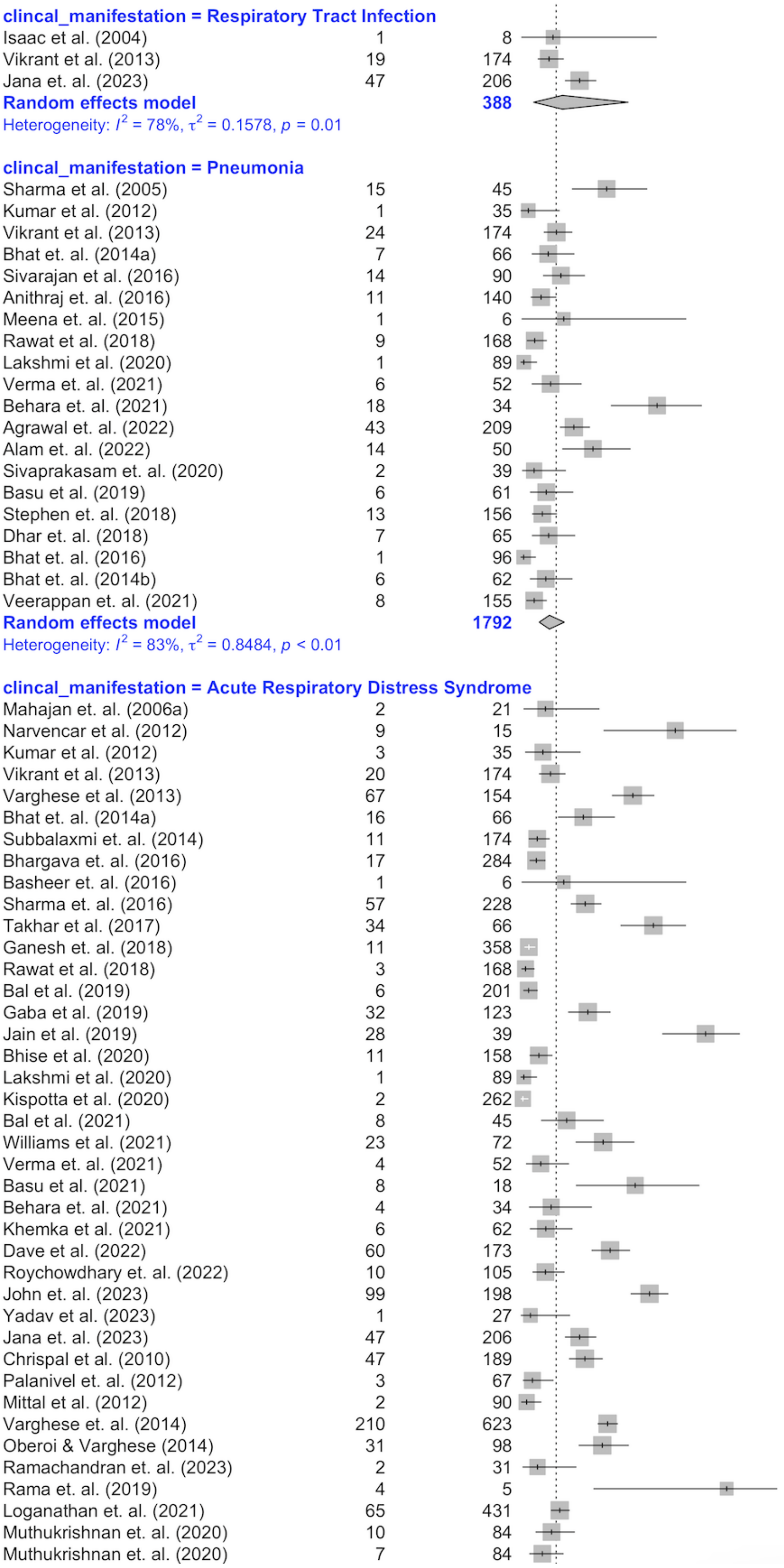

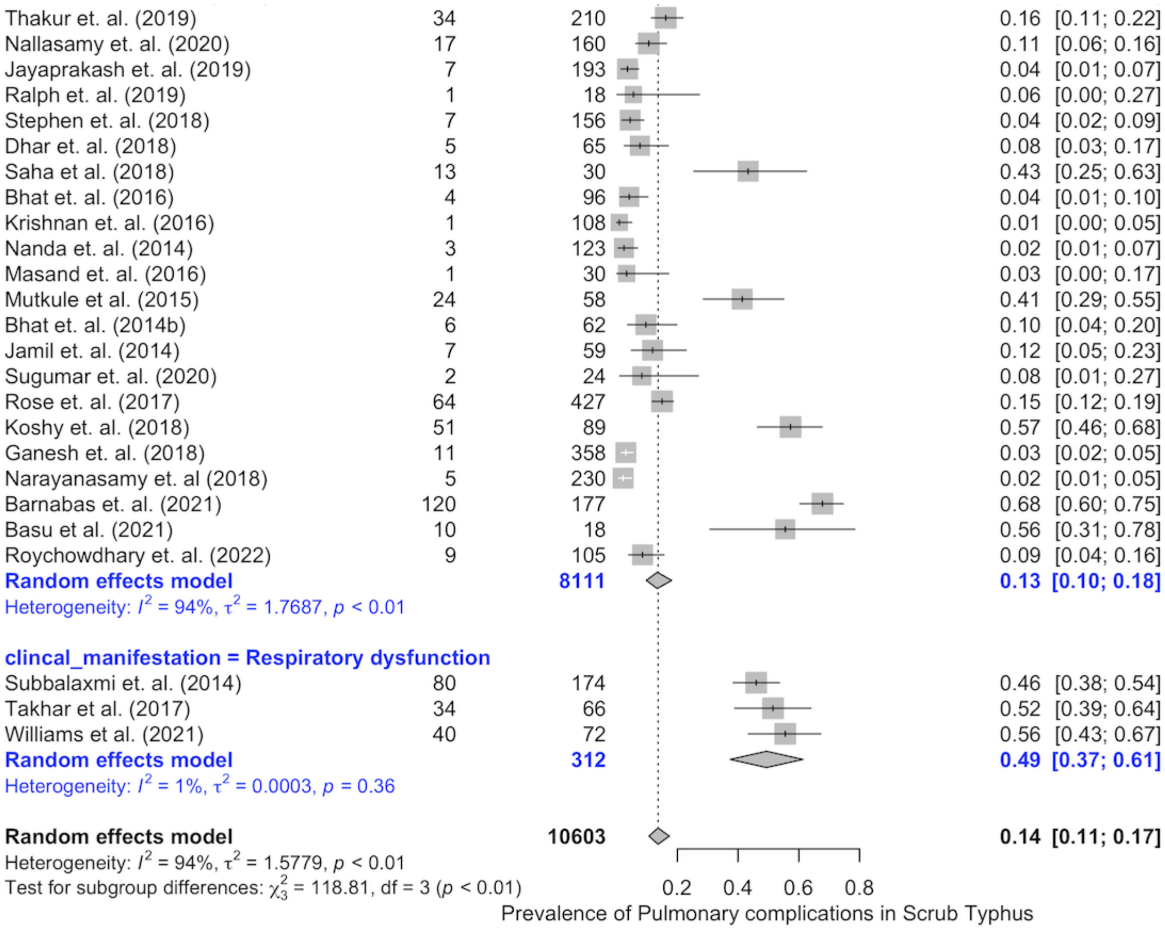

Forest plot 10. Pooled prevalence of different types of pulmonary complications of scrub typhus

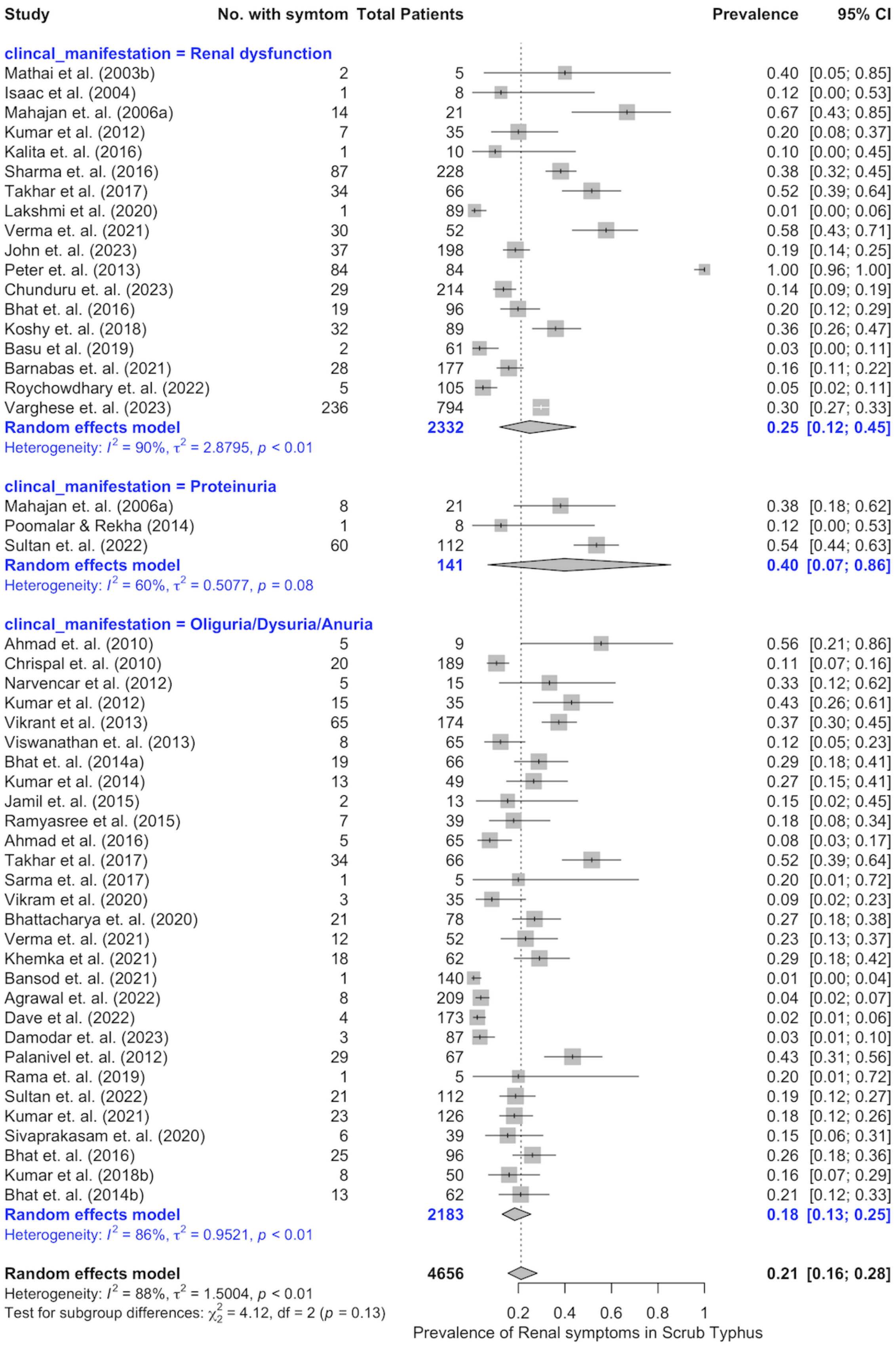

Forest plot 11. Pooled prevalence of different types of renal symptoms of scrub typhus

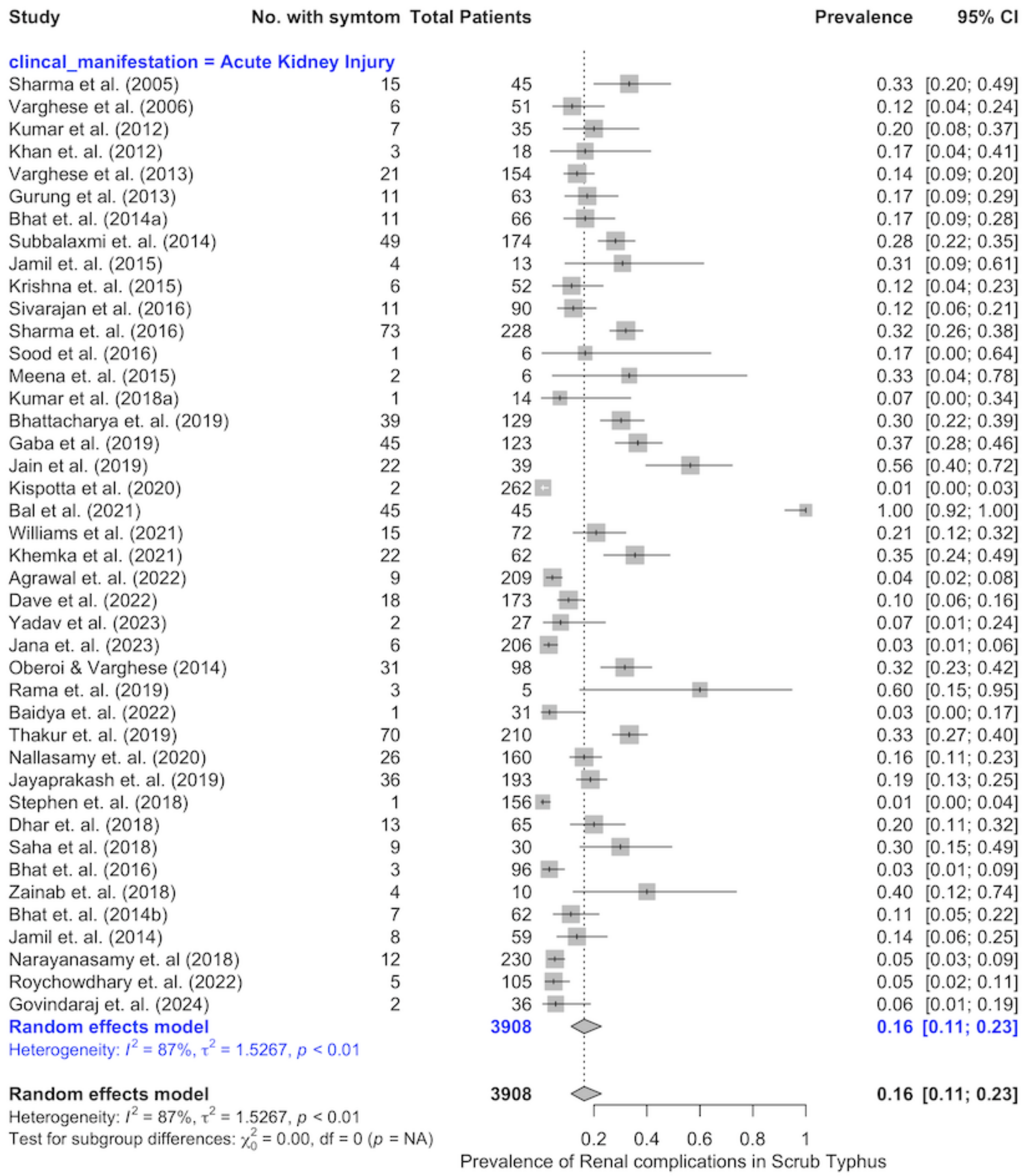

Forest plot 12. Pooled prevalence of different types of renal complications of scrub typhus

Study	No. with symtom	Total Patients	Prevalence	95% CI
**clincal_manifestation = Altered sensorium**				
Mathai et al. (2003)	5	28	0.18	[0.06; 0.37]
Mahajan et. al. (2006a)	5	21	0.24	[0.08; 0.47]
Varghese et al. (2006)	9	51	0.18	[0.08; 0.31]
Vivekanandan et. al. (2010)	10	50	0.20	[0.10; 0.34]
Razak et. al. (2010)	6	29	0.21	[0.08; 0.40]
Chrispal et. al. (2010)	42	189	0.22	[0.17; 0.29]
Mahajan et. al. (2010)	4	4	1.00	[0.40; 1.00]
Dass et. al. (2011)	4	24	0.17	[0.05; 0.37]
Narvencar et al. (2012)	1	15	0.07	[0.00; 0.32]
Kumar et al. (2012)	6	35	0.17	[0.07; 0.34]
Gupta et. al. (2012)	1	5	0.20	[0.01; 0.72]
Vikrant et al. (2013)	28	174	0.16	[0.11; 0.22]
Varghese et al. (2013)	38	154	0.25	[0.18; 0.32]
Sinha et al. (2014)	8	42	0.19	[0.09; 0.34]
Sethi et. al. (2014)	10	45	0.22	[0.11; 0.37]
Bhat et. al. (2014a)	15	66	0.23	[0.13; 0.35]
Subbalaxmi et. al. (2014)	43	174	0.25	[0.18; 0.32]
Kumar et al. (2014)	12	49	0.24	[0.13; 0.39]
Abhilash et. al. (2015)	102	189	0.54	[0.47; 0.61]
Stephen et al. (2015)	3	28	0.11	[0.02; 0.28]
Jamil et. al. (2015)	13	13	1.00	[0.75; 1.00]
Krishna et. al. (2015)	4	52	0.08	[0.02; 0.19]
Kumar et al. (2015)	1	14	0.07	[0.00; 0.34]
Sharma et. al. (2015)	14	23	0.61	[0.39; 0.80]
Narayanasamy et al. (2016)	29	117	0.25	[0.17; 0.34]
Rajan et al. (2016)	4	33	0.12	[0.03; 0.28]
Bhargava et. al. (2016)	28	284	0.10	[0.07; 0.14]
Mahajan et. al. (2016a)	41	253	0.16	[0.12; 0.21]
Sharma et. al. (2016)	55	228	0.24	[0.19; 0.30]
Sood et al. (2016)	6	6	1.00	[0.54; 1.00]
Takhar et al. (2017)	26	66	0.39	[0.28; 0.52]
Sarma et. al. (2017)	1	5	0.20	[0.01; 0.72]
Rana et. al. (2017)	31	37	0.84	[0.68; 0.94]
Rawat et al. (2018)	7	168	0.04	[0.02; 0.08]
Kumar et al. (2018a)	14	14	1.00	[0.77; 1.00]
Gaba et al. (2019)	25	123	0.20	[0.14; 0.29]
Jain et al. (2019)	15	39	0.38	[0.23; 0.55]
Bhise et al. (2020)	4	158	0.03	[0.01; 0.06]
Bhattacharya et. al. (2020)	29	78	0.37	[0.26; 0.49]
Lakshmi et al. (2020)	5	89	0.06	[0.02; 0.13]
Kaur et. al. (2020)	8	8	1.00	[0.63; 1.00]
Williams et al. (2021)	16	72	0.22	[0.13; 0.34]
Verma et. al. (2021)	27	52	0.52	[0.38; 0.66]
Das et. al. (2021)	92	92	1.00	[0.96; 1.00]
Khan et. al. (2017)	104	104	1.00	[0.97; 1.00]
Basu et al. (2021)	3	18	0.17	[0.04; 0.41]
Khemka et al. (2021)	41	62	0.66	[0.53; 0.78]
Bansod et. al. (2021)	13	140	0.09	[0.05; 0.15]
Agrawal et. al. (2022)	20	209	0.10	[0.06; 0.14]
Dave et al. (2022)	28	173	0.16	[0.11; 0.23]
John et. al. (2023)	20	198	0.10	[0.06; 0.15]
Damodar et. al. (2023)	87	87	1.00	[0.96; 1.00]
Mahajan et. al. (2006b)	5	21	0.24	[0.08; 0.47]
Mahajan et. al. (2008)	2	5	0.40	[0.05; 0.85]
Mahajan et. al. (2009)	1	5	0.20	[0.01; 0.72]
Palanivel et. al. (2012)	39	67	0.58	[0.46; 0.70]
Varghese et. al. (2013)	9	16	0.56	[0.30; 0.80]
Peter et. al. (2013)	80	84	0.95	[0.88; 0.99]
Varghese et. al. (2014)	161	623	0.26	[0.22; 0.29]
Kalita et. al. (2015)	13	33	0.39	[0.23; 0.58]
Boorugu et al. (2014)	42	189	0.22	[0.17; 0.29]
Ramasamy et. al. (2023)	5	49	0.10	[0.03; 0.22]
Shiva et. al. (2022)	7	11	0.64	[0.31; 0.89]
Sardana et. al. (2020)	2	5	0.40	[0.05; 0.85]
Rama et. al. (2019)	3	5	0.60	[0.15; 0.95]
Chunduru et. al. (2023)	17	214	0.08	[0.05; 0.12]
Vikas et. al. (2023)	8	8	1.00	[0.63; 1.00]
Baidya et. al. (2022)	15	31	0.48	[0.30; 0.67]
Kumar et. al. (2021)	37	126	0.29	[0.22; 0.38]
Thakur et. al. (2019)	60	210	0.29	[0.23; 0.35]
Nallasamy et. al. (2020)	39	160	0.24	[0.18; 0.32]
Sivaprakasam et. al. (2020)	4	39	0.10	[0.03; 0.24]
Ralph et. al. (2019)	7	18	0.39	[0.17; 0.64]
[illegible]	[illegible]	[illegible]	[illegible]	[illegible]

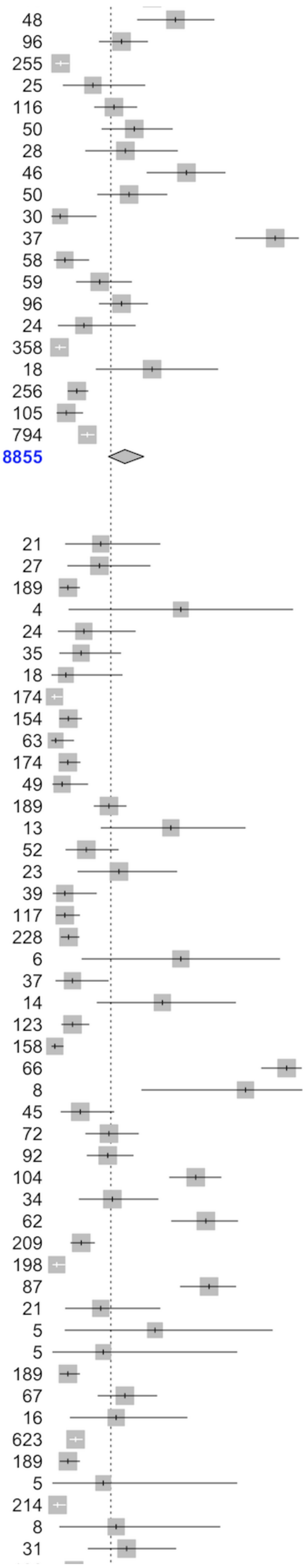

Kakarlapudi et. al. (2018)	23	48	0.48	[0.33; 0.63]
Bhat et. al. (2016)	26	96	0.27	[0.19; 0.37]
Karanth et. al. (2014)	9	255	0.04	[0.02; 0.07]
Aggarwal et al. (2015)	4	25	0.16	[0.05; 0.36]
Pichamuthu et al. (2014)	28	116	0.24	[0.17; 0.33]
Kumar et al. (2018b)	16	50	0.32	[0.20; 0.47]
Valappil et. al. (2017)	8	28	0.29	[0.13; 0.49]
Mittal et. al. (2017)	24	46	0.52	[0.37; 0.67]
Balasubramanian et. al. (2016)	15	50	0.30	[0.18; 0.45]
Masand et. al. (2016)	1	30	0.03	[0.00; 0.17]
Misra et. al. (2014)	32	37	0.86	[0.71; 0.95]
Mutkule et al. (2015)	3	58	0.05	[0.01; 0.14]
Jamil et. al. (2014)	11	59	0.19	[0.10; 0.31]
Bhat et. al. (2016)	26	96	0.27	[0.19; 0.37]
Sugumar et. al. (2020)	3	24	0.12	[0.03; 0.32]
Ganesh et. al. (2018)	11	358	0.03	[0.02; 0.05]
Basu et al. (2021)	7	18	0.39	[0.17; 0.64]
Narayanasamy et. al (2021)	25	256	0.10	[0.06; 0.14]
Roychowdhary et. al. (2022)	6	105	0.06	[0.02; 0.12]
Varghese et. al. (2023)	110	794	0.14	[0.12; 0.16]
**Random effects model**		**8855**	**0.28**	**[0.22; 0.36]**
Heterogeneity: $I^2 = 90\%$, $\tau^2 = 2.3618$, $p < 0.01$				
**clincal_manifestation = Seizures/Coma/Hemiparesis**				
Mahajan et. al. (2006a)	4	21	0.19	[0.05; 0.42]
Somashekar et al. (2006)	5	27	0.19	[0.06; 0.38]
Chrispal et. al. (2010)	12	189	0.06	[0.03; 0.11]
Mahajan et. al. (2010)	2	4	0.50	[0.07; 0.93]
Dass et. al. (2011)	3	24	0.12	[0.03; 0.32]
Kumar et al. (2012)	4	35	0.11	[0.03; 0.27]
Khan et. al. (2012)	1	18	0.06	[0.00; 0.27]
Vikrant et al. (2013)	2	174	0.01	[0.00; 0.04]
Varghese et al. (2013)	10	154	0.06	[0.03; 0.12]
Gurung et al. (2013)	1	63	0.02	[0.00; 0.09]
Subbalaxmi et. al. (2014)	11	174	0.06	[0.03; 0.11]
Kumar et al. (2014)	2	49	0.04	[0.00; 0.14]
Abhilash et. al. (2015)	42	189	0.22	[0.17; 0.29]
Jamil et. al. (2015)	6	13	0.46	[0.19; 0.75]
Krishna et. al. (2015)	7	52	0.13	[0.06; 0.26]
Sharma et. al. (2015)	6	23	0.26	[0.10; 0.48]
Ramyasree et al. (2015)	2	39	0.05	[0.01; 0.17]
Narayanasamy et al. (2016)	6	117	0.05	[0.02; 0.11]
Sharma et. al. (2016)	15	228	0.07	[0.04; 0.11]
Sood et al. (2016)	3	6	0.50	[0.12; 0.88]
Rana et. al. (2017)	3	37	0.08	[0.02; 0.22]
Kumar et al. (2018a)	6	14	0.43	[0.18; 0.71]
Gaba et al. (2019)	10	123	0.08	[0.04; 0.14]
Bhise et al. (2020)	2	158	0.01	[0.00; 0.04]
Alam et. al. (2020)	60	66	0.91	[0.81; 0.97]
Kaur et. al. (2020)	6	8	0.75	[0.35; 0.97]
Bal et al. (2021)	5	45	0.11	[0.04; 0.24]
Williams et al. (2021)	16	72	0.22	[0.13; 0.34]
Das et. al. (2021)	20	92	0.22	[0.14; 0.32]
Khan et. al. (2017)	58	104	0.56	[0.46; 0.66]
Behara et. al. (2021)	8	34	0.24	[0.11; 0.41]
Khemka et al. (2021)	37	62	0.60	[0.46; 0.72]
Agrawal et. al. (2022)	24	209	0.11	[0.07; 0.17]
John et. al. (2023)	4	198	0.02	[0.01; 0.05]
Damodar et. al. (2023)	53	87	0.61	[0.50; 0.71]
Mahajan et. al. (2006b)	4	21	0.19	[0.05; 0.42]
Mahajan et. al. (2008)	2	5	0.40	[0.05; 0.85]
Mahajan et. al. (2009)	1	5	0.20	[0.01; 0.72]
Chrispal et al. (2010)	12	189	0.06	[0.03; 0.11]
Palanivel et. al. (2012)	19	67	0.28	[0.18; 0.41]
Varghese et. al. (2013)	4	16	0.25	[0.07; 0.52]
Varghese et. al. (2014)	58	623	0.09	[0.07; 0.12]
Boorugu et al. (2014)	12	189	0.06	[0.03; 0.11]
Vindhiya et. al. (2023)	1	5	0.20	[0.01; 0.72]
Chunduru et. al. (2023)	5	214	0.02	[0.01; 0.05]
Vikas et. al. (2023)	2	8	0.25	[0.03; 0.65]
Baidya et. al. (2022)	9	31	0.29	[0.14; 0.48]

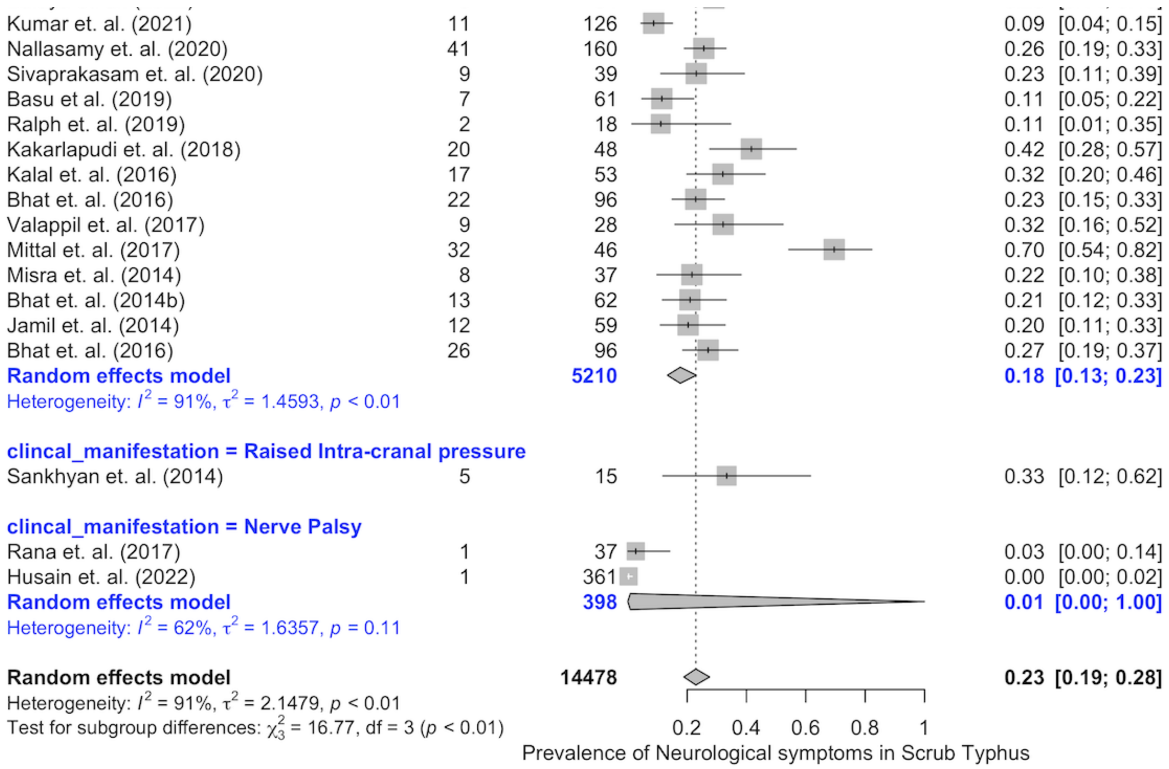

Forest plot 13. Pooled prevalence of different types of neurological symptoms of scrub typhus

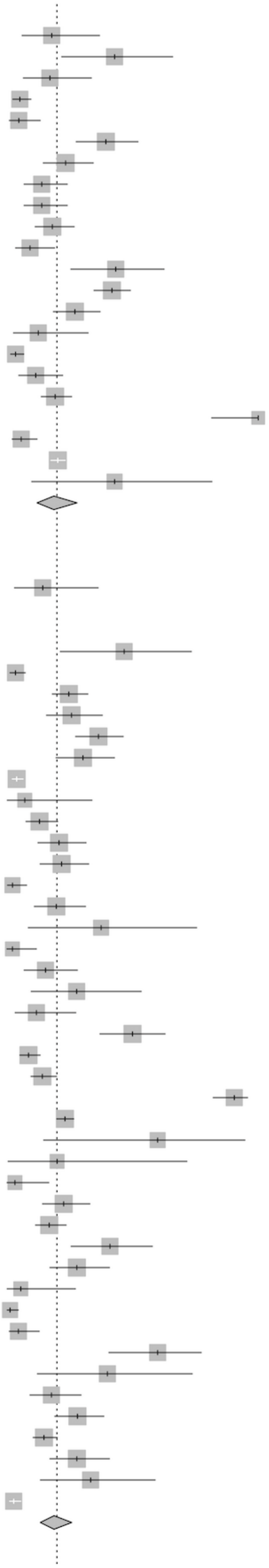

Study	No. with symtom	Total Patients	Prevalence	95% CI
**clincal_manifestation = Other Neurological Manifestations**				
Mathai et al. (2003)	5	28	0.18	[0.06; 0.37]
Mahajan et. al. (2006a)	9	21	0.43	[0.22; 0.66]
Kumar et al. (2012)	6	35	0.17	[0.07; 0.34]
Vikrant et al. (2013)	9	174	0.05	[0.02; 0.10]
Gurung et al. (2013)	3	63	0.05	[0.01; 0.13]
Takhar et al. (2017)	26	66	0.39	[0.28; 0.52]
Singla et. al. (2020)	18	77	0.23	[0.14; 0.34]
Williams et al. (2021)	10	72	0.14	[0.07; 0.24]
Williams et al. (2021)	10	72	0.14	[0.07; 0.24]
Roychowdhary et. al. (2022)	19	105	0.18	[0.11; 0.27]
Dhar et. al. (2018)	6	65	0.09	[0.03; 0.19]
Saha et al. (2018)	13	30	0.43	[0.25; 0.63]
Boorugu et al. (2014)	79	189	0.42	[0.35; 0.49]
Bhat et. al. (2016)	26	96	0.27	[0.19; 0.37]
Sugumar et. al. (2020)	3	24	0.12	[0.03; 0.32]
Narayanasamy et. al (2018)	8	230	0.03	[0.02; 0.07]
Basu et al. (2019)	7	61	0.11	[0.05; 0.22]
Barnabas et. al. (2021)	34	177	0.19	[0.14; 0.26]
Basu et al. (2021)	18	18	1.00	[0.81; 1.00]
Roychowdhary et. al. (2022)	6	105	0.06	[0.02; 0.12]
Varghese et. al. (2023)	161	794	0.20	[0.18; 0.23]
Rehani et. al. (2024)	3	7	0.43	[0.10; 0.82]
**Random effects model**		**2509**	**0.19**	**[0.12; 0.28]**
Heterogeneity: $I^2 = 88\%$, $\tau^2 = 1.1991$, $p < 0.01$				
**clincal_manifestation = Cerebrospinal fluid abnormalities**				
Mahajan et. al. (2006a)	3	21	0.14	[0.03; 0.36]
**clincal_manifestation = Shock**				
Narvencar et al. (2012)	7	15	0.47	[0.21; 0.73]
Vikrant et al. (2013)	6	174	0.03	[0.01; 0.07]
Varghese et al. (2013)	38	154	0.25	[0.18; 0.32]
Bhat et. al. (2014a)	17	66	0.26	[0.16; 0.38]
Arun Babu et al. (2017)	39	107	0.36	[0.27; 0.46]
Takhar et al. (2017)	20	66	0.30	[0.20; 0.43]
Ganesh et. al. (2018)	14	358	0.04	[0.02; 0.06]
Kumar et al. (2018a)	1	14	0.07	[0.00; 0.34]
Gaba et al. (2019)	16	123	0.13	[0.08; 0.20]
Singla et. al. (2020)	16	77	0.21	[0.12; 0.32]
Bhattacharya et. al. (2020)	17	78	0.22	[0.13; 0.33]
Lakshmi et al. (2020)	2	89	0.02	[0.00; 0.08]
Alam et. al. (2020)	13	66	0.20	[0.11; 0.31]
Kaur et. al. (2020)	3	8	0.38	[0.09; 0.76]
Bal et al. (2021)	1	45	0.02	[0.00; 0.12]
Verma et. al. (2021)	8	52	0.15	[0.07; 0.28]
Basu et al. (2021)	5	18	0.28	[0.10; 0.53]
Behara et. al. (2021)	4	34	0.12	[0.03; 0.27]
Khemka et al. (2021)	31	62	0.50	[0.37; 0.63]
Agrawal et. al. (2022)	18	209	0.09	[0.05; 0.13]
John et. al. (2023)	28	198	0.14	[0.10; 0.20]
Peter et. al. (2013)	76	84	0.90	[0.82; 0.96]
Varghese et. al. (2014)	144	623	0.23	[0.20; 0.27]
Vindhiya et. al. (2023)	3	5	0.60	[0.15; 0.95]
Rama et. al. (2019)	1	5	0.20	[0.01; 0.72]
Baidya et. al. (2022)	1	31	0.03	[0.00; 0.17]
Muthukrishnan et. al. (2020)	19	84	0.23	[0.14; 0.33]
Nallasamy et. al. (2020)	27	160	0.17	[0.11; 0.24]
Sivaprakasam et. al. (2020)	16	39	0.41	[0.26; 0.58]
Basu et al. (2019)	17	61	0.28	[0.17; 0.41]
Ralph et. al. (2019)	1	18	0.06	[0.00; 0.27]
Stephen et. al. (2018)	2	156	0.01	[0.00; 0.05]
Dhar et. al. (2018)	3	65	0.05	[0.01; 0.13]
Saha et al. (2018)	18	30	0.60	[0.41; 0.77]
Zainab et. al. (2018)	4	10	0.40	[0.12; 0.74]
Bhat et. al. (2014b)	11	62	0.18	[0.09; 0.30]
Koshy et. al. (2018)	25	89	0.28	[0.19; 0.39]
Narayanasamy et. al (2018)	34	230	0.15	[0.10; 0.20]
Basu et al. (2019)	17	61	0.28	[0.17; 0.41]
Basu et al. (2021)	6	18	0.33	[0.13; 0.59]
Narayanasamy et. al (2021)	7	256	0.03	[0.01; 0.06]
**Random effects model**		**4100**	**0.19**	**[0.13; 0.26]**
Heterogeneity: $I^2 = 90\%$, $\tau^2 = 1.4508$, $p < 0.01$				

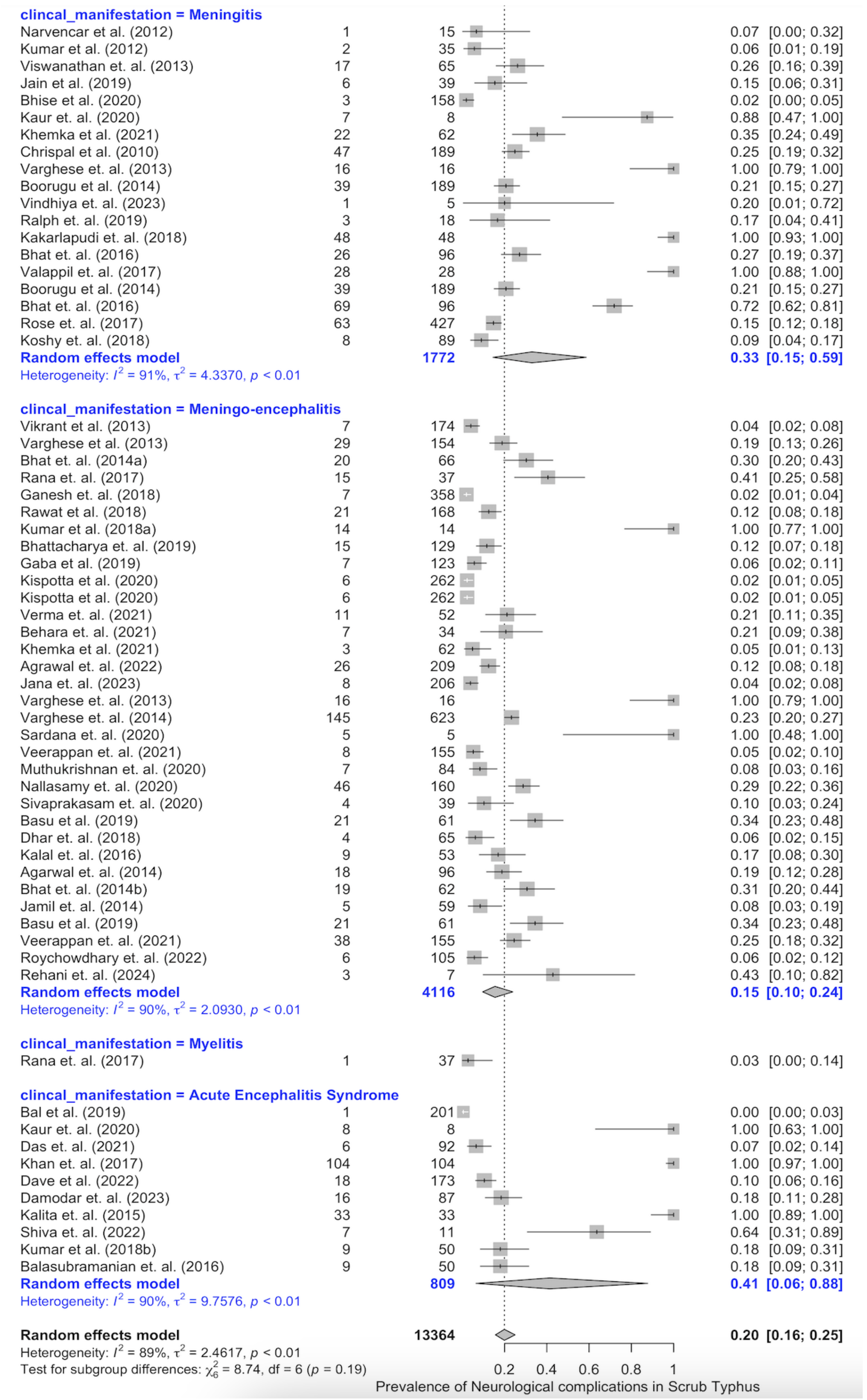

Forest plot 14. Pooled prevalence of different types of neurological complications of scrub typhus

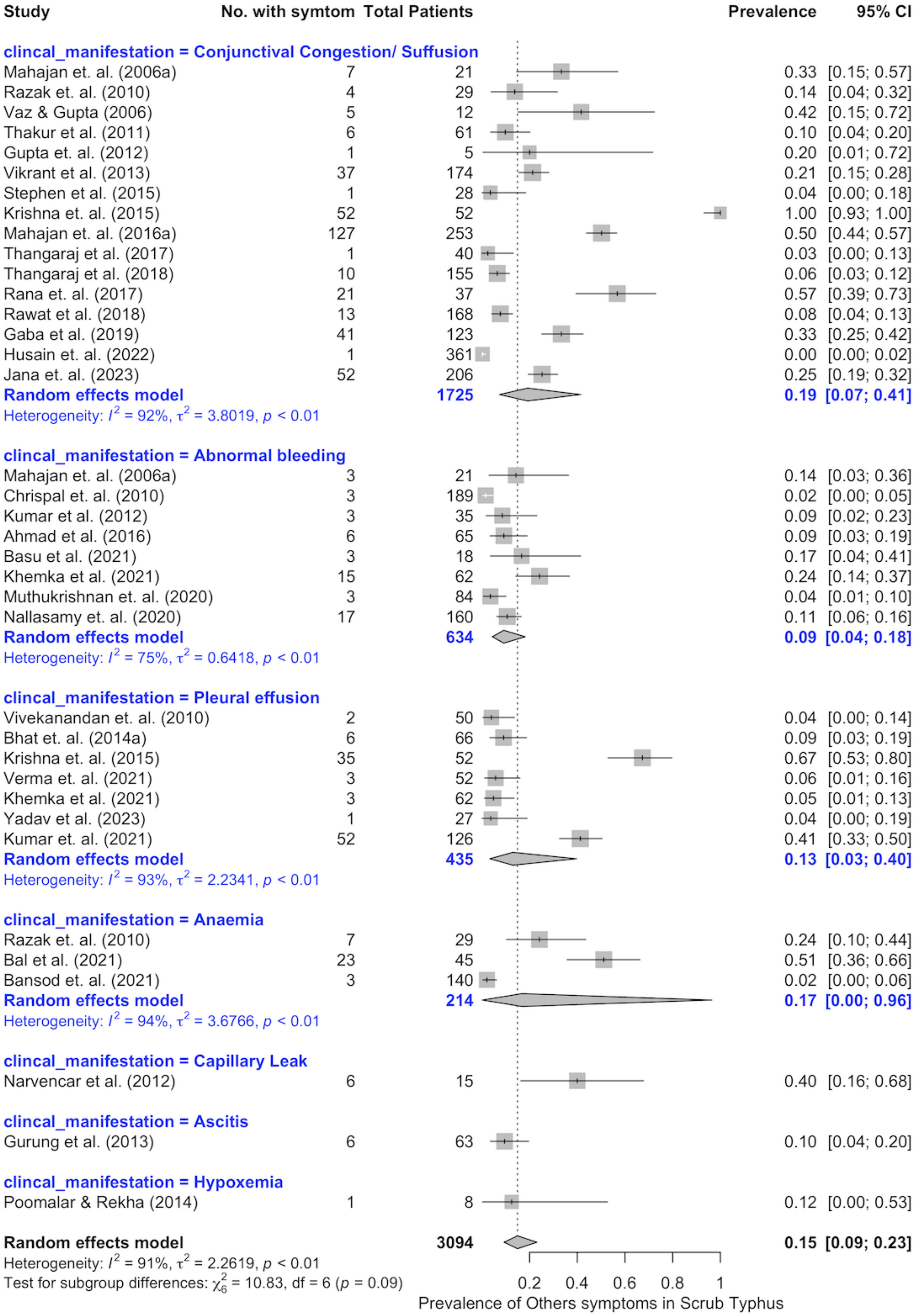

Forest plot 15. Pooled prevalence of other symptoms of scrub typhus

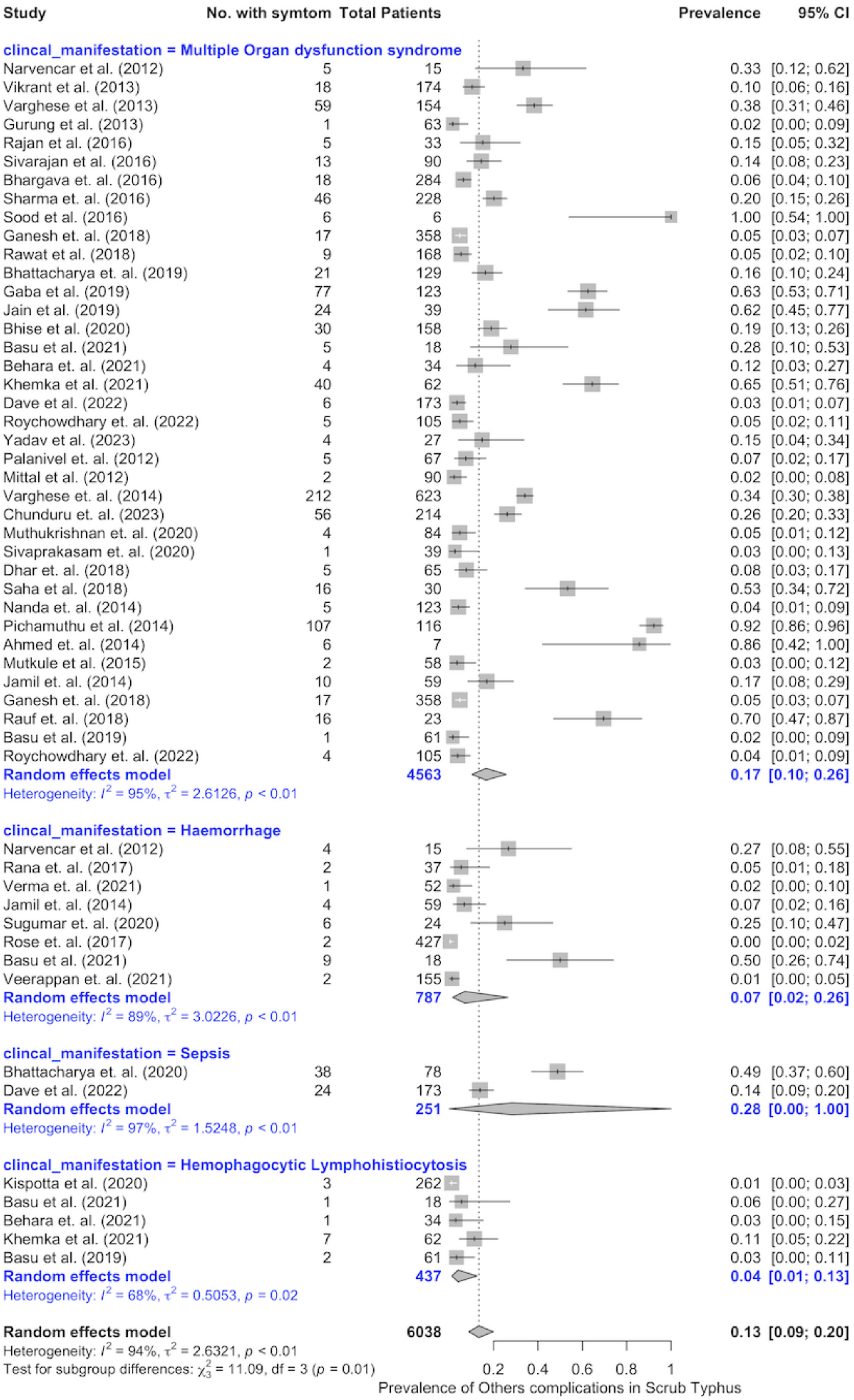

Forest plot 16. Pooled prevalence of other complications of scrub typhus

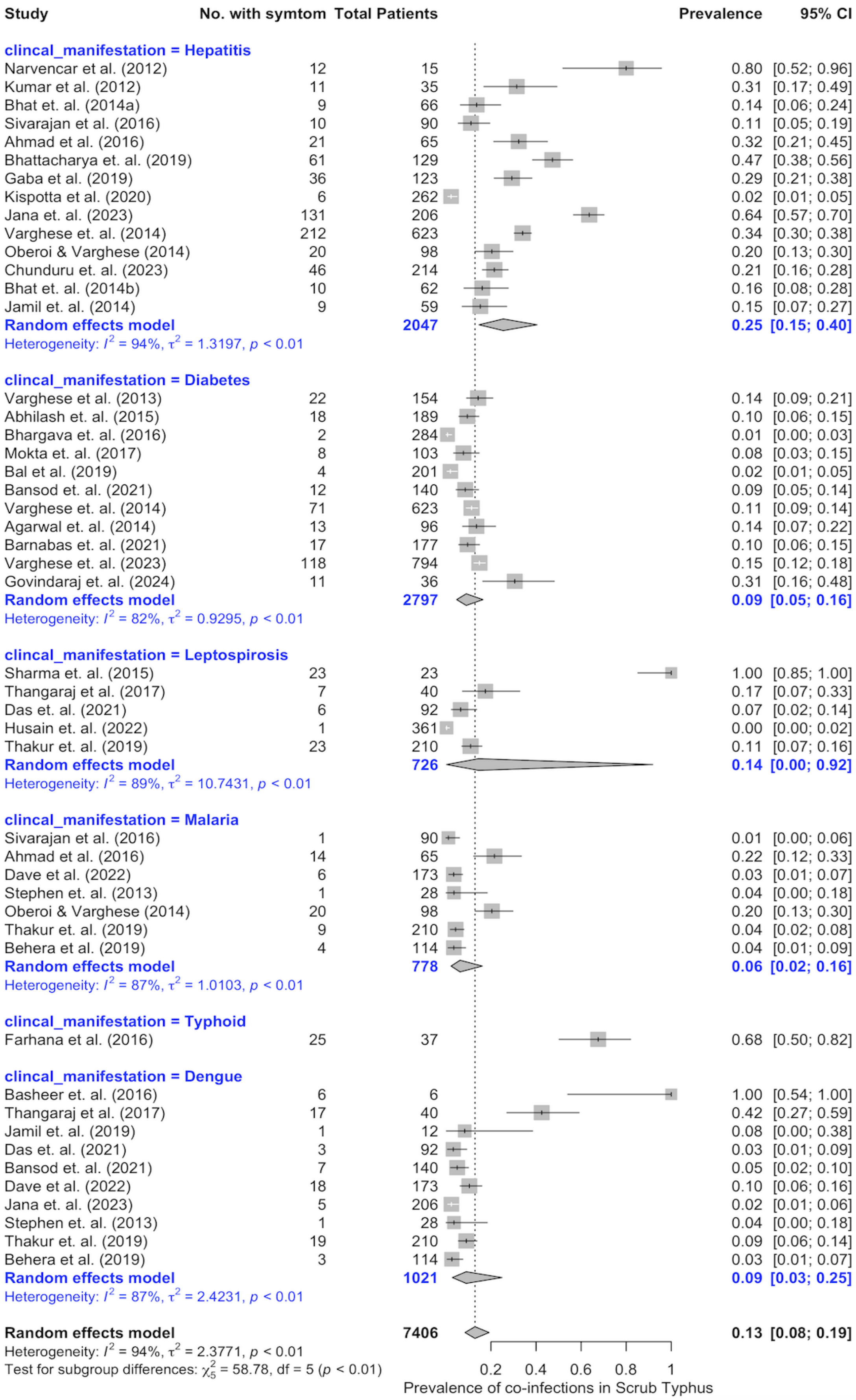

Forest plot 17. Pooled prevalence of co-infections along with scrub typhus reported in literature
